# Whole Genome Association Mapping of Plant Height in Winter Wheat (*Triticum aestivum* L.)

**DOI:** 10.1371/journal.pone.0113287

**Published:** 2014-11-18

**Authors:** Christine D. Zanke, Jie Ling, Jörg Plieske, Sonja Kollers, Erhard Ebmeyer, Viktor Korzun, Odile Argillier, Gunther Stiewe, Maike Hinze, Kerstin Neumann, Martin W. Ganal, Marion S. Röder

**Affiliations:** 1 Leibniz Institute of Plant Genetics and Crop Plant Research (IPK), Gatersleben, Germany; 2 TraitGenetics GmbH, Gatersleben, Germany; 3 KWS LOCHOW GMBH, Bergen, Germany; 4 Syngenta France S.A.S., Orgerus, France; 5 Syngenta Seeds GmbH, Bad Salzuflen, Germany; Institute for Sustainable Agriculture (IAS-CSIC), Spain

## Abstract

The genetic architecture of plant height was investigated in a set of 358 recent European winter wheat varieties plus 14 spring wheat varieties based on field data in eight environments. Genotyping of diagnostic markers revealed the *Rht-D1b* mutant allele in 58% of the investigated varieties, while the *Rht-B1b* mutant was only present in 7% of the varieties. *Rht-D1* was significantly associated with plant height by using a mixed linear model and employing a kinship matrix to correct for population stratification. Further genotyping data included 732 microsatellite markers, resulting in 770 loci, of which 635 markers were placed on the ITMI map plus a set of 7769 mapped SNP markers genotyped with the 90 k iSELECT chip. When Bonferroni correction was applied, a total of 153 significant marker-trait associations (MTAs) were observed for plant height and the SSR markers (−log_10_ (P-value) ≥4.82) and 280 (−log_10_ (P-value) ≥5.89) for the SNPs. Linear regression between the most effective markers and the BLUEs for plant height indicated additive effects for the MTAs of different chromosomal regions. Analysis of syntenic regions in the rice genome revealed closely linked rice genes related to gibberellin acid (GA) metabolism and perception, i.e. GA20 and GA2 oxidases orthologous to wheat chromosomes 1A, 2A, 3A, 3B, 5B, 5D and 7B, *ent*-kaurenoic acid oxidase orthologous to wheat chromosome 7A, *ent*-kaurene synthase on wheat chromosome 2B, as well as GA-receptors like DELLA genes orthologous to wheat chromosomes 4B, 4D and 7A and genes of the GID family orthologous to chromosomes 2B and 5B. The data indicated that besides the widely used GA-insensitive dwarfing genes *Rht-B1* and *Rht-D1* there is a wide spectrum of loci available that could be used for modulating plant height in variety development.

## Introduction

Plant height (PH) is an intrinsic component of plant architecture with effects on lodging stability, harvest index and yield. The introduction of dwarfing genes was a major factor during the ‘Green Revolution’ in the 1970 s, which led to a dramatic increase of production in the staple crops rice, wheat and maize at reduced costs [1], [2].

In wheat, the major dwarfing genes are *Rht-B1* and *Rht-D1* (old nomenclature *Rht1* and *Rht2*), which reduce plant height by reducing the response to gibberellin (gibberellin insensitive dwarfing genes) with pleiotropic effects on grain number and yield. The molecular identification of the *Rht-B1* and *Rht-D1* genes in wheat [3] confirmed them as orthologs to the gibberellin response regulator *GAI* in Arabidopsis [4] and the dwarfing genes *dwarf-8* (*d8*) and *dwarf-9* (*d9*) in maize [5]. Mutations in the DELLA-domain of these proteins often cause a dominant gibberellin-insensitive phenotype by increasing the stability of this negative regulator of gibberellin signal transduction [6]. Other orthologous DELLA genes are the *Slender* rice 1 [7] and the barley Slender1 [8], [9] mutants. The gibberellin-insensitive dwarf mutant *Dwarf 1* in rice encodes the α-subunit of GTP-binding protein which may be associated with gibberellin signal transduction [10]. The *GID*1 family in rice encodes a soluble receptor for gibberellin [11], while the *gid2* mutant is caused by the loss of function of a putative F-box protein [12].

Besides the gibberellin insensitive dwarfing genes also gibberellin sensitive dwarfing genes exist. A prominent example is the semi-dwarf (sd-1) “green revolution” rice, which contains a defective gibberellin 20-oxidase gene [13], [14]. Mutations in *GA20ox1* also caused semi-dwarf phenotypes in Arabidopsis [15] and an orthologous gene is likely to cause the *sdw1*/*denso* mutant in barley [16]. Like the gibberellin 20-oxidase mutants, the maize *Dwarf3* gene, encoding a protein of the cytochrome P450 superfamily, is involved in gibberellin biosynthesis [17].

A different mechanism for height reduction was described in the maize *brachytic2* (*br2*) mutants, where the loss of a P-glycoprotein modulated polar auxin transport in the stalk [18]. An orthologous mutant *dwarf3* was discovered in sorghum and the ABCB1 P-glycoprotein is a likely candidate for the *d2* dwarfing gene in pearl millet [19].

In wheat, besides the dwarfing genes with known function, e.g. *Rht-B1* and *Rht-D1* [20], a number of additional dwarfing genes were mapped in bi-parental populations [21]–[23]. In the current study, we were interested to gain a comprehensive picture about the loci involved in the regulation of plant height in European winter wheat, by applying a whole genome association mapping approach. Association mapping can be carried out with candidate genes [24], [25] or with anonymous markers covering the genome [26], [27]. In fact, the associations of the maize *Dwarf8* polymorphisms with variation in flowering time and plant height were regarded as first association mapping approach reported for crop plants [28], though the validity of this assumption recently has been questioned [29]. In comparison to bi-parental mapping populations, association panels have the advantage that the genetic diversity of larger germplasm panels can be monitored and a refined resolution can be obtained by monitoring the “historical” recombinations, which occurred during evolution or breeding of a line [30]. Association mapping and genomic selection approaches for plant height were conducted in barley [31], sorghum [32], maize [33] and wheat [34].

The goals of the current study were (i) to establish marker-trait associations (MTAs) for plant height in a panel of European winter wheat varieties, (ii) to compare the MTAs obtained with SSR markers to those obtained with SNP markers, and (iii) to connect the obtained MTAs to known genes of the gibberellic acid (GA) metabolism by exploiting the synteny to the annotated genome sequence of rice [35]. Our wheat association panel previously was analyzed for resistance to fungal pathogens [36]–[38] and heading date [39].

## Materials and Methods

### Plant material and phenotyping

No specific permits were required for the described field trials. The field trials were performed by companies. The field trials did not involve endangered or protected species. The plant material, consisting of 358 European winter wheat varieties plus 14 spring wheat varieties as a outgroup, is described in more detail in [36]. Field trials were conducted in the season 2008/2009 in Andelu/France (09.AND), Seligenstadt/Germany (09.SEL) and Wohlde/Germany (09.WOH) and in the season 2009/2010 in Andelu/France (10.AND), Janville/France (10.JAN), Saultain/France (10.SAU), Seligenstadt/Germany (10.SEL) and Wohlde/Germany (10.WOH) by applying an alpha design with two replications per site. Plot sizes were 5 to 6.75 m^2^ and climatic factors are described in Table S1. Both winter and spring varieties were sown in autumn and common agricultural practices were applied, which included the applications of growth regulators (Cycocel CL at a rate of 2.5L/ha 09.AND. 10.AND, 10.JAN and 10.SAU; CCC720 at a rate of 1.5L/ha at 09.WOH and 10.WOH; 1.0 L/ha CCC720 and 0.3 L/ha Moddus at 09.SEL and 1.3 L/ha CCC720 at 10.SEL). The active substance for CCC720 is Chlormequatchlorid (720 g/L), for Moddus it is Trinexapacethyl (250 g/L) and for Cycocel CL it is Chlormequat (460 g/L) and Imazaquine (10 g/L). Cycocel is based on the action of chlormequat chloride which is an inhibitor of *ent*-kaurene synthase involved in the early steps of GA-biosynthesis [40]. As untreated control, all varieties were grown in a nursery in rows with 6 plants in Gatersleben 2012 (12.GAT). Average plant height of each plot was measured before harvest with exclusion of awns.

### Molecular data analysis and genetic mapping

For marker-trait analysis a set of 732 microsatellite markers, resulting in 770 different loci spred across all chromosomes of wheat was used. Of these 770 loci, 635 loci were mapped and 135 loci were unmapped. Since the microsatellites are multi-allelic, they amounted to 3176 alleles. More details about this data set and the description of linkage disequilibrium (LD) and population structure are provided in [36]. For SNP-analysis, all 372 varieties were genotyped on a novel 90 k Infinium chip (90k iSELECT) [41], [42]. This resulted in a total of 21742 scorable and polymorphic markers on our association panel by considering all polymorphic markers with a minor allele frequency (MAF) >0.03. Of these markers, only the 7769 mapped markers were included in the association analysis, while the unmapped markers were not used for association analysis. The SSR-markers were mapped on the ITMI-population (International Triticeae Initiative) based on recombinant inbred lines between the parents W7984 (synthetic wheat) × Opata M85 [43], [44], while the SNP markers were mapped on 138 lines of a newly created doubled-haploid population of the same parents [45], [46]. Map construction was performed using the software package Joinmap 4.1. Both maps have different recombination values, and currently only few common markers are available, which makes comparisons difficult. For display a reduced version of the SNP-map was used containing most relevant markers with MTAs for PH. As candidate genes the photoperiodism gene *Ppd-D1* [47] and the dwarfing genes *Rht-B1* and *Rht-D1* [48] were genotyped on all varieties.

### Statistical analysis and association mapping

Each year-location combination was considered as an environment in our study. For each environment, adjusted entry mean of the genotypes were estimated using GenStat 13^th^ edition and following model:
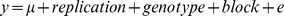
with replication and genotype as fixed factors and block as random effect nested within replication; µ represents an overall mean and e is a residual term; y represents the single plot values within each environment.

Adjusted entry means were used to calculate Best Linear Unbiased Estimators (BLUEs) across all eight environments using the software package GenStat 14^th^ edition [VSN International, Hemel Hempstead, Hertfordshire, UK] as described in [36]:
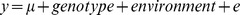
with genotype and environment as fixed effects; µ represents an overall mean and e is a residual term.

For calculating genotype-phenotype associations a minor allele frequency (MAF) threshold of 3% (equalling 11 varieties) was used for all markers. A mixed model for association mapping was applied based on the BLUEs across environments using the software package GenStat 14^th^ edition as described in [36] by applying a kinship matrix as relationship model:
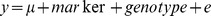



with 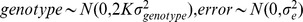.

Marker refers to a fixed effect of every marker, µ represents an overall mean, e is a residual term, and K denotes the kinship matrix among all genotypes. The Loiselle kinship matrix was calculated for 155 SSR markers, equally distributed on the genome, by using the software package SPAGeDi [49]. This kinship matrix was applied to correct for false positives when calculating MTAs with SSR as well as with SNP markers as described by [50]. The threshold of Bonferroni correction for multiple testing was calculated by dividing P<0.01 with the number of SSR or SNP markers used for the analysis. Marker effects (r^2^) were estimated using the software package TASSEL 3.0. The additive effects were calculated by using GenStat 14^th^ edition, where for the bi-allelic SNP-markers the most frequent alleles was set to zero and the difference of the phenotypic effect towards the less frequent allele was calculated. Therefore negative additive effects mean a decrease in plant height and positive additive effects mean an increase in plant height.

Spearman rank order correlations and ANOVA using the adjusted means of the eight environments were calculated with the software package SigmaPlot 11.0. The broad sense heritability was calculated from the variance components according to the formula:
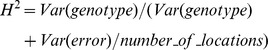
with variance components calculated with the software package SPSS v. 19. This software was also used to conduct a trait Posthoc test according to Tukey B.

## Results

### Description of phenotypic data

**Figure 1:**
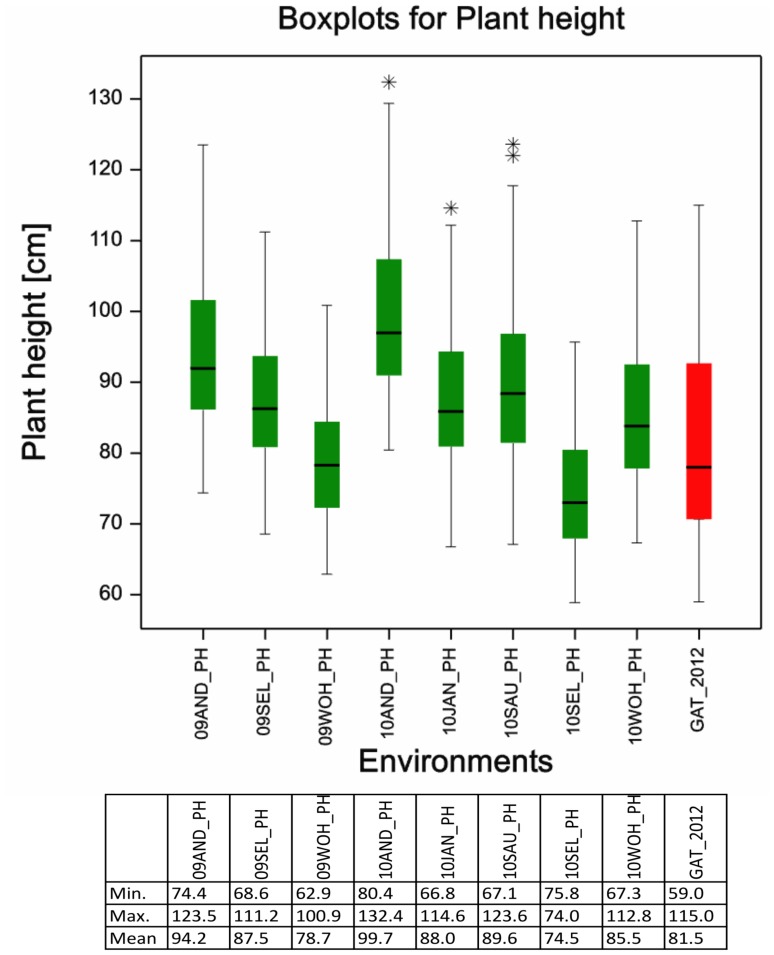
Boxplots for plant height. Boxplots for eight field environments are depicted in green, the untreated control in a nursery is shown in red. Asterisks mark outlier varieties.

**Figure 2:**
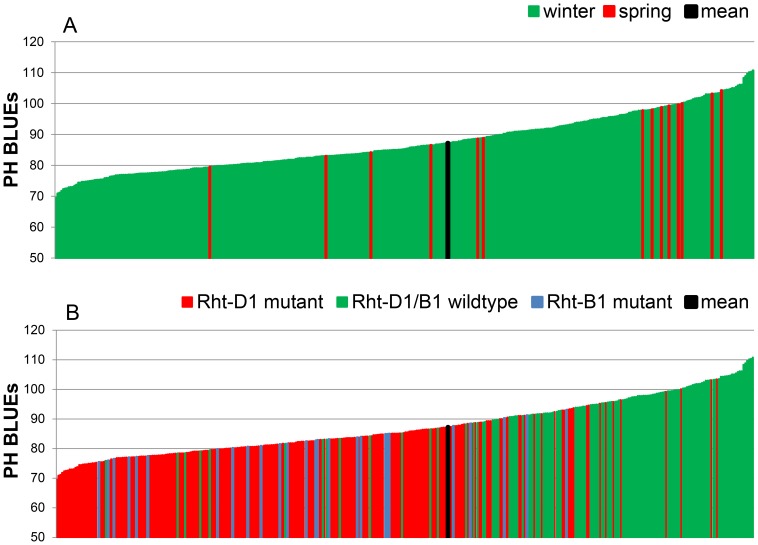
Phenotypic distribution of plant height BLUEs in 372 wheat varieties. The BLUEs for plant height were calculated across eight environments. Plant height BLUEs were arranged according to growth habit (A) or the distribution of the mutant alleles of the dwarfing genes *Rht-B1* and *Rht-D1* (B).

The phenotypic data were based on eight field environments with various distributions of the trait plant height (Figure 1). The BLUEs for PH across the eight environments ranged from 69.5 cm to 110.7 cm with a mean of 87.2 (Table S2; Figure 2). The untreated control 12.GAT had very similar values ranging from 59.0 cm to 115.0 cm (Figure 1). The broad sense heritability among the eight field environments was 0.89. An ANOVA test revealed a significant effect of environment and a Tukey B test discriminated seven phenotypic classes (Table S3). The Spearman rank order correlations among the eight field environments ranged from 0.862 to 0.943 indicating a good reproducibility of the ranking of the varieties in the different environments (Table S4). The correlation between the BLUEs of the eight field environments and the untreated control 12.GAT was 0.882. The analysis of variance was significant for genotype as well as environment (Table S5).

### Analysis of marker trait associations

**Figure 3:**
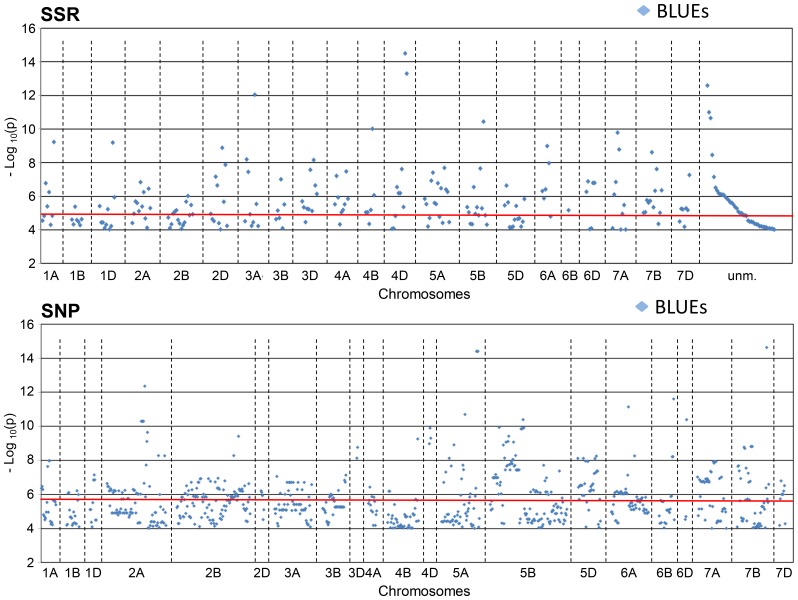
Manhattan plots of SSR or SNP marker alleles associated with plant height. The plot presents significant MTAs at a threshold of –log_10_ (P-value) ≥4.0 for BLUEs sorted according to their chromosomal location. The red line indicates the threshold for Bonferroni correction, –log_10_ (P-value) ≥4.82 (SSR) and ≥5.89 (SNP).

For analysis of MTAs, the eight field environments and the BLUEs across these eight environments were taken into account. By considering all MTAs found for all eight environments with −log_10_ (P-value) ≥4.0, in total 1873 MTAs were found for the SSRs and 6245 for the SNPs, of those 239 MTAs were detected for calculating with BLUEs across all environments in the SSRs, and 788 MTAs detected in the SNPs (Table 1; Tables S6, S7). After applying Bonferroni-correction, −log_10_ (P-value) ≥4.82 was significant for the SSRs and −log_10_ (P-value) ≥5.89 was significant for SNPs. Then the numbers shifted from 1104 significant MTAs for SSRs associated with BLUEs to 153 MTAs, and from 1904 significant MTAs for the SNPs associated with BLUEs to 280 MTAs (Figure 3). However, many markers detected MTAs in several environments and also especially the significant SNPs co-segregated or were closely linked in many cases, so that the number of real loci was significantly lower (Figure S1). By considering significant MTAs for BLUEs only and by combining marker loci with distance in the genetic map of ≤6 cM, the number of combined marker loci was estimated to be 109 for the SSRs and 87 for the SNPs (Tables S6B, S7B, S8).

**Table 1:** Number of MTAs per environment for the SSR markers and the SNPs of the 90K iSelect chip.

	SSR		90K iSelect	
Environments	−log_10_ (P-value) ≥4.0	−log_10_ (P-value) ≥4.82	−log_10_ (P-value) ≥4.0	−log_10_ (P-value) ≥5.89
Andelu (2009)	209	127	660	176
Seligenstadt (2009)	225	132	761	229
Wohlde (2009)	220	118	686	195
Andelu (2010)	221	130	705	273
Janvielle (2010)	169	87	566	134
Saultain (2010)	163	105	620	154
Seligenstadt (2010)	208	120	707	229
Wohlde (2010)	219	132	752	234
BLUEs	239	153	788	280
**Sum**	**1873**	**1104**	**6245**	**1904**

### Analysis of candidate genes

The *Rht-D1b* mutant allele on chromosome 4D was detected in 216 varieties, while only 26 *Rht-B1b* mutant alleles on chromosome 4B were present (Table S2). No double-dwarfs were found. In the spectrum of varieties the *Rht-D1b* and *Rht-B1b* mutant alleles were mainly represented in the first half of the distribution containing the shorter varieties (Figure 2). The MTA with *Rht-D1* candidate gene was highly significant in all environments (Table S9). No significant MTAs with −log_10_ (P-value) ≥3.0 were detected for *Rht-B1*, which may be due to the low representation of the mutant allele in the germplasm.

The varieties were also genotyped for the *Ppd-D1* gene on chromosome 2DS regulating the photoperiodic sensitivity of the lines [46]. The mutant allele *Ppd-D1a* rendering a photoperiod insensitive phenotype was detected in 53 varieties, which mostly originated from South France. The *Ppd-D1* candidate gene was significantly associated with plant height in all environments. A possible reason is that *Ppd-D1* is closely linked to the dwarfing gene *Rht8* [51]. Allelic variants of microsatellite marker GWM261 were reported as diagnostic for *Rht8* [22], [52]. The 192-bp allele of GWM261, which is considered to be diagnostic for reduced height at the *Rht8* locus, was present in 35 varieties (Table S2). Moderately significant MTAs for PH were found with −log_10_ (P-value) ≥3.0 in four environments and with −log_10_ (P-value) ≥4.0 in two environments (Table S9) for this marker. However, no linkage disequilibrium based on r^2^ was detected between the *Ppd-D1* candidate gene and marker GWM261, indicating that the significant MTA detected for *Ppd-D1* is independent of the effect based on *Rht8* (Figure S2). The Spearman rank order correlation between BLUEs for PH and BLUEs for heading date in the same environments was moderately significant with r = 0.158 (P = 0.00231).

### Additive effects of SSR and SNP markers

The varieties carried between 19 and 170 PH reducing SSR alleles and between 4 to 101 PH promoting alleles (Figure S3). The Spearman Rank order correlation between the number of PH reducing alleles per variety and the PH BLUEs was −0.750 (P = 0.0000002), and for the number of PH promoting alleles it was 0.808 (P = 0.0000002).

**Figure 4:**
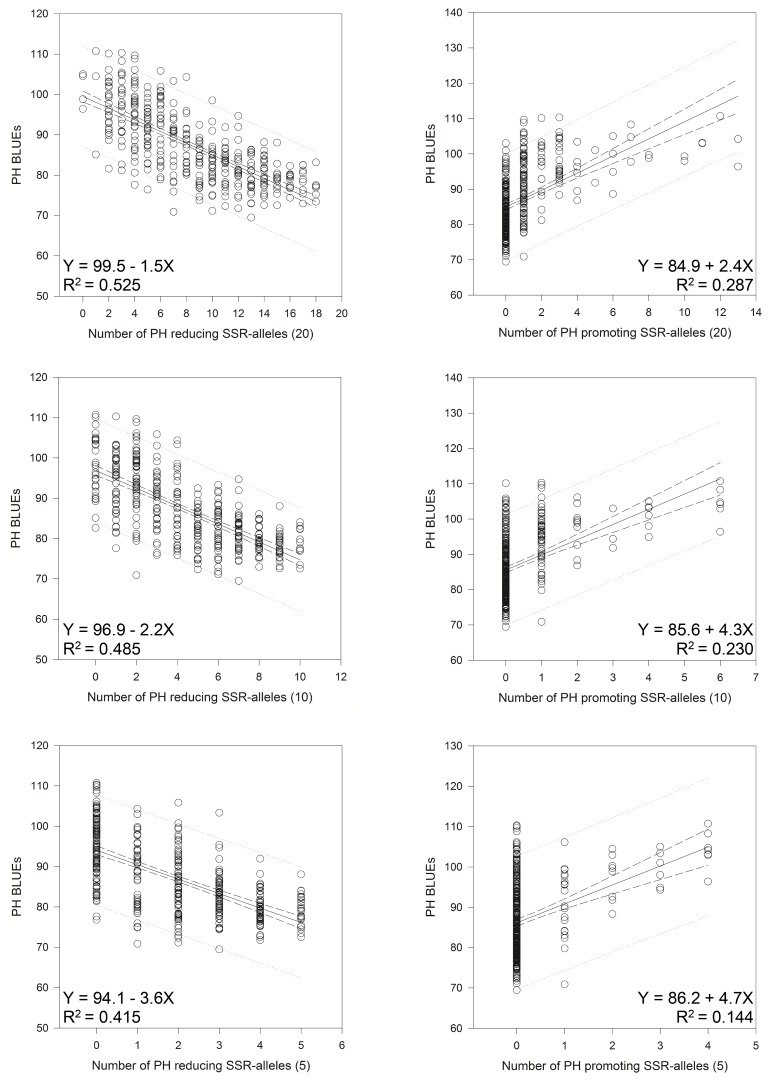
Linear regressions of the most PH reducing (“best”) and the most PH promoting (“worst”) SSR-alleles with PH-BLUEs. Linear regression resulted in a relationship between PH-BLUEs and the 20, 10 or 5 “best” or “worst” SSR-alleles in 372 varieties.

**Figure 5:**
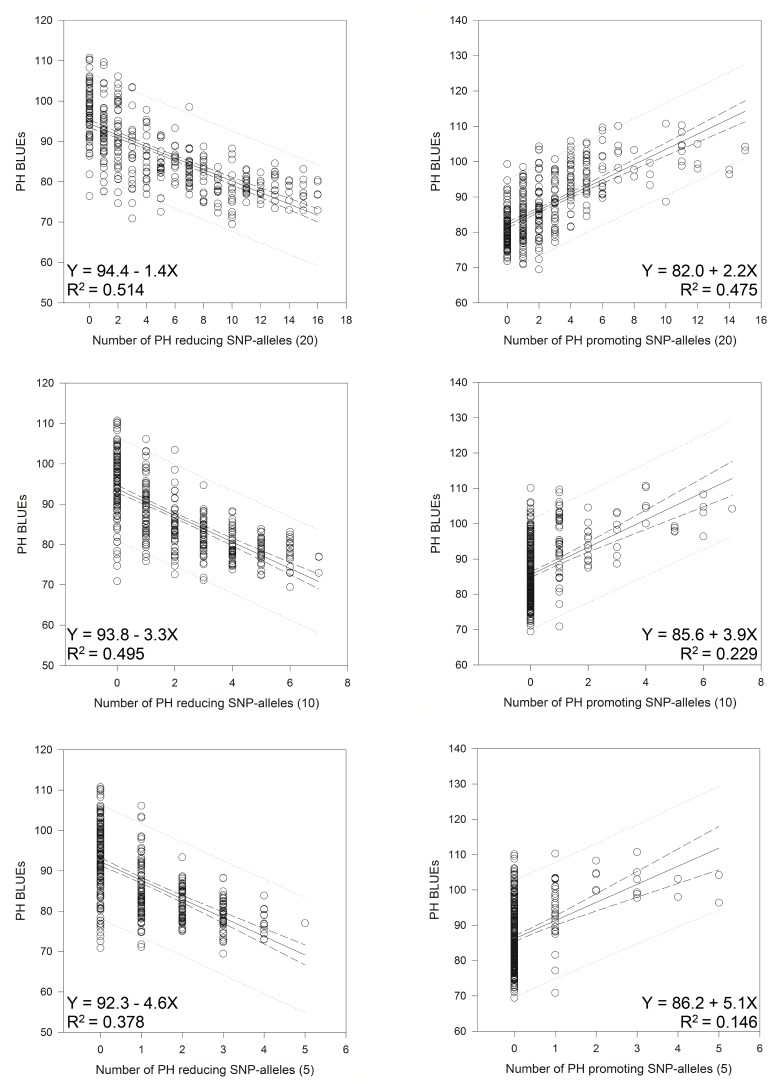
Linear regressions of the most PH reducing (“best”) and the most PH promoting (“worst”) SNP-alleles with PH-BLUEs. Linear regression resulted in a relationship between PH-BLUEs and the 20, 10 or 5 “best” or “worst” SNP-alleles from different loci in 372 varieties.

We sorted the significant SSR alleles with −log_10_ (P-value) ≥4.0 according to their additive effects and tested the 20, 10 and 5 most significant alleles for their effects on the PH BLUEs (Table 2). Linear regression showed a dependence of the PH BLUEs from the number of PH reducing alleles with Y = 99.5–1.5X with R^2^ = 0.525; for the PH promoting alleles the regression formula was Y = 84.9+2.4X with R^2^ = 0.287 for the 20 most significant SSR-markers based on the additive effects (Figure 4). Even with only 5 alleles, an R^2^ = 0.415 was found for the PH reducing alleles. Similar observations were found for the SNP-markers, where the 20 “best” and “worst” markers were selected which represented different combined loci, i.e. cluster of markers in LD were represented by only one marker (Table 3, Figure 5).

**Table 2:** List of the most PH reducing (“best”) and most PH enhancing (“worst”) SSR-alleles.

Marker alleles	Chromosome (linked gene)	Position (cM)	alleles belong to the					
			20 best	10 best	5 best	20 worst	10 worst	5 worst
GWM0357_128	1A	92.3	x	x				
GDM0126_196	1B	124.9				x		
GWM4780b_140	1D	89.1	x					
GWM4795_195	1D	130.8	x					
GWM1053_134	2A	0.2				x		
WMC0522_204	2A	88.7				x		
WMC0154_179*	2B	0.4	x					
GWM1419_197	2D	118.4				x		
GWM0539_151	2D	136.0				x	x	
GWM1204_279	2D	140.4	x					
GWM0349_232	2D	192.8				x	x	x
GWM1235_122	2D	215.0	x	x				
GWM1507_135*	3A	61.0	x	x	x			
CFA2193_239	3A	155.5	x	x	x			
GWM0938_144	3B	176.5				x	x	x
GWM1564_235	3B	238.5				x		
GWM0383a_183	3D	131.1				x	x	x
WMC0262_188	4A (*KAO-B1*)	165.1	x					
BARC0163_161	4B	81.2				x	x	x
GWM0819_183*	4D (*Rht-D1*)	34.8	x					
GWM4346_219*	4D (*Rht-D1*)	41.5	x	x	x			
GWM4001a_211	4D	100.6	x	x	x			
BARC0100b_162*	5A	88.1				x		
CFA2163a_187	5A (*Rht9*)	163.3				x	x	
WMC0415b_176*	5B	97.6				x	x	x
GWM1475a_122*	5B	108.2	x	x				
BARC0107_187*	6A	93.5	x					
GWM1241_126*	6D	138.5				x	x	
GWM1207_206	7A	230.3				x		
GWM0400_137	7B	3.4				x		
BARC0267a_145	7B	36.0	x	x				
GWM1650a_191	7D	29.1				x	x	
GWM0885_172	7D	52.1				x		
BARC0149_148	unm.	13.1	x					
BARC1144_268	unm.	31.0				x	x	
CFD0045_177	unm.	36.0	x	x				
WMC0032_null	unm.	50.0	x					
WMC0054_162	unm.	51.4	x					
WMC0410_115	unm.	74.1	x	x	x			
WMJ3103b_164	unm.	113.1				x		

*Coincides with meta-QTL according to Griffiths et al. (2012) [54].

**Table 3:** List of the most PH reducing (“best”) and most PH enhancing (“worst”) SNP-alleles.

SNP-Marker	Chromosome (linked orthologous locus in rice)	Position (cM)	alleles belong to the					
			20 best	10 best	5 best	20 worst	10 worst	5 worst
BS00086680_51	1A	62.6	x	x	x			
Kukri_c54467_100	1A	65.9	x					
tplb0025b13_2687	1D	3.4				x	x	x
wsnp_Ex_c1358_2602235	1D	12.1				x	x	
wsnp_CAP8_c2677_1394934	2A	66.6				x		
BS00022896_51	2A	69.8	x					
RAC875_c38018_278	2A	69.8	x	x				
wsnp_CAP11_c1711_934478	2A (*OsGA 20 oxidase 2*)	95.8	x					
RAC875_c4609_1756	2A	105.3	x					
BS00023075_51	2A	117.2	x					
Ra_c23048_474	2B	66.0	x					
BobWhite_rep_c64068_241	2B	131.7				x		
Tdurum_contig91865_242	3A	38.3	x	x	x			
Kukri_c20889_526	3A (*OsGA2ox3* *)	61.4				x	x	
BobWhite_c19725_1329	3B	57.4				x	x	
RAC875_c403_2247	3B (*OsGA2ox2* *)	66.8				x	x	x
Jagger_c3839_60	3D	147.1	x					
IAAV7221	4B	2.2	x	x	x			
RAC875_c1357_860	4B	66.4				x		
RFL_Contig5341_816	4B	79.1				x	x	x
wsnp_BE444644A_Ta_2_1	5A	11.0	x					
wsnp_BF293620A_Ta_2_3	5A	57.7	x	x				
wsnp_Ex_c23795_33033959	5A	101.2				x		
RAC875_c25756_279	5B (*Os GA receptor GID1L2*)	50.5	x					
IAAV7207	5B	57.4				x	x	x
Excalibur_c5329_1335	5B	104.1				x		
wsnp_Ex_c65985_64188864	5D	27.9				x		
wsnp_Ex_c1278_2449191	5D	81.7	x	x				
BS00089597_51	5D (*OsGA20ox1* *)	166.2	x	x				
BS00036878_51	6A	57.0				x		
BS00084314_51	6B	42.8	x	x	x			
RAC875_rep_c71463_98	6B	95.7	x	x				
Kukri_c73802_205	6D	2.3				x	x	
Excalibur_c15844_1470	6D	19.3				x		
Ex_c101666_634	7B	42.9				x	x	
wsnp_Ex_c24376_33618864	7B	50.6				x		
BS00024215_51	7B	54.5	x					
Tdurum_contig42584_1190	7B	140.4	x	x	x			
Excalibur_c22419_460	7D	56.8				x	x	x
Ku_c884_791	7D	83.9				x		

*Nomenclature according to Sakamoto et al. 2004 [58].

### Analysis of syntenic regions in the rice genome

We developed a strategy to search for potential candidate genes for plant height regulation in the complete set of 21742 scorable wheat SNPs based on synteny to the annotated rice genome. For this purpose, a keyword search was conducted with keywords ‘gibberellin’, ‘auxin’, ‘cytochrome P450’, ‘kaurene’, ‘kaurenoic’ and ‘DELLA’ in the MSU rice database (http://rice.plantbiology.msu.edu/). The key words were based on gene ontologies of known dwarfing genes from the literature. We then used the BlastX-annotations in rice of the wheat SNP markers, as provided by [42], to relate the rice loci detected in the keyword search to the complete set of wheat SNPs. A total of 694 hits for all keywords were found in the MSU rice database (Table 4). Of those 190 hits were present, scorable and polymorphic in the data of the 90 k iSELECT chip and 44 hits were significant for PH-BLUEs with −log_10_ (P-value) ≥3.0 (Table 4, Table S10). The significant markers included 20 hits for ‘cytochrome P450’, 14 hits for ‘auxin’ and 10 hits for ‘gibberellin’. Most significant with −log_10_ (P-value) ≥11.0 was wheat marker Kukri_c82296_367 on chromosome 5B with synteny to rice locus LOC_Os09g37500, annotated as OsSAUR55, an auxin responsive SAUR gene family. The SAUR ( = small auxin-up RNAs) gene family in rice represents early auxin-responsive genes with largely unknown function [53]. Additionally, nine wheat markers with homology to auxin responsive factors on chromosomes 2B, 3A, 3B, 5A, 6A, 7A and 7D were significant. The significant hits for ‘gibberellin’ included a gibberellin response modulator protein on chromosome 5B and a chitin-inducible gibberellin-responsive protein on chromosome 3A (Table S10).

**Table 4:** Keyword search in rice for PH candidate genes.

	Keywords in rice				
	gibberrellin	auxin	cytochromeP450	kaurene,kaurenoic	DELLA
Number of hits in MSU rice database	84	177	421	9	3
Number of syntenic wheat SNPs present and polymorphic in the 90 k iSELECT chip	20	61	103	6	0
Number of wheat SNPs significant for PH-BLUEs with LOD >3.0	10	14	20	0	0

Out of 84 hits for the keyword ‘gibberellin’ in the rice database only 20 hits were present and polymorphic in our 90 k iSELECT chip. Therefore, we wanted to test if any rice-genes from the key-word search ‘gibberellin’ are linked to highly significant genomic regions in the wheat-genome. For this purpose, the syntenic rice loci were extracted from [42] for the significant wheat SNP-markers of Table S7B and this list was compared to the rice loci of the keyword search ‘gibberellin’. The results of coinciding genomic regions are depicted in Table 5 and Table S11. The list of genes included several GA20 and GA2 oxidases orthologous to wheat chromosomes 1A, 2A, 3A, 3B, 5B, 5D and 7B, and other genes involved in GA synthesis, like ent-kaurenoic acid oxidase orthologous to wheat chromosome 7A and *ent*-kaurene synthase orthologous to wheat chromosome 2B. But there are also potential GA-receptors, like DELLA-genes orthologous to wheat chromosomes 4B, 4D and 7A and genes of the GID family orthologous to wheat chromosomes 2B and 5B (Table 5, Figure S1).

**Table 5:** List of orthologous loci in rice with genes of the GA metabolism or related to dwarfing phenotypes.

Wheat SNPs, chromosomal position	Orthologous locus in rice	Gene annotation in rice
1A; 69.8 cM	LOC_Os05g34854	OsGA2ox4*; GA 20 oxidase 2
2A; 95.8–98.1 cM	LOC_Os04g55070	GA 20 oxidase 2
2B; 64.3 cM	LOC_Os07g44850	GA receptor GID1L2
2B; 75.7–85.1 cM	LOC_Os04g52230	Ent kaurene synthase
3A; 59–61.4 cM	LOC_Os01g55240	OsGA2ox3*; GA 2-beta dioxygenase
3B; 59.7–66.8 cM	LOC_Os01g66100	OsGA20ox2*; GA 20 oxidase 2
4B; 50.8 cM	LOC_Os03g49990	DELLA, SLR1
4D; 47 cM	LOC_Os03g49990	DELLA, SLR1
5B; 45.8–50.5 cM	LOC_Os09g28230	GA receptor GID1L2
5B; 95.4–96.2 cM	LOC_Os03g42130	GA 20 oxidase 2
5B; 165 cM	LOC_Os03g63970	OsGA20ox1*; GA 20 oxidase 1
5D; 163.9–167.8 cM	LOC_Os03g63970	OsGA20ox1*; GA 20 oxidase 1
7A; 1.6–5.7 cM	LOC_Os06g02019	OsKAO*; ent-kaurenoic acid oxidase (cytochrom P450)
7A; 80.3 cM	LOC_Os06g02019	DELLA, RGL3
7B; 68.6 cM	LOC_Os08g44590	GA 20 oxidase 2

*Nomenclature according to Sakamoto et al. 2004 [58]

## Discussion

**Figure 6:**
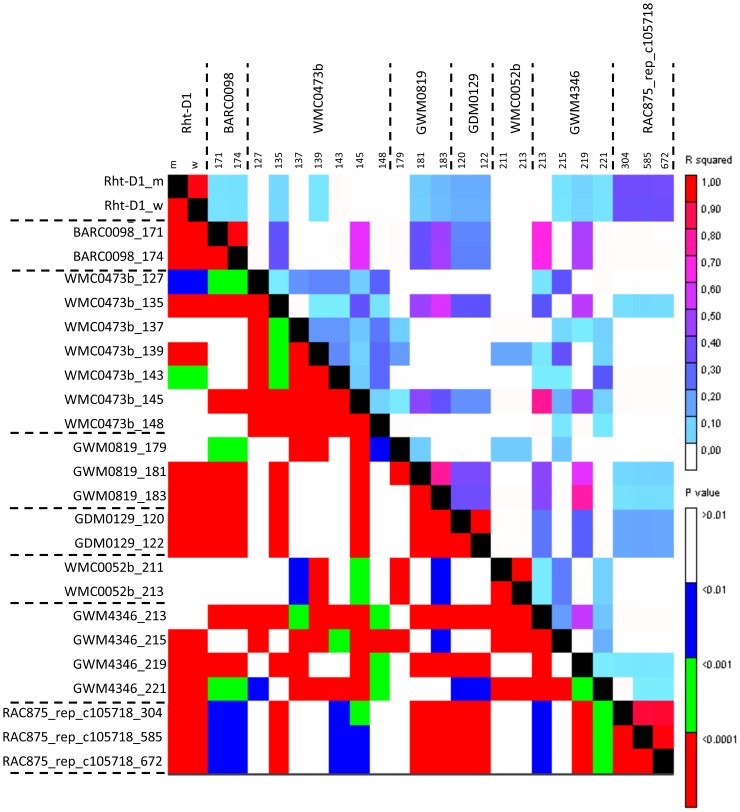
Linkage disequilibrium in the region of *Rht-D1*. The LD around *Rht-D1* on chromosome 4D extended to linked SSR and SNP markers with significant MTAs for plant height. In the upper triangle r^2^ and in the lower triangle the P-values are depicted.

The pattern of inheritance for plant height was characterized by many closely linked markers with high LD, especially for the SNPs. When these closely linked marker loci were combined, we estimated 109 loci for the SSRs and 87 for the SNPs. The higher numbers for SSRs may be caused by a better coverage of the D-genome for this marker type. Eventually, further loci could be combined. A meta-QTL analysis of four doubled-haploid populations in European elite winter wheat germplasm estimated sixteen meta-QTL loci [54]. Several of these meta-QTL coincided with our detected MTAs (Table 6). In the population Avalon × Cadenza, a QTL distal to marker GWM261 on chromosome 2DS was described [54], which may coincide with the MTAs detected by markers GWM4815 and GWM1418 in our study. This QTL could be due to the effect of various *Rht8* alleles [22], [52] or be a pleiotropic effect of *Ppd-D1* with a reduced time to flowering resulting in reduced plant height [55]. *Rht-D1* was included in a block of highly significant markers in LD including BARC98, WMC473b, GWM819, GDM129, GWM4346 for the SSRs and RAC875_rep_c105718_304 for the SNPs (Figure 6) and coincided with the respective meta-QTL on chromosome 4D [54]. Several of the markers coinciding with meta-QTL regions were found in our list of the 20 most PH reducing or PH promoting alleles based on the additive effects. These included besides the markers linked to *Rht-D1*, GWM4346 and GWM819, WMC154 on chromosome 2B, GWM1507 on 3A, BARC100 on 5A, WMC415b and GWM1475a on 5B, BARC107 on 6A and GWM1241 on chromosome 6D (Table 2).

**Table 6:** Coincidences of significant MTAs in our study with QTL or meta-QTL regions described by Griffiths et al. 2012 [54].

Significant markers in our study	Chromosome	Coincidences with QTL regions described in [54]
GWM164	1A	WMC336 - PSP3027
GWM337	1D	GWM337 - WMC36
GWM359	2A	wPt6207 - WMC827
WMC154	2B	Stm17tcac - WMC154
GMW4815, GWM1418	2D	CFD36 - GWM261
GWM1507, GWM4018	3A	BARC45 - WMC269
GWM4660, GWM251, BARC163	4B	GWM149 - GWM6
BARC98, WMC473b, GWM819, GDM129, GWM4346	4D	WMC285 - *Rht-D1*
BARC4, BARC100	5A	wPt3794 - GWM617
GWM1475, WMC415b, BARC109a, GWM1165, GWM1108, GWM335	5B	GWM213 - GWM408
BARC107, GWM4608, WMC672	6A	BARC23 - GWM570
GWM1241	6D	CFD42 - GWM325
GWM1397, GWM3062, GWM4335	7D	wPt6263 - GWM44

Of the known mapped *Rht*-genes in wheat, besides *Rht-D1* and *Rht8*, possibly an allelic effect of *Rht4* was discovered in our study. *Rht4* was mapped on chromosome arm 2BL with marker WMC317 explaining most of the phenotypic variance in the cross Vigour18 × Burt *ert* (*Rht4*) [23]. WMC317 was not significant in our association study, however significant MTAs were found for the flanking markers GWM1399, GWM619 and GWM3038 (Figure S1). The *Rht5*-gene was mapped on chromosome 3BS in the Vigour18 × Marfed M (*Rht5*) population and linked to marker BARC102. BARC102 is located in a distance of 1.2 cM (http://wheat.pw.usda.gov/GG2/index.shtml) from marker WMC808, which was significant in our study. Therefore the MTAs discovered by WMC808 and GWM4145 are possibly caused by an allele of *Rht5*. Gene *Rht9* was mapped to chromosome arm 5AL in the Chuan Mai 18 (*Rht8*) × Mara (*Rht8Rht9*) population and linked to marker BARC151 [23]. While BARC151 was not significant in our association panel, the neighboring markers GWM1342 and CFA2163 discovered highly significant MTAs (Figure S1), which may be an allelic effect of *Rht9*. The *Rht13* gene was reported to be linked to marker GWM577 on the distal long arm of chromosome 7B [23]. GWM577 was not significant in our association panel, but marker BARC182 in a distance of 12 cM was highly significant, an effect which may be caused by an allele of *Rht13*.

For a number of gibberellin metabolic pathway genes the chromosomal location is known in wheat [56]. Three *ent*-kaurenic acid oxidase (KAO) genes were mapped to the distal ends of chromosome arms 7AS, 4AL and 7DS corresponding to the 7BS/4AL translocation region [57]. The significant markers GWM681 and GWM735 correspond to the location of *KAO-A1* south of marker GWM471 on chromosome arm 7AS, while the significant markers WMC262 and GWM1251 correspond to *KAO-B1* on chromosome 4AL north of marker GWM160. WMC262 is listed among the 20 most effective SSR-markers for plant height reduction based on the additive effects (Table 2). The significant MTAs at both sites, on chromosomes 7AS and 4AL, may be caused by the respective *KAO*-genes, since a *KAO*-gene has been described as *Dwarf3* gene in maize [17]. On chromosome 7AS a large significant cluster of SNP markers was detected which had synteny to the rice gene *OsKAO* [58] on rice chromosome 6 (Table 5, Figure S1), the syntenic locus to *KAO-A1* in wheat.

Three GA 20-oxidase genes, *TaGA20ox1*, were located on the distal long arms of chromosomes 5B and 5D [59], [56] and chromosome arm 4AL in the region of the known 5AL/4AL translocation [60]. These loci may correspond to the significant MTAs detected in our study on chromosome arm 5DL by markers BARC177, WMC765, BARC144, GWM1454, GWM272, on chromosome arm 5BL by marker WMC 783 and on chromosome 4A by marker BARC 343. This assumption is supported by the fact that a cluster of SNP-markers on chromosome arm 5DL (wsnp_RFL_Contig2402_1924907, Excalibur_c24145_1643, wsnp_Ex_c11055_179228283, wsnp_Ex_c24145_33394561, wsnp_Ex_c24145_33394644) and an SNP marker on chromosome 5BL (Kukri_c1214_2565) spanned a region on rice chromosome 3, where *OsGA20ox1*, the syntenic locus to *TaGA20ox1*, is located (Table 5, Figure S1, Table S11).

Unfortunately, the 21742 scorable SNPs in our study did only include 20 hits for the keyword ‘gibberellin’ based on synteny to the rice genome (Table 4) and no GA20-oxidases were present. Therefore, we could only rely on indirect evidence based on mapping in similar genomic regions to assess the impact of gibberellin pathway genes (Table 5). Syntenic relationships to rice genomic regions containing GA oxidases were found for chromosomes 1A, 3A, 3B and 5B (Table 5). The regions on chromosomes 3A and 3B corresponded to *OsGA2ox3* and *OsGA20ox2* [58], both on rice chromosome 1. For both chromosomal regions large clusters of SNP markers were significant (Figure S1) and representative markers (Kukri_c20889_526 on chromosome 3A and RAC875_c403_2247 on chromosome 3B) were in the list of the makers with highest additive effects (Table 3). The semidwarfing gene *sdw1/denso* on chromosome 3HL in barley was described as most likely orthologous gene of the *sd1* gene in rice, which carries the mutation in the gene *OsGA20ox2* [16], [61] encoding an oxidase enzyme involved in the biosynthesis of gibberellin [13], [14]. Therefore, the highly significant SNP cluster on chromosome 3BL corresponding to *OsGA20ox2* in our study may be the syntenic locus to gene *sdw1/denso* in barley. Since co-location of loci is not a direct proof for the involvement of the mentioned genes in regulating PH, further sequence analysis and SNP-discovery in the wheat genes themselves is necessary to estimate their contribution to the expression of PH.

Syntenic relationships were also discovered for rice genomic regions carrying genes for GA perception, like DELLA proteins on wheat chromosomes 4B, 4D and 7A and GID receptors on wheat chromosomes 2B and 5B (Table 5). The cluster of SNP-markers on wheat chromosome 4D with synteny to *OsDELLA* on rice chromosome 3 corresponds to the *Rht-D1* gene, since the respective SNP-Markers (RAC875_rep_c105718_304, RAC875_rep_c105718_672, RAC875_rep_c105718_585) are in LD with *Rht-D1* (Figure 6).

The significant candidate genes of the keyword search (Table 4) included 20 hits for ‘cytochrome P450’. Cytochrom P450 is part of *ent*-kaurenoic acid oidase, an enzyme of the GA-metabolism, but it also is present in enzymes of other functions including the involvement in brassinosteroid biosynthesis [62]. Highly significant hits were found for auxin-related genes as well. These included markers with homology to auxin responsive factors and the SAUR gene family, the function of which is largely unknown in both cases [53], [63].

Our genotyping results indicated, that the *Rht-D1b* mutant allele on chromosome 4D was present in 58% of the varieties analyzed, while the *Rht-B1b* mutant allele on chromosome 4B was found in only 7% of our varieties. The detection method employed [48] did not distinguish from further alleles, such as *Rht-B1b* from *Rht-B1d*, and *Rht-D1b* from *Rht-D1c* and *Rht-D1d* [64], [65]. The high percentage of *Rht-D1*mutant allele in our germplasm set contradicted the results of [66], where 12% *Rht-B1b* and 16% *Rht-D1b* mutant alleles were reported in 103 French varieties, while in the total set of 368 worldwide accessions for both loci, *Rht-B1* and *Rht-D1*, a frequency of 12% mutant alleles was found. In contrast to our study, where only registered varieties were investigated, the study of [66] included landraces. Another study reported frequencies of 40% for *Rht-B1b* and 22% for *Rht-D1b* in a 172 genotypes originating from 20 countries [67]. The data indicated, that *Rht-D1b* may be prevalent in the Middle European wheat varieties, while *Rht-B1b* seems to occur mainly in the world wide germplasm including landraces.

The *Rht1* mutant phenotypes were found to increase harvest index and grain yield [68] and were therefore introduced in many commercial wheat varieties around the world. But also some negative effects, like decreased seedling vigour and shorter coleoptiles were reported for these GA-insensitive dwarfing genes [69], [70]. Beneficial effects for the *Rht1* wild type alleles were reported in drought environments [71]. Therefore, the use of alternative height reducing genes was suggested [70], [72]. Our data indicate a wealth of alternative loci that could be employed for modulating plant height reduction and which are already present in the germplasm. With only five SNP- or SSR-markers obvious additive effects for PH BLUEs were observed (Figures 4 and 5). Similar results were reported for pyramiding of elite alleles for plant height in two winter wheat populations [73].

## Supporting Information

Figure S1 **Chromosomal location of marker-trait associations for plant height.** The linkage map for the SNP-markers of the 90 k iSELECT chip based on the ITMI-DH population is depicted on the left side, while the linkage map for the SSR-markers based on the ITMI-RIL population is shown on the right side for each linkage group. Each MTA for a single environment or the BLUEs are depicted by an icon as explained on the first page of the file. The locations of known *Rht* genes and known mapping positions for gibberellin metabolism genes for wheat are indicated in the SSR maps. Orthologous sites to rice (*Oryza sativa* = *Os*) loci with genes related to GA metabolism or perception are indicated in the SNP maps.
(PDF)

Figure S2 **No Linkage disequilibrium was detected between SSR-marker GWM0261 and the candidate gene** ***Ppd-D1*** **on chromosome 2D.**
(PDF)

Figure S3 **Frequency of (A) PH reducing and (B) PH promoting SSR-alleles per variety**.
(PDF)

Table S1 **Climatic factors at trial sites.**
(XLSX)

Table S2 **List of varieties, genotyping data of candidate genes and phenotypic data.**
(XLSX)

Table S3 **Estimation of variance components and broad sense heritability and estimation of differences between groups ( = environments) using ANOVA and a Tukey B test.**
(DOCX)

Table S4 **Spearman rank order correlation of PH scores in 372 varieties among eight environments and the BLUEs.** GAT_2012 represents an untreated control environment in a plant nursery.
(DOCX)

Table S5 **Analysis of variance (ANOVA) of plant height in 372 varieties in eight environments.**
(DOCX)

Table S6 **List of significant (–log_10_(P) >4.0) marker trait associations of the SSR markers. Sheet 6A: List of significant (–log_10_(P) >4.0) marker trait associations of the SSR markers.** Columns F to U describe the additive effect and the R^2^ for each environment. Colum V indicates the R2 of the BLUEs values. Positive additive effects promote plant height and negative additive effects reduce plant height. **Sheet 6B:** **List of significant (–log_10_(P) >4.0) marker trait associations of the SSR markers for BLUEs only.** Markers with distances <6.0 cM were combined by colour coding. Columns F to U describe the additive effect and the R^2^ for each environment. Colum V indicates the R^2^ of the BLUEs values. Positive additive effects promote plant height and negative additive effects reduce plant height.
(XLSX)

Table S7 **List of significant (–log_10_(P) >4.0) marker trait associations of the SNP markers. Sheet 7A: List of significant (–log_10_(P) >4.0) marker trait associations of the SNP markers.** Columns G to V describe the additive effect and the R^2^ for each environment. Colum W indicates the R^2^ of the BLUEs values. Positive additive effects promote plant height and negative additive effects reduce plant height. **Sheet 7B: List of significant (–log_10_(P) >4.0) marker trait associations of the SNP markers for the BLUEs only.** Markers with distances <6.0 cM were combined by colour coding. Columns G to V describe the additive effect and the R^2^ for each environment. Colum W indicates the R^2^ of the BLUEs values. Positive additive effects promote plant height and negative additive effects reduce plant height.
(XLSX)

Table S8 **Number of MTAs per chromosomes for the SSR and SNP markers.**
(XLSX)

Table S9 **List of significant (–log_10_(P) >2.0) marker trait associations of the candidate genes.** Columns F to U describe the additive effect and the R^2^ for each environment. Colum V indicates the R^2^ of the BLUEs values. Positive additive effects promote plant height and negative additive effects reduce plant height.
(XLSX)

Table S10 **List of significant (−log_10_(P) >3.0) marker trait associations of the candidates from the keyword search.** Columns Y to AF describe the additive effect for each environment. Column AG indicates the additive effect of the BLUEs values. Positive additive effects promote plant height and negative effects reduce plant height.
(XLSX)

Table S11 **List of wheat SNP with GA related orthologous loci in rice.**
(XLSX)
