# Supplementary material for: Whole Genome Association Mapping of Plant Height in Winter Wheat (Triticum aestivum L.)
Source: PLoS One. 2014 Nov 18;9(11):e113287. doi: 10.1371/journal.pone.0113287 (PMC4236181; doi:10.1371/journal.pone.0113287)
Supplement: Figure S1 — Chromosomal location of marker-trait associations for plant height. The linkage map for the SNP-markers of the 90 k iSELECT chip based on the ITMI-DH population is depicted on the left side, while the linkage map for the SSR-markers based on the ITMI-RIL population is shown on the right side for each linkage group. Each MTA for a single environment or the BLUEs are depicted by an icon as explained on the first page of the file. The locations of known Rht genes and known mapping positions for gibberellin metabolism genes for wheat are indicated in the SSR maps. Orthologous sites to rice (Oryza sativa = Os) loci with genes related to GA metabolism or perception are indicated in the SNP maps. (PDF) [file pone.0113287.s001.pdf]

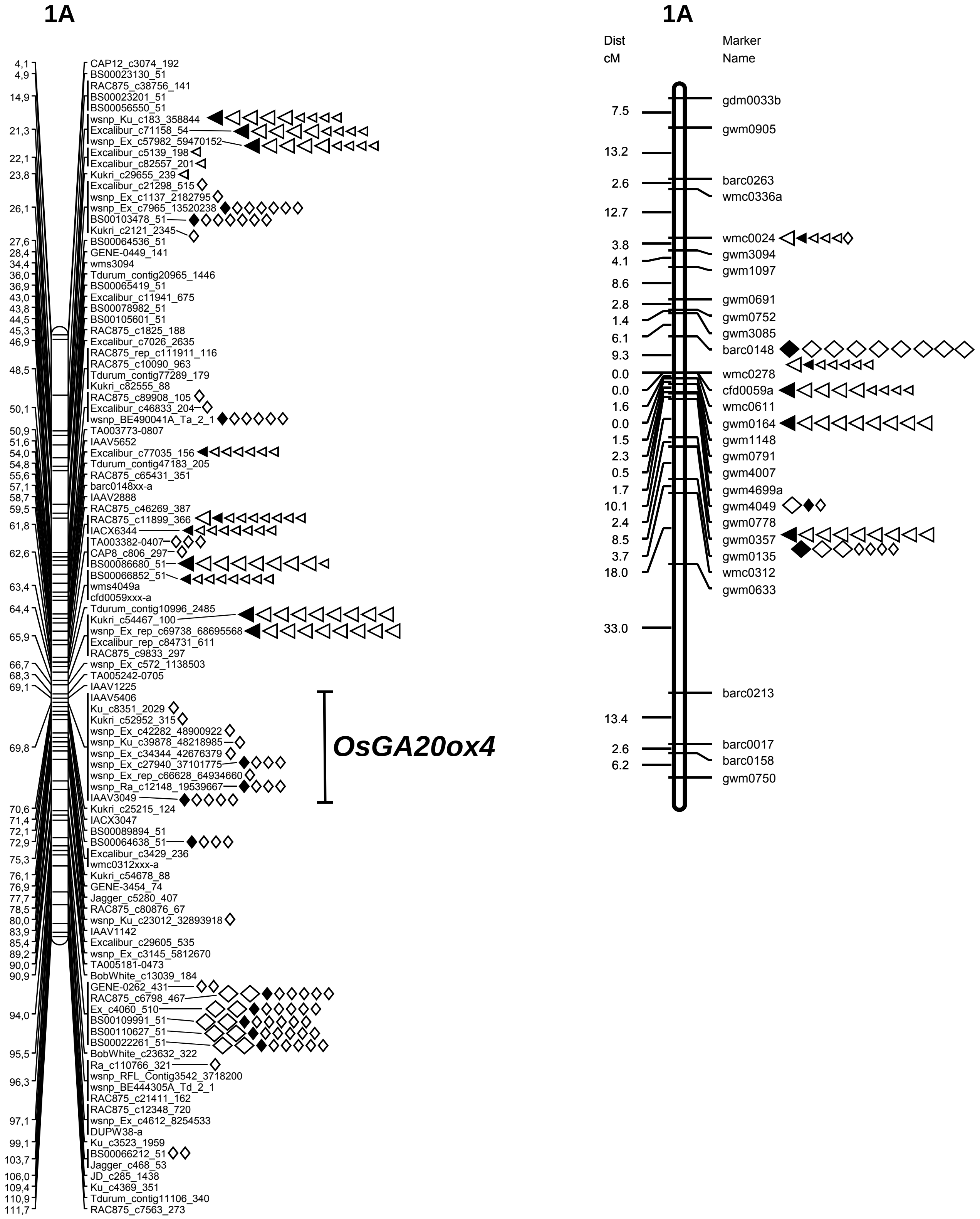

Fig S1: Chromosomal location of marker trait associations.

- ◁ Plant height decreasing effect, single environment, -$\log_{10}$(p)-value ≥ 4.0
- ◁ Plant height decreasing effect, single environment, -$\log_{10}$(p)-value ≥ 4.82 (SSR) ≥ 5.89 (SNP)
- ◀ Plant height decreasing effect, BLUEs, -$\log_{10}$(p)-value ≥ 4.0
- ◀ Plant height decreasing effect, BLUEs, -$\log_{10}$(p)-value ≥ 4.82 (SSR) ≥ 5.89 (SNP)

- ◇ Plant height increasing effect, single environment, -$\log_{10}$(p)-value ≥ 4.0
- ◇ Plant height increasing effect, single environment, -$\log_{10}$(p)-value ≥ 4.82 (SSR) ≥ 5.89 (SNP)
- ◆ Plant height increasing effect, BLUEs, -$\log_{10}$(p)-value ≥ 4.0
- ◆ Plant height increasing effect, BLUEs, -$\log_{10}$(p)-value ≥ 4.82 (SSR) ≥ 5.89 (SNP)

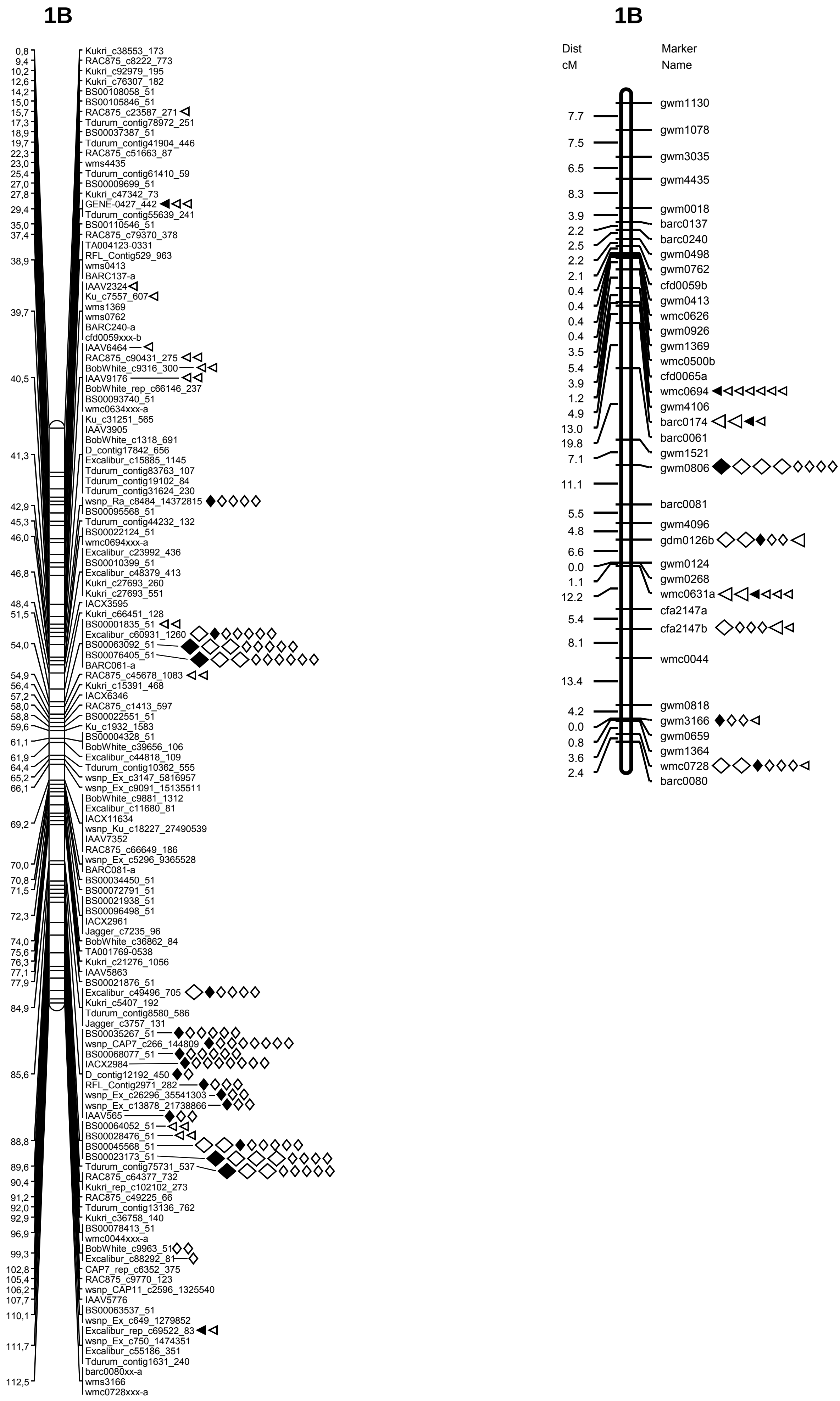
1B
0,8
9,4
10,2
12,6
14,2
15,0
15,7
17,3
18,9
19,7
22,3
23,0
25,4
27,0
27,8
29,4
35,0
37,4
38,9
39,7
40,5
41,3
42,9
45,3
46,0
46,8
48,4
51,5
54,0
54,9
56,4
57,2
58,0
58,8
59,6
61,1
61,9
64,4
65,2
66,1
69,2
70,0
70,8
71,5
72,3
74,0
75,6
76,3
77,1
77,9
84,9
85,6
88,8
89,6
90,4
91,2
92,0
92,9
96,9
99,3
102,8
105,4
106,2
107,7
110,1
111,7
112,5
Kukri_c38553_173
RAC875_c8222_773
Kukri_c92979_195
Kukri_c76307_182
BS00108058_51
BS00105846_51
RAC875_c23587_271
Tdurum_contig78972_251
BS00037387_51
Tdurum_contig41904_446
RAC875_c51663_87
wms4435
Tdurum_contig61410_59
BS00009699_51
Kukri_c47342_73
GENE-0427_442
Tdurum_contig55639_241
BS00110546_51
RAC875_c79370_378
TA004123-0331
RFL_Contig529_963
wms0413
BARC137-a
IAAV2324
Ku_c7557_607
wms1369
wms0762
BARC240-a
cfd0059xxx-b
IAAV6464
RAC875_c90431_275
BobWhite_c9316_300
IAAV9176
BobWhite_rep_c66146_237
BS00093740_51
wmc0634xxx-a
Ku_c31251_565
IAAV3905
BobWhite_c1318_691
D_contig17842_656
Excalibur_c15885_1145
Tdurum_contig83763_107
Tdurum_contig19102_84
Tdurum_contig31624_230
wsnp_Ra_c8484_14372815
BS00095568_51
Tdurum_contig44232_132
BS00022124_51
wmc0694xxx-a
Excalibur_c23992_436
BS00010399_51
Excalibur_c48379_413
Kukri_c27693_260
Kukri_c27693_551
IACX3595
Kukri_c66451_128
BS00001835_51
Excalibur_c60931_1260
BS00063092_51
BS00076405_51
BARC061-a
RAC875_c45678_1083
Kukri_c15391_468
IACX6346
RAC875_c1413_597
BS00022551_51
Ku_c1932_1583
BS00004328_51
BobWhite_c39656_106
Excalibur_c44818_109
Tdurum_contig10362_555
wsnp_Ex_c3147_5816957
wsnp_Ex_c9091_15135511
BobWhite_c9881_1312
Excalibur_c11680_81
IACX11634
wsnp_Ku_c18227_27490539
IAAV7352
RAC875_c66649_186
wsnp_Ex_c5296_9365528
BARC081-a
BS00034450_51
BS00072791_51
BS00021938_51
BS00096498_51
IACX2961
Jagger_c7235_96
BobWhite_c36862_84
TA001769-0538
Kukri_c21276_1056
IAAV5863
BS00021876_51
Excalibur_c49496_705
Kukri_c5407_192
Tdurum_contig8580_586
Jagger_c3757_131
BS00035267_51
wsnp_CAP7_c266_144809
BS00068077_51
IACX2984
D_contig12192_450
RFL_Contig2971_282
wsnp_Ex_c26296_35541303
wsnp_Ex_c13878_21738866
IAAV565
BS00064052_51
BS00028476_51
BS00045568_51
BS00023173_51
Tdurum_contig75731_537
RAC875_c64377_732
Kukri_rep_c102102_273
RAC875_c49225_66
Tdurum_contig13136_762
Kukri_c36758_140
BS00078413_51
wmc0044xxx-a
BobWhite_c9963_51
Excalibur_c88292_81
CAP7_rep_c6352_375
RAC875_c9770_123
wsnp_CAP11_c2596_1325540
IAAV5776
BS00063537_51
wsnp_Ex_c649_1279852
Excalibur_rep_c69522_83
wsnp_Ex_c750_1474351
Excalibur_c55186_351
Tdurum_contig1631_240
barc0080xx-a
wms3166
wmc0728xxx-a
1B
Dist
cM
Marker
Name
7.7
7.5
6.5
8.3
3.9
2.2
2.5
2.2
2.1
0.4
0.4
0.4
0.4
3.5
5.4
3.9
1.2
4.9
13.0
19.8
7.1
11.1
5.5
4.8
6.6
0.0
1.1
12.2
5.4
8.1
13.4
4.2
0.0
0.8
3.6
2.4
gwm1130
gwm1078
gwm3035
gwm4435
gwm0018
barc0137
barc0240
gwm0498
gwm0762
cfd0059b
gwm0413
wmc0626
gwm0926
gwm1369
wmc0500b
cfd0065a
wmc0694
gwm4106
barc0174
barc0061
gwm1521
gwm0806
barc0081
gwm4096
gdm0126b
gwm0124
gwm0268
wmc0631a
cfa2147a
cfa2147b
wmc0044
gwm0818
gwm3166
gwm0659
gwm1364
wmc0728
barc0080

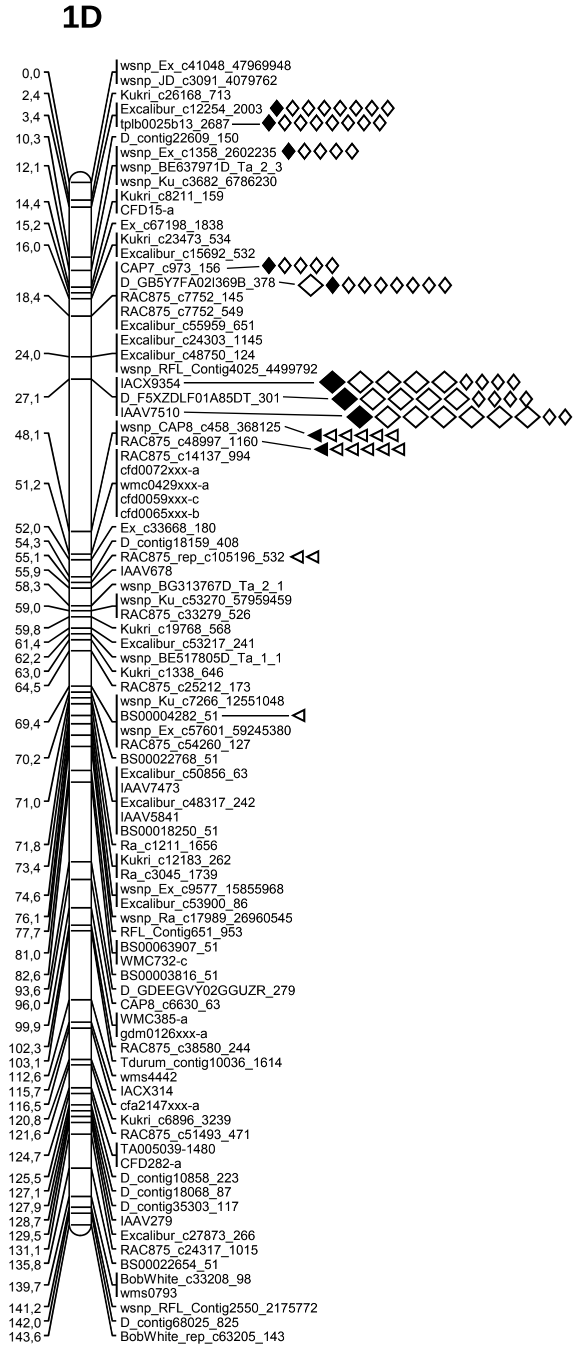
1D
0,0
2,4
3,4
10,3
12,1
14,4
15,2
16,0
18,4
24,0
27,1
48,1
51,2
52,0
54,3
55,1
55,9
58,3
59,0
59,8
61,4
62,2
63,0
64,5
69,4
70,2
71,0
71,8
73,4
74,6
76,1
77,7
81,0
82,6
93,6
96,0
99,9
102,3
103,1
112,6
115,7
116,5
120,8
121,6
124,7
125,5
127,1
127,9
128,7
129,5
131,1
135,8
139,7
141,2
142,0
143,6
wsnp_Ex_c41048_47969948
wsnp_JD_c3091_4079762
Kukri_c26168_713
Excalibur_c12254_2003
tplb0025b13_2687
D_contig22609_150
wsnp_Ex_c1358_2602235
wsnp_BE637971D_Ta_2_3
wsnp_Ku_c3682_6786230
Kukri_c8211_159
CFD15-a
Ex_c67198_1838
Kukri_c23473_534
Excalibur_c15692_532
CAP7_c973_156
D_GB5Y7FA02I369B_378
RAC875_c7752_145
RAC875_c7752_549
Excalibur_c55959_651
Excalibur_c24303_1145
Excalibur_c48750_124
wsnp_RFL_Contig4025_4499792
IACX9354
D_F5XZDLF01A85DT_301
IAAV7510
wsnp_CAP8_c458_368125
RAC875_c48997_1160
RAC875_c14137_994
cfd0072xxx-a
wmc0429xxx-a
cfd0059xxx-c
cfd0065xxx-b
Ex_c33668_180
D_contig18159_408
RAC875_rep_c105196_532
IAAV678
wsnp_BG313767D_Ta_2_1
wsnp_Ku_c53270_57959459
RAC875_c33279_526
Kukri_c19768_568
Excalibur_c53217_241
wsnp_BE517805D_Ta_1_1
Kukri_c1338_646
RAC875_c25212_173
wsnp_Ku_c7266_12551048
BS00004282_51
wsnp_Ex_c57601_59245380
RAC875_c54260_127
BS00022768_51
Excalibur_c50856_63
IAAV7473
Excalibur_c48317_242
IAAV5841
BS00018250_51
Ra_c1211_1656
Kukri_c12183_262
Ra_c3045_1739
wsnp_Ex_c9577_15855968
Excalibur_c53900_86
wsnp_Ra_c17989_26960545
RFL_Contig651_953
BS00063907_51
WMC732-c
BS00003816_51
D_GDEEGVY02GGUZR_279
CAP8_c6630_63
WMC385-a
gdm0126xxx-a
RAC875_c38580_244
Tdurum_contig10036_1614
wms4442
IACX314
cfa2147xxx-a
Kukri_c6896_3239
RAC875_c51493_471
TA005039-1480
CFD282-a
D_contig10858_223
D_contig18068_87
D_contig35303_117
IAAV279
Excalibur_c27873_266
RAC875_c24317_1015
BS00022654_51
BobWhite_c33208_98
wms0793
wsnp_RFL_Contig2550_2175772
D_contig68025_825
BobWhite_rep_c63205_143

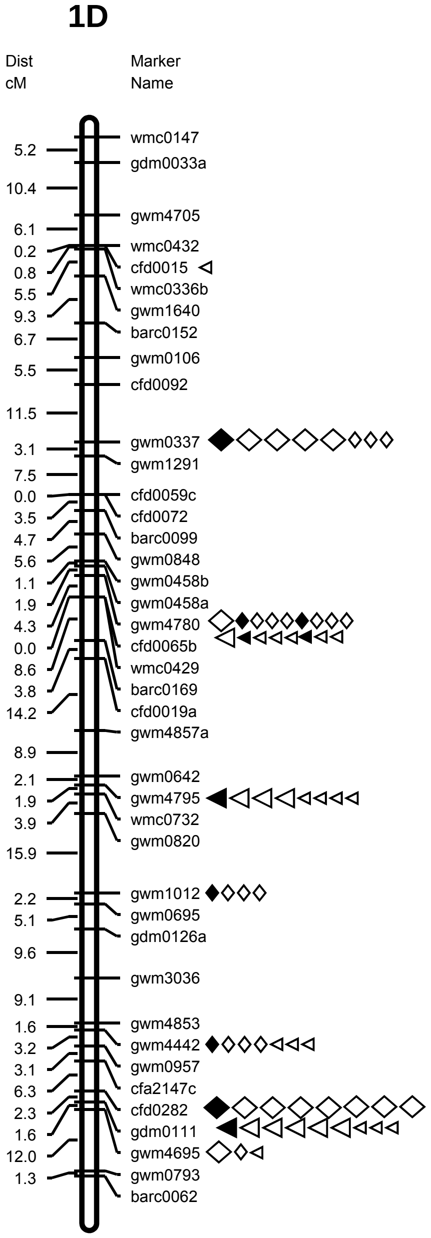
1D
Dist
cM
Marker
Name
5.2
10.4
6.1
0.2
0.8
5.5
9.3
6.7
5.5
11.5
3.1
7.5
0.0
3.5
4.7
5.6
1.1
1.9
4.3
0.0
8.6
3.8
14.2
8.9
2.1
1.9
3.9
15.9
2.2
5.1
9.6
9.1
1.6
3.2
3.1
6.3
2.3
1.6
12.0
1.3
wmc0147
gdm0033a
gwm4705
wmc0432
cfd0015
wmc0336b
gwm1640
barc0152
gwm0106
cfd0092
gwm0337
gwm1291
cfd0059c
cfd0072
barc0099
gwm0848
gwm0458b
gwm0458a
gwm4780
cfd0065b
wmc0429
barc0169
cfd0019a
gwm4857a
gwm0642
gwm4795
wmc0732
gwm0820
gwm1012
gwm0695
gdm0126a
gwm3036
gwm4853
gwm4442
gwm0957
cfa2147c
cfd0282
gdm0111
gwm4695
gwm0793
barc0062

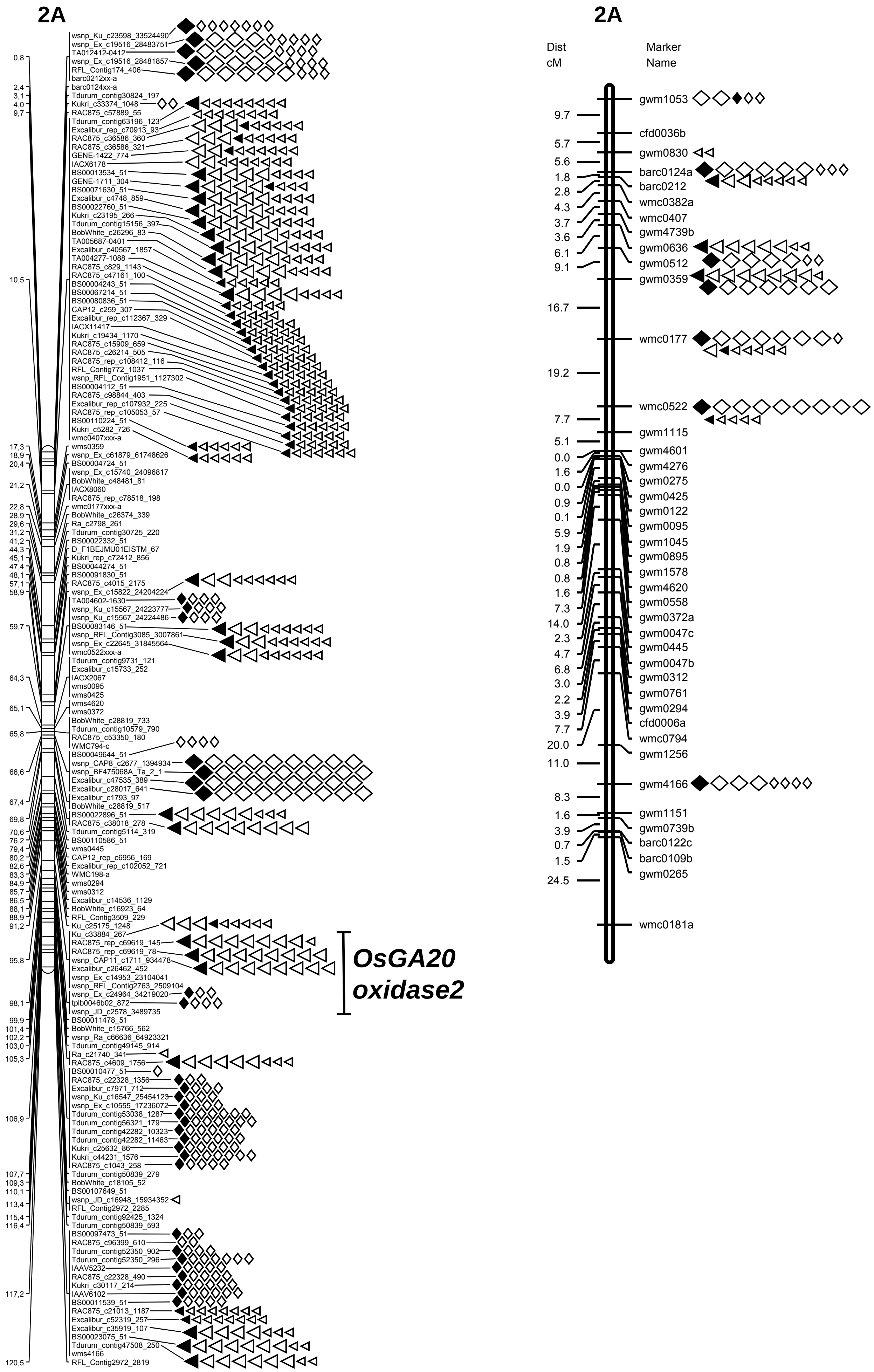
2A
wsnp_Ku_c23598_33524490
wsnp_Ex_c19516_28483751
TA012412-0412
wsnp_Ex_c19516_28481857
RFL_Contig174_406
barc0212xx-a
barc0124xx-a
Tdurum_contig30824_197
Kukri_c33374_1048
RAC875_c57889_55
Tdurum_contig63196_123
Excalibur_rep_c70913_93
RAC875_c36586_360
RAC875_c36586_321
GENE-1422_774
IACX6178
BS00013534_51
GENE-1711_304
BS00071630_51
Excalibur_c4748_859
BS00022760_51
Kukri_c23195_266
Tdurum_contig15156_397
BobWhite_c26296_83
TA005687-0401
Excalibur_c40567_1857
TA004277-1088
RAC875_c829_1143
RAC875_c47161_100
BS00004243_51
BS00067214_51
BS00080836_51
CAP12_c259_307
Excalibur_rep_c112367_329
IACX11417
Kukri_c19434_1170
RAC875_c15909_659
RAC875_c26214_505
RAC875_rep_c108412_116
RFL_Contig772_1037
wsnp_RFL_Contig1951_1127302
BS00004112_51
RAC875_c98844_403
Excalibur_rep_c107932_225
RAC875_rep_c105053_57
BS00110224_51
Kukri_c5282_726
wmc0407xxx-a
wms0359
wsnp_Ex_c61879_61748626
BS00004724_51
wsnp_Ex_c15740_24096817
BobWhite_c48481_81
IACX8060
RAC875_rep_c78518_198
wmc0177xxx-a
BobWhite_c26374_339
Ra_c2798_261
Tdurum_contig30725_220
BS00022332_51
D_F1BEJMU01EISTM_67
Kukri_rep_c72412_856
BS00044274_51
BS00091830_51
RAC875_c4015_2175
wsnp_Ex_c15822_24204224
TA004602-1630
wsnp_Ku_c15567_24223777
wsnp_Ku_c15567_24224486
BS00083146_51
wsnp_RFL_Contig3085_3007861
wsnp_Ex_c22645_31845564
wmc0522xxx-a
Tdurum_contig9731_121
Excalibur_c15733_252
IACX2067
wms0095
wms0425
wms4620
wms0372
BobWhite_c28819_733
Tdurum_contig10579_790
RAC875_c53350_180
WMC794-c
BS00049644_51
wsnp_CAP8_c2677_1394934
wsnp_BF475068A_Ta_2_1
Excalibur_c47535_389
Excalibur_c28017_641
Excalibur_c1793_97
BobWhite_c28819_517
BS00022896_51
RAC875_c38018_278
Tdurum_contig5114_319
BS00110586_51
wms0445
CAP12_rep_c6956_169
Excalibur_rep_c102052_721
WMC198-a
wms0294
wms0312
Excalibur_c14536_1129
BobWhite_c16923_64
RFL_Contig3509_229
Ku_c25175_1248
Ku_c33884_267
RAC875_rep_c69619_145
RAC875_rep_c69619_78
wsnp_CAP11_c1711_934478
Excalibur_c26462_452
wsnp_Ex_c14953_23104041
wsnp_RFL_Contig2763_2509104
wsnp_Ex_c24964_34219020
tplb0046b02_872
wsnp_JD_c2578_3489735
BS00011478_51
BobWhite_c15766_562
wsnp_Ra_c66636_64923321
Tdurum_contig49145_914
Ra_c21740_341
RAC875_c4609_1756
BS00010477_51
RAC875_c22328_1356
Excalibur_c7971_712
wsnp_Ku_c16547_25454123
wsnp_Ex_c10555_17236072
Tdurum_contig53038_1287
Tdurum_contig56321_179
Tdurum_contig42282_10323
Tdurum_contig42282_11463
Kukri_c25632_86
Kukri_c44231_1576
RAC875_c1043_258
Tdurum_contig50839_279
BobWhite_c18105_52
BS00107649_51
wsnp_JD_c16948_15934352
RFL_Contig2972_2285
Tdurum_contig92425_1324
Tdurum_contig50839_593
BS00097473_51
RAC875_c96399_610
Tdurum_contig52350_902
Tdurum_contig52350_296
IAAV5232
RAC875_c22328_490
Kukri_c30117_214
IAAV6102
BS00011539_51
RAC875_c21013_1187
Excalibur_c52319_257
Excalibur_c35919_107
BS00023075_51
Tdurum_contig47508_250
wms4166
RFL_Contig2972_2819
0,8
2,4
3,1
4,0
9,7
10,5
17,3
18,9
20,4
21,2
22,8
28,9
29,6
31,2
41,2
44,3
45,1
47,4
48,1
57,1
58,9
59,7
64,3
65,1
65,8
66,6
67,4
69,8
70,6
76,2
79,4
80,2
82,6
83,3
84,9
85,7
86,5
88,1
88,9
91,2
95,8
98,1
99,9
101,4
102,2
103,0
105,3
106,9
107,7
109,3
110,1
113,4
115,4
116,4
117,2
120,5
OsGA20
oxidase2
2A
Dist
cM
Marker
Name
gwm1053
9.7
cfd0036b
5.7
gwm0830
5.6
barc0124a
1.8
barc0212
2.8
wmc0382a
4.3
wmc0407
3.7
gwm4739b
3.6
gwm0636
6.1
gwm0512
9.1
gwm0359
16.7
wmc0177
19.2
wmc0522
7.7
gwm1115
5.1
gwm4601
0.0
gwm4276
1.6
gwm0275
0.0
gwm0425
0.9
gwm0122
0.1
gwm0095
5.9
gwm1045
1.9
gwm0895
0.8
gwm1578
0.8
gwm4620
1.6
gwm0558
7.3
gwm0372a
14.0
gwm0047c
2.3
gwm0445
4.7
gwm0047b
6.8
gwm0312
3.0
gwm0761
2.2
gwm0294
3.9
cfd0006a
7.7
wmc0794
20.0
gwm1256
11.0
gwm4166
8.3
gwm1151
1.6
gwm0739b
3.9
barc0122c
0.7
barc0109b
1.5
gwm0265
24.5
wmc0181a

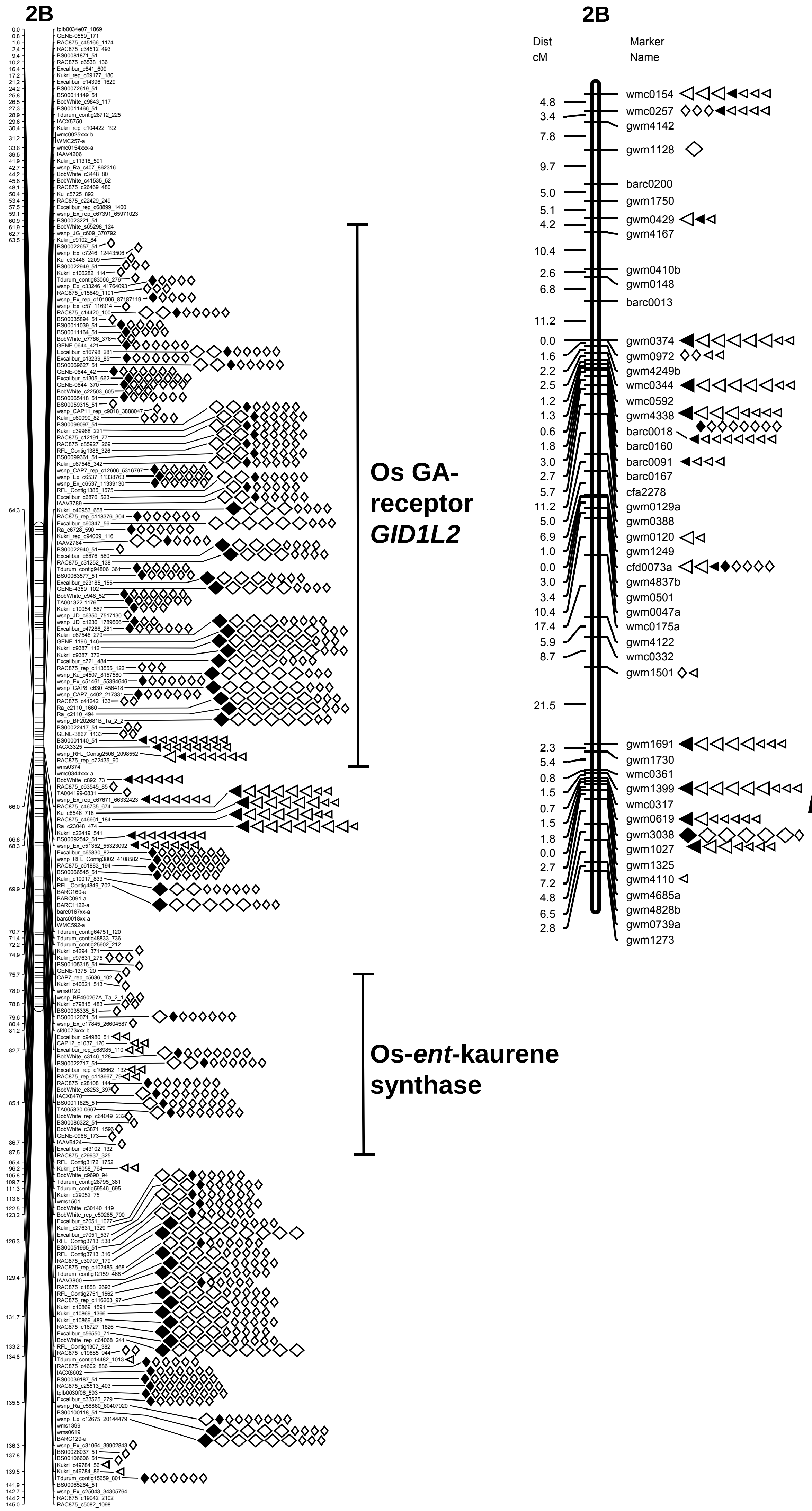

2B
Os GA-receptor GID1L2
Os-ent-kaurene synthase
2B
Dist cM
Marker Name
4.8
3.4
7.8
9.7
5.0
5.1
4.2
10.4
2.6
6.8
11.2
0.0
1.6
2.2
2.5
1.2
1.3
0.6
1.8
3.0
2.7
5.7
11.2
5.0
6.9
1.0
0.0
3.0
3.4
10.4
17.4
5.9
8.7
21.5
2.3
5.4
0.8
1.5
0.7
1.5
1.8
0.0
2.7
7.2
4.8
6.5
2.8
wmc0154
wmc0257
gwm4142
gwm1128
barc0200
gwm1750
gwm0429
gwm4167
gwm0410b
gwm0148
barc0013
gwm0374
gwm0972
gwm4249b
wmc0344
wmc0592
gwm4338
barc0018
barc0160
barc0091
barc0167
cfa2278
gwm0129a
gwm0388
gwm0120
gwm1249
cfd0073a
gwm4837b
gwm0501
gwm0047a
wmc0175a
gwm4122
wmc0332
gwm1501
gwm1691
gwm1730
wmc0361
gwm1399
wmc0317
gwm0619
gwm3038
gwm1027
gwm1325
gwm4110
gwm4685a
gwm4828b
gwm0739a
gwm1273
Rht4

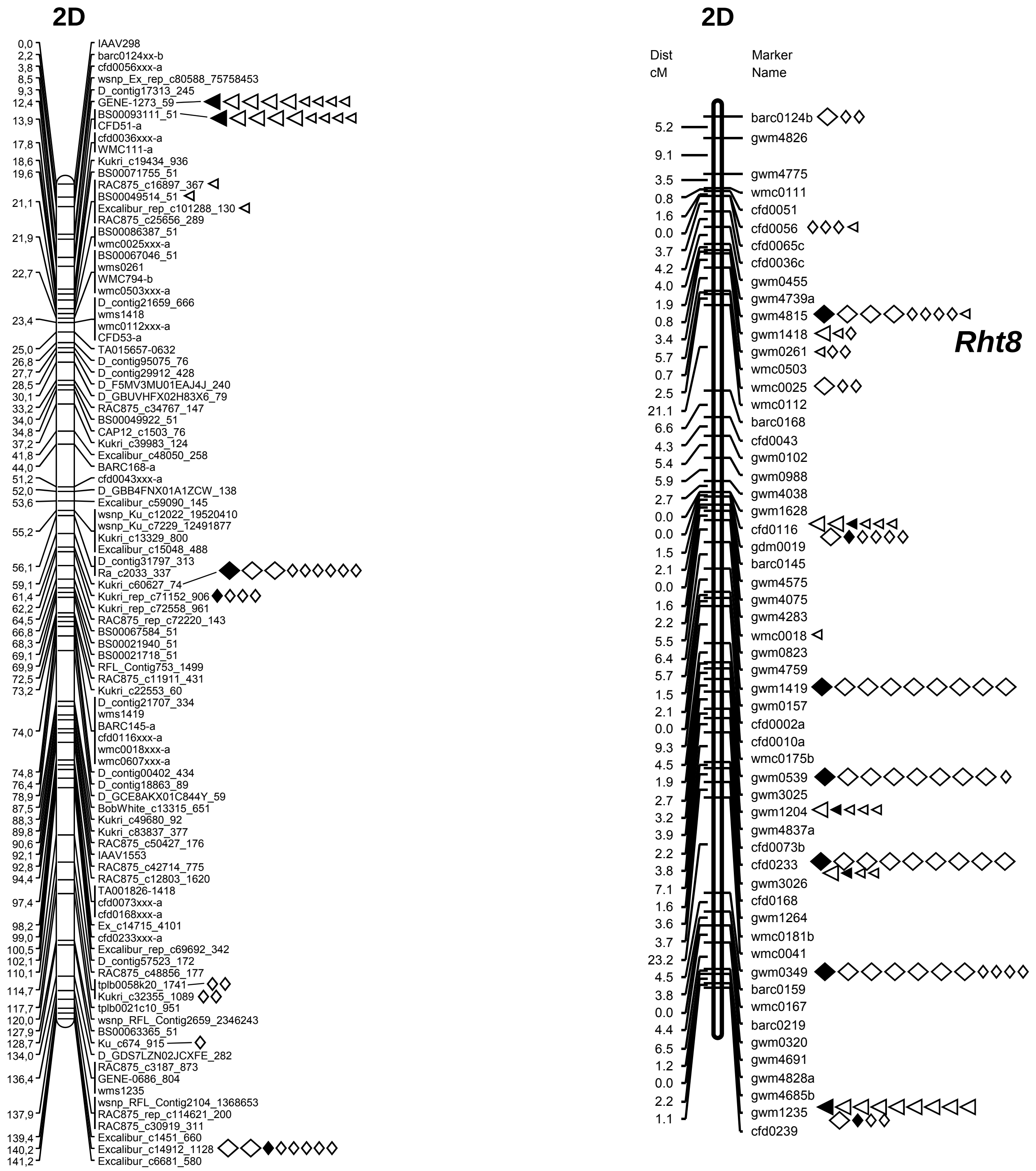

2D
0,0 IAAV298
2,2 barc0124xx-b
3,8 cfd0056xxx-a
8,5 wsnp_Ex_rep_c80588_75758453
9,3 D_contig17313_245
12,4 GENE-1273_59
13,9 BS00093111_51
CFD51-a
17,8 cfd0036xxx-a
WMC111-a
18,6 Kukri_c19434_936
19,6 BS00071755_51
RAC875_c16897_367
BS00049514_51
21,1 Excalibur_rep_c101288_130
RAC875_c25656_289
21,9 BS00086387_51
wmc0025xxx-a
BS00067046_51
22,7 wms0261
WMC794-b
wmc0503xxx-a
D_contig21659_666
23,4 wms1418
wmc0112xxx-a
CFD53-a
25,0 TA015657-0632
26,8 D_contig95075_76
27,7 D_contig29912_428
28,5 D_F5MV3MU01EAJ4J_240
30,1 D_GBUVHFX02H83X6_79
33,2 RAC875_c34767_147
34,0 BS00049922_51
34,8 CAP12_c1503_76
37,2 Kukri_c39983_124
41,8 Excalibur_c48050_258
44,0 BARC168-a
51,2 cfd0043xxx-a
52,0 D_GBB4FNX01A1ZCW_138
53,6 Excalibur_c59090_145
wsnp_Ku_c12022_19520410
55,2 wsnp_Ku_c7229_12491877
Kukri_c13329_800
Excalibur_c15048_488
56,1 D_contig31797_313
Ra_c2033_337
59,1 Kukri_c60627_74
61,4 Kukri_rep_c71152_906
62,2 Kukri_rep_c72558_961
64,5 RAC875_rep_c72220_143
66,8 BS00067584_51
68,3 BS00021940_51
69,1 BS00021718_51
69,9 RFL_Contig753_1499
72,5 RAC875_c11911_431
73,2 Kukri_c22553_60
D_contig21707_334
wms1419
74,0 BARC145-a
cfd0116xxx-a
wmc0018xxx-a
wmc0607xxx-a
74,8 D_contig00402_434
76,4 D_contig18863_89
78,9 D_GCE8AKX01C844Y_59
87,5 BobWhite_c13315_651
88,3 Kukri_c49680_92
89,8 Kukri_c83837_377
90,6 RAC875_c50427_176
92,1 IAAV1553
92,8 RAC875_c42714_775
94,4 RAC875_c12803_1620
TA001826-1418
97,4 cfd0073xxx-a
cfd0168xxx-a
98,2 Ex_c14715_4101
99,0 cfd0233xxx-a
100,5 Excalibur_rep_c69692_342
102,1 D_contig57523_172
110,1 RAC875_c48856_177
114,7 tplb0058k20_1741
Kukri_c32355_1089
117,7 tplb0021c10_951
120,0 wsnp_RFL_Contig2659_2346243
127,9 BS00063365_51
128,7 Ku_c674_915
134,0 D_GDS7LZN02JCXFE_282
RAC875_c3187_873
136,4 GENE-0686_804
wms1235
wsnp_RFL_Contig2104_1368653
137,9 RAC875_rep_c114621_200
RAC875_c30919_311
139,4 Excalibur_c1451_660
140,2 Excalibur_c14912_1128
141,2 Excalibur_c6681_580
2D
Dist cM
Marker Name
barc0124b
5.2 gwm4826
9.1
3.5 gwm4775
0.8 wmc0111
1.6 cfd0051
0.0 cfd0056
3.7 cfd0065c
4.2 cfd0036c
4.0 gwm0455
1.9 gwm4739a
0.8 gwm4815
3.4 gwm1418
5.7 gwm0261
0.7 wmc0503
2.5 wmc0025
21.1 wmc0112
6.6 barc0168
4.3 cfd0043
5.4 gwm0102
5.9 gwm0988
2.7 gwm4038
0.0 gwm1628
0.0 cfd0116
1.5 gdm0019
2.1 barc0145
0.0 gwm4575
1.6 gwm4075
2.2 gwm4283
5.5 wmc0018
6.4 gwm0823
5.7 gwm4759
1.5 gwm1419
2.1 gwm0157
0.0 cfd0002a
9.3 cfd0010a
4.5 wmc0175b
1.9 gwm0539
2.7 gwm3025
3.2 gwm1204
3.9 gwm4837a
2.2 cfd0073b
3.8 cfd0233
7.1 gwm3026
1.6 cfd0168
3.6 gwm1264
3.7 wmc0181b
23.2 wmc0041
4.5 gwm0349
3.8 barc0159
0.0 wmc0167
4.4 barc0219
6.5 gwm0320
1.2 gwm4691
0.0 gwm4828a
2.2 gwm4685b
1.1 gwm1235
cfd0239
Rht8

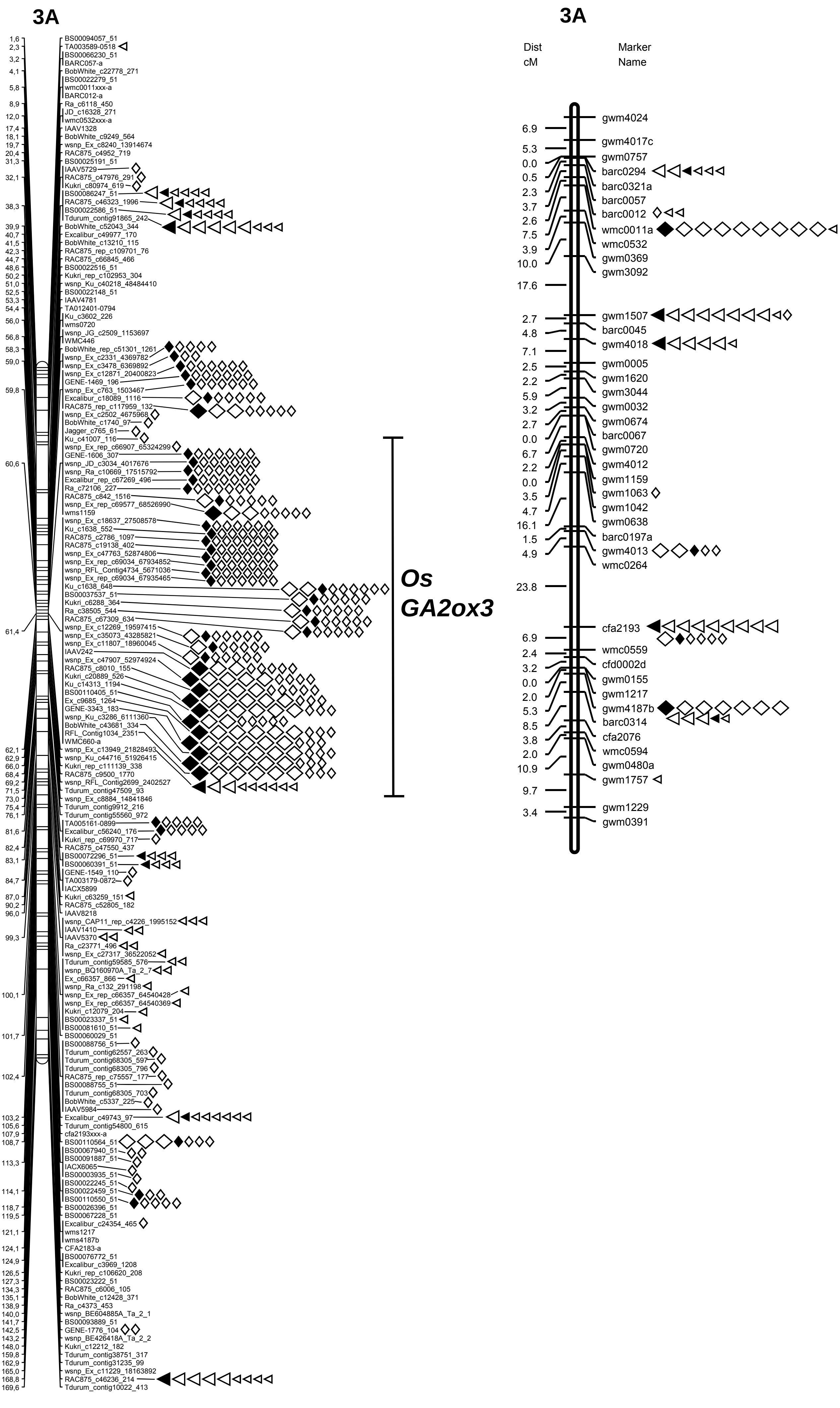
3A
BS00094057_51
TA003589-0518
BS00066230_51
BARC057-a
BobWhite_c22778_271
BS00022279_51
wmc0011xxx-a
BARC012-a
Ra_c6118_450
JD_c16328_271
wmc0532xxx-a
IAAV1328
BobWhite_c9249_564
wsnp_Ex_c8240_13914674
RAC875_c4952_719
BS00025191_51
IAAV5729
RAC875_c47976_291
Kukri_c80974_619
BS00086247_51
RAC875_c46323_1996
BS00022586_51
Tdurum_contig91865_242
BobWhite_c52043_344
Excalibur_c49977_170
BobWhite_c13210_115
RAC875_rep_c109701_76
RAC875_c66845_466
BS00022516_51
Kukri_rep_c102953_304
wsnp_Ku_c40218_48484410
BS00022148_51
IAAV4781
TA012401-0794
Ku_c3602_226
wms0720
wsnp_JG_c2509_1153697
WMC446
BobWhite_rep_c51301_1261
wsnp_Ex_c2331_4369782
wsnp_Ex_c3478_6369892
wsnp_Ex_c12871_20400823
GENE-1469_196
wsnp_Ex_c763_1503467
Excalibur_c18089_1116
RAC875_rep_c117959_132
wsnp_Ex_c2502_4675968
BobWhite_c1740_97
Jagger_c765_61
Ku_c41007_116
wsnp_Ex_rep_c66907_65324299
GENE-1606_307
wsnp_JD_c3034_4017676
wsnp_Ra_c10669_17515792
Excalibur_rep_c67269_496
Ra_c72106_227
RAC875_c842_1516
wsnp_Ex_rep_c69577_68526990
wms1159
wsnp_Ex_c18637_27508578
Ku_c1638_552
RAC875_c2786_1097
RAC875_c19138_402
wsnp_Ex_c47763_52874806
wsnp_Ex_rep_c69034_67934852
wsnp_RFL_Contig4734_5671036
wsnp_Ex_rep_c69034_67935465
Ku_c1638_648
BS00037537_51
Kukri_c6288_364
Ra_c38505_544
RAC875_c67309_634
wsnp_Ex_c12269_19597415
wsnp_Ex_c35073_43285821
wsnp_Ex_c11807_18960045
IAAV242
wsnp_Ex_c47907_52974924
RAC875_c8010_155
Kukri_c20889_526
Ku_c14313_1194
BS00110405_51
Ex_c9685_1264
GENE-3343_183
wsnp_Ku_c3286_6111360
BobWhite_c43681_334
RFL_Contig1034_2351
WMC660-a
wsnp_Ex_c13949_21828493
wsnp_Ku_c44716_51926415
Kukri_rep_c111139_338
RAC875_c9500_1770
wsnp_RFL_Contig2699_2402527
Tdurum_contig47509_93
wsnp_Ex_c8884_14841846
Tdurum_contig9912_216
Tdurum_contig55560_972
TA005161-0899
Excalibur_c56240_176
Kukri_rep_c69970_717
RAC875_c47550_437
BS00072296_51
BS00060391_51
GENE-1549_110
TA003179-0872
IACX5899
Kukri_c63259_151
RAC875_c52805_182
IAAV8218
wsnp_CAP11_rep_c4226_1995152
IAAV1410
IAAV5370
Ra_c23771_496
wsnp_Ex_c27317_36522052
Tdurum_contig59585_576
wsnp_BQ160970A_Ta_2_7
Ex_c66357_866
wsnp_Ra_c132_291198
wsnp_Ex_rep_c66357_64540428
wsnp_Ex_rep_c66357_64540369
Kukri_c12079_204
BS00023337_51
BS00081610_51
BS00060029_51
BS00088756_51
Tdurum_contig62557_263
Tdurum_contig68305_597
Tdurum_contig68305_796
RAC875_rep_c75557_177
BS00088755_51
Tdurum_contig68305_703
BobWhite_c5337_225
IAAV5984
Excalibur_c49743_97
Tdurum_contig54800_615
cfa2193xxx-a
BS00110564_51
BS00067940_51
BS00091887_51
IACX6065
BS00003935_51
BS00022245_51
BS00022459_51
BS00110550_51
BS00026396_51
BS00067228_51
Excalibur_c24354_465
wms1217
wms4187b
CFA2183-a
BS00076772_51
Excalibur_c3969_1208
Kukri_rep_c106620_208
BS00023222_51
RAC875_c6006_105
BobWhite_c12428_371
Ra_c4373_453
wsnp_BE604885A_Ta_2_1
BS00093889_51
GENE-1776_104
wsnp_BE426418A_Ta_2_2
Kukri_c12212_182
Tdurum_contig38751_317
Tdurum_contig31235_99
wsnp_Ex_c11229_18163892
RAC875_c46236_214
Tdurum_contig10022_413
Os
GA2ox3
3A
Dist
cM
Marker
Name
gwm4024
6.9
gwm4017c
5.3
gwm0757
0.0
barc0294
0.5
barc0321a
2.3
barc0057
3.7
barc0012
2.6
wmc0011a
7.5
wmc0532
3.9
gwm0369
10.0
gwm3092
17.6
2.7
gwm1507
4.8
barc0045
gwm4018
7.1
2.5
gwm0005
2.2
gwm1620
5.9
gwm3044
3.2
gwm0032
2.7
gwm0674
0.0
barc0067
6.7
gwm0720
2.2
gwm4012
0.0
gwm1159
3.5
gwm1063
4.7
gwm1042
16.1
gwm0638
1.5
barc0197a
4.9
gwm4013
wmc0264
23.8
cfa2193
6.9
2.4
wmc0559
3.2
cfd0002d
0.0
gwm0155
2.0
gwm1217
5.3
gwm4187b
8.5
barc0314
3.8
cfa2076
2.0
wmc0594
10.9
gwm0480a
9.7
gwm1757
3.4
gwm1229
gwm0391

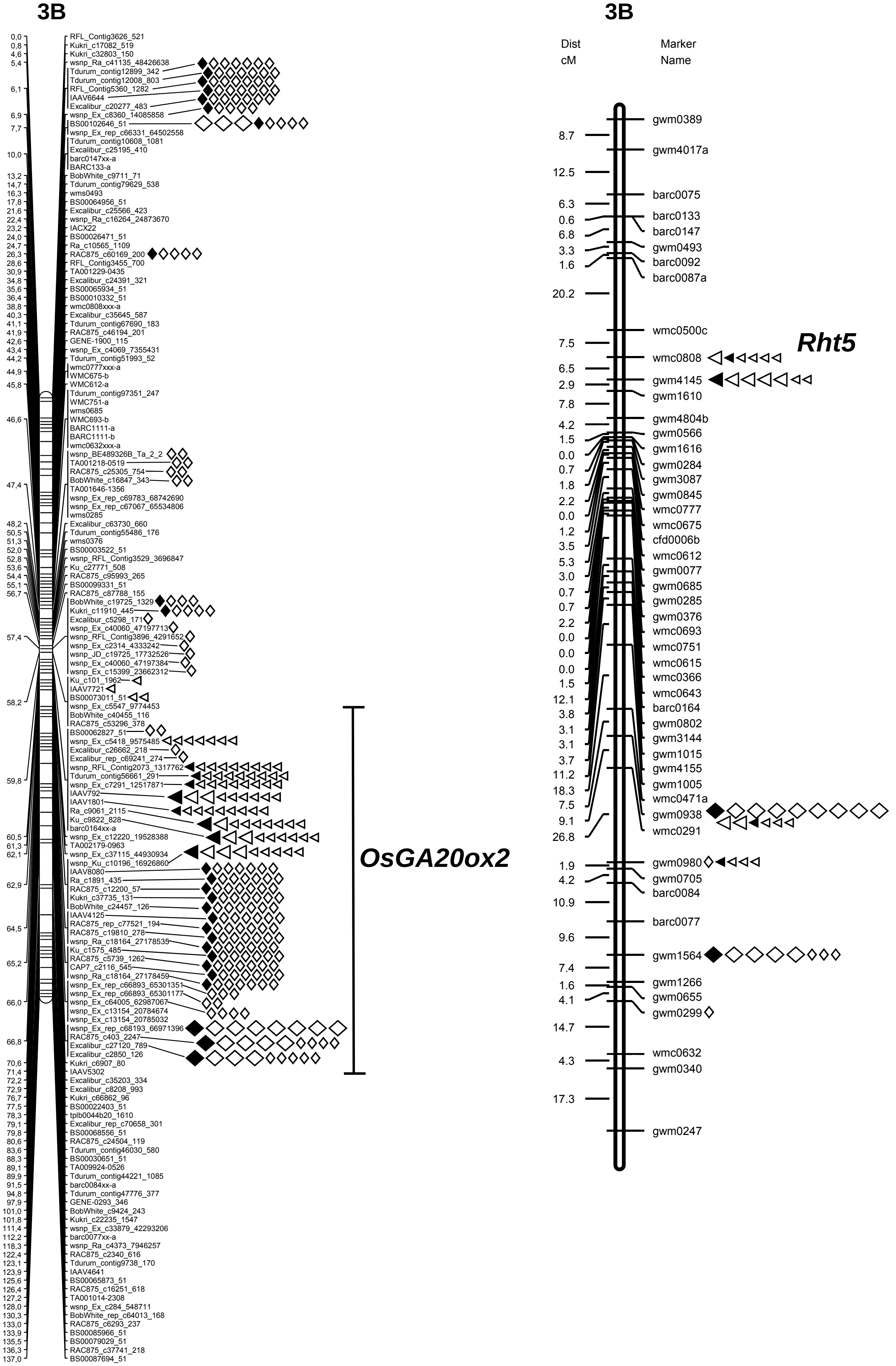
3B
OsGA20ox2
3B
Dist
cM
Marker
Name
Rht5
gwm0389
gwm4017a
barc0075
barc0133
barc0147
gwm0493
barc0092
barc0087a
wmc0500c
wmc0808
gwm4145
gwm1610
gwm4804b
gwm0566
gwm1616
gwm0284
gwm3087
gwm0845
wmc0777
wmc0675
cfd0006b
wmc0612
gwm0077
gwm0685
gwm0285
gwm0376
wmc0693
wmc0751
wmc0615
wmc0366
wmc0643
barc0164
gwm0802
gwm3144
gwm1015
gwm4155
gwm1005
wmc0471a
gwm0938
wmc0291
gwm0980
gwm0705
barc0084
barc0077
gwm1564
gwm1266
gwm0655
gwm0299
wmc0632
gwm0340
gwm0247

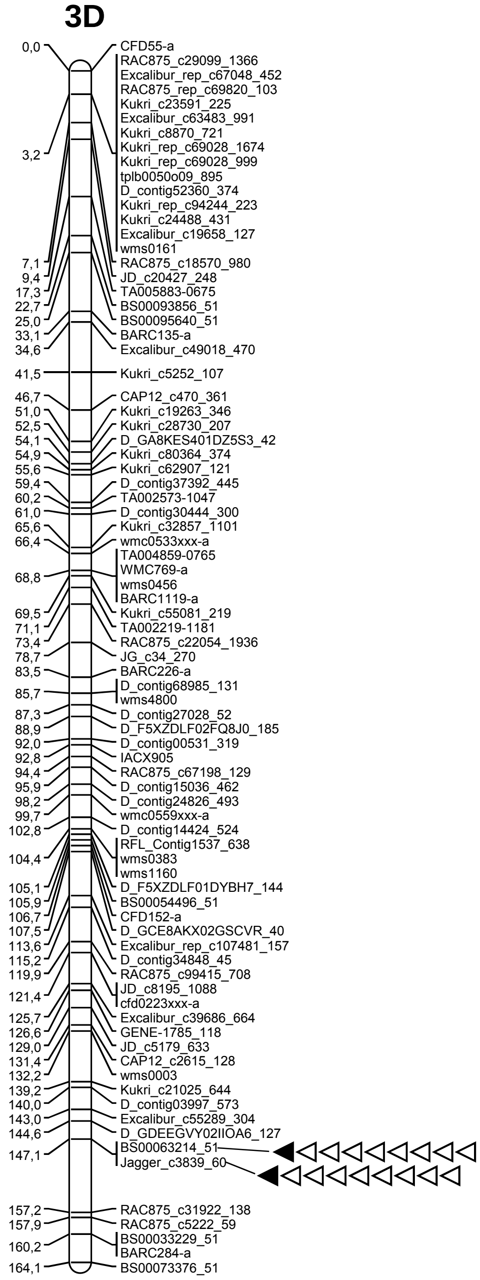

3D
0,0 CFD55-a
RAC875_c29099_1366
Excalibur_rep_c67048_452
RAC875_rep_c69820_103
Kukri_c23591_225
Excalibur_c63483_991
Kukri_c8870_721
Kukri_rep_c69028_1674
3,2 Kukri_rep_c69028_999
tplb0050o09_895
D_contig52360_374
Kukri_rep_c94244_223
Kukri_c24488_431
Excalibur_c19658_127
wms0161
7,1 RAC875_c18570_980
9,4 JD_c20427_248
17,3 TA005883-0675
22,7 BS00093856_51
25,0 BS00095640_51
33,1 BARC135-a
34,6 Excalibur_c49018_470
41,5 Kukri_c5252_107
46,7 CAP12_c470_361
51,0 Kukri_c19263_346
52,5 Kukri_c28730_207
54,1 D_GA8KES401DZ5S3_42
54,9 Kukri_c80364_374
55,6 Kukri_c62907_121
59,4 D_contig37392_445
60,2 TA002573-1047
61,0 D_contig30444_300
65,6 Kukri_c32857_1101
66,4 wmc0533xxx-a
TA004859-0765
68,8 WMC769-a
wms0456
BARC1119-a
69,5 Kukri_c55081_219
71,1 TA002219-1181
73,4 RAC875_c22054_1936
78,7 JG_c34_270
83,5 BARC226-a
85,7 D_contig68985_131
wms4800
87,3 D_contig27028_52
88,9 D_F5XZDLF02FQ8J0_185
92,0 D_contig00531_319
92,8 IACX905
94,4 RAC875_c67198_129
95,9 D_contig15036_462
98,2 D_contig24826_493
99,7 wmc0559xxx-a
102,8 D_contig14424_524
RFL_Contig1537_638
104,4 wms0383
wms1160
105,1 D_F5XZDLF01DYBH7_144
105,9 BS00054496_51
106,7 CFD152-a
107,5 D_GCE8AKX02GSCVR_40
113,6 Excalibur_rep_c107481_157
115,2 D_contig34848_45
119,9 RAC875_c99415_708
121,4 JD_c8195_1088
cfd0223xxx-a
125,7 Excalibur_c39686_664
126,6 GENE-1785_118
129,0 JD_c5179_633
131,4 CAP12_c2615_128
132,2 wms0003
139,2 Kukri_c21025_644
140,0 D_contig03997_573
143,0 Excalibur_c55289_304
144,6 D_GDEEGVY02IIOA6_127
147,1 BS00063214_51
Jagger_c3839_60
157,2 RAC875_c31922_138
157,9 RAC875_c5222_59
160,2 BS00033229_51
BARC284-a
164,1 BS00073376_51

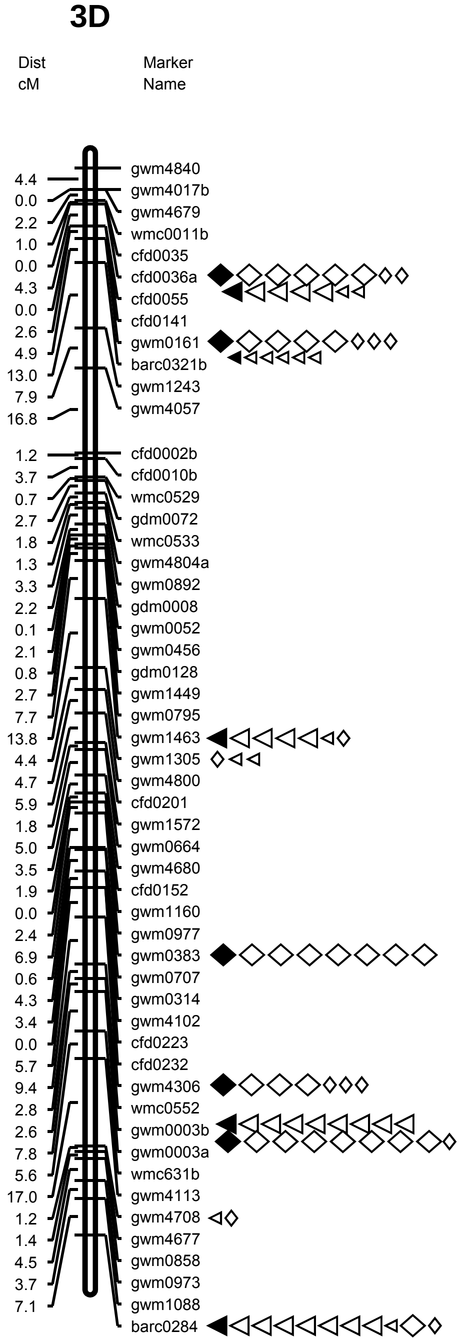

3D
Dist cM
Marker Name
4.4 gwm4840
0.0 gwm4017b
2.2 gwm4679
1.0 wmc0011b
0.0 cfd0035
4.3 cfd0036a
0.0 cfd0055
2.6 cfd0141
4.9 gwm0161
13.0 barc0321b
7.9 gwm1243
16.8 gwm4057
1.2 cfd0002b
3.7 cfd0010b
0.7 wmc0529
2.7 gdm0072
1.8 wmc0533
1.3 gwm4804a
3.3 gwm0892
2.2 gdm0008
0.1 gwm0052
2.1 gwm0456
0.8 gdm0128
2.7 gwm1449
7.7 gwm0795
13.8 gwm1463
4.4 gwm1305
4.7 gwm4800
5.9 cfd0201
1.8 gwm1572
5.0 gwm0664
3.5 gwm4680
1.9 cfd0152
0.0 gwm1160
2.4 gwm0977
6.9 gwm0383
0.6 gwm0707
4.3 gwm0314
3.4 gwm4102
0.0 cfd0223
5.7 cfd0232
9.4 gwm4306
2.8 wmc0552
2.6 gwm0003b
7.8 gwm0003a
5.6 wmc631b
17.0 gwm4113
1.2 gwm4708
1.4 gwm4677
4.5 gwm0858
3.7 gwm0973
7.1 gwm1088
barc0284

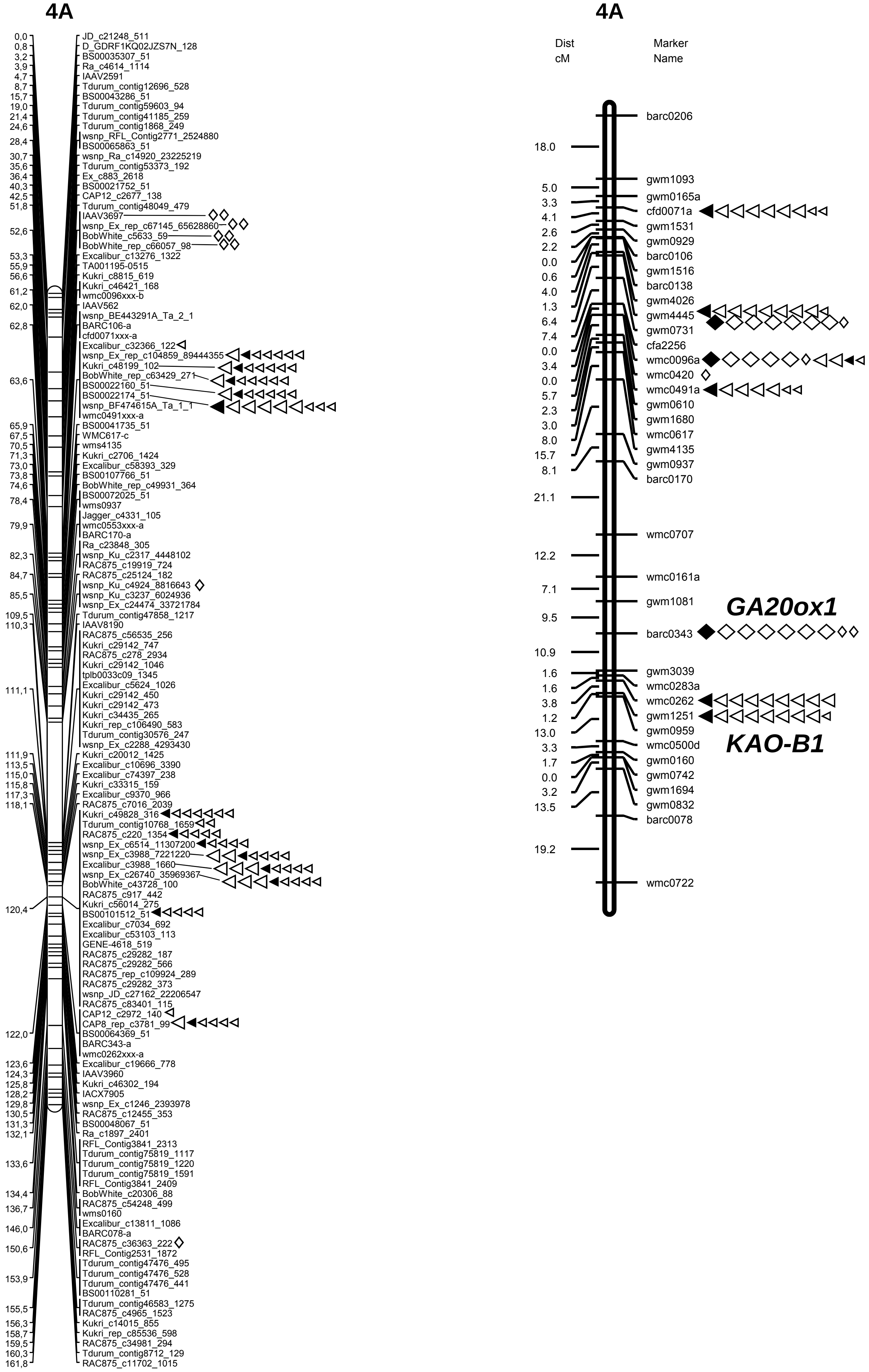

4A
0,0
0,8
3,2
3,9
4,7
8,7
15,7
19,0
21,4
24,6
28,4
30,7
35,6
36,4
40,3
42,5
51,8
52,6
53,3
55,9
56,6
61,2
62,0
62,8
63,6
65,9
67,5
70,5
71,3
73,0
73,8
74,6
78,4
79,9
82,3
84,7
85,5
109,5
110,3
111,1
111,9
113,5
115,0
115,8
117,3
118,1
120,4
122,0
123,6
124,3
125,8
128,2
129,8
130,5
131,3
132,1
133,6
134,4
136,7
146,0
150,6
153,9
155,5
156,3
158,7
159,5
160,3
161,8
JD_c21248_511
D_GDRF1KQ02JZS7N_128
BS00035307_51
Ra_c4614_1114
IAAV2591
Tdurum_contig12696_528
BS00043286_51
Tdurum_contig59603_94
Tdurum_contig41185_259
Tdurum_contig1868_249
wsnp_RFL_Contig2771_2524880
BS00065863_51
wsnp_Ra_c14920_23225219
Tdurum_contig53373_192
Ex_c883_2618
BS00021752_51
CAP12_c2677_138
Tdurum_contig48049_479
IAAV3697
wsnp_Ex_rep_c67145_65628860
BobWhite_c5633_59
BobWhite_rep_c66057_98
Excalibur_c13276_1322
TA001195-0515
Kukri_c8815_619
Kukri_c46421_168
wmc0096xxx-b
IAAV562
wsnp_BE443291A_Ta_2_1
BARC106-a
cfd0071xxx-a
Excalibur_c32366_122
wsnp_Ex_rep_c104859_89444355
Kukri_c48199_102
BobWhite_rep_c63429_271
BS00022160_51
BS00022174_51
wsnp_BF474615A_Ta_1_1
wmc0491xxx-a
BS00041735_51
WMC617-c
wms4135
Kukri_c2706_1424
Excalibur_c58393_329
BS00107766_51
BobWhite_rep_c49931_364
BS00072025_51
wms0937
Jagger_c4331_105
wmc0553xxx-a
BARC170-a
Ra_c23848_305
wsnp_Ku_c2317_4448102
RAC875_c19919_724
RAC875_c25124_182
wsnp_Ku_c4924_8816643
wsnp_Ku_c3237_6024936
wsnp_Ex_c24474_33721784
Tdurum_contig47858_1217
IAAV8190
RAC875_c56535_256
Kukri_c29142_747
RAC875_c278_2934
Kukri_c29142_1046
tplb0033c09_1345
Excalibur_c5624_1026
Kukri_c29142_450
Kukri_c29142_473
Kukri_c34435_265
Kukri_rep_c106490_583
Tdurum_contig30576_247
wsnp_Ex_c2288_4293430
Kukri_c20012_1425
Excalibur_c10696_3390
Excalibur_c74397_238
Kukri_c33315_159
Excalibur_c9370_966
RAC875_c7016_2039
Kukri_c49828_316
Tdurum_contig10768_1659
RAC875_c220_1354
wsnp_Ex_c6514_11307200
wsnp_Ex_c3988_7221220
Excalibur_c3988_1660
wsnp_Ex_c26740_35969367
BobWhite_c43728_100
RAC875_c917_442
Kukri_c56014_275
BS00101512_51
Excalibur_c7034_692
Excalibur_c53103_113
GENE-4618_519
RAC875_c29282_187
RAC875_c29282_566
RAC875_rep_c109924_289
RAC875_c29282_373
wsnp_JD_c27162_22206547
RAC875_c83401_115
CAP12_c2972_140
CAP8_rep_c3781_99
BS00064369_51
BARC343-a
wmc0262xxx-a
Excalibur_c19666_778
IAAV3960
Kukri_c46302_194
IACX7905
wsnp_Ex_c1246_2393978
RAC875_c12455_353
BS00048067_51
Ra_c1897_2401
RFL_Contig3841_2313
Tdurum_contig75819_1117
Tdurum_contig75819_1220
Tdurum_contig75819_1591
RFL_Contig3841_2409
BobWhite_c20306_88
RAC875_c54248_499
wms0160
Excalibur_c13811_1086
BARC078-a
RAC875_c36363_222
RFL_Contig2531_1872
Tdurum_contig47476_495
Tdurum_contig47476_528
Tdurum_contig47476_441
BS00110281_51
Tdurum_contig46583_1275
RAC875_c4965_1523
Kukri_c14015_855
Kukri_rep_c85536_598
RAC875_c34981_294
Tdurum_contig8712_129
RAC875_c11702_1015
4A
Dist
cM
Marker
Name
barc0206
18.0
5.0
gwm1093
3.3
gwm0165a
4.1
cfd0071a
2.6
gwm1531
2.2
gwm0929
0.0
barc0106
0.6
gwm1516
4.0
barc0138
1.3
gwm4026
6.4
gwm4445
7.4
gwm0731
0.0
cfa2256
3.4
wmc0096a
0.0
wmc0420
5.7
wmc0491a
2.3
gwm0610
3.0
gwm1680
8.0
wmc0617
15.7
gwm4135
8.1
gwm0937
barc0170
21.1
wmc0707
12.2
wmc0161a
7.1
gwm1081
9.5
GA20ox1
barc0343
10.9
1.6
gwm3039
1.6
wmc0283a
3.8
wmc0262
1.2
gwm1251
13.0
gwm0959
3.3
wmc0500d
KAO-B1
1.7
gwm0160
0.0
gwm0742
3.2
gwm1694
13.5
gwm0832
barc0078
19.2
wmc0722

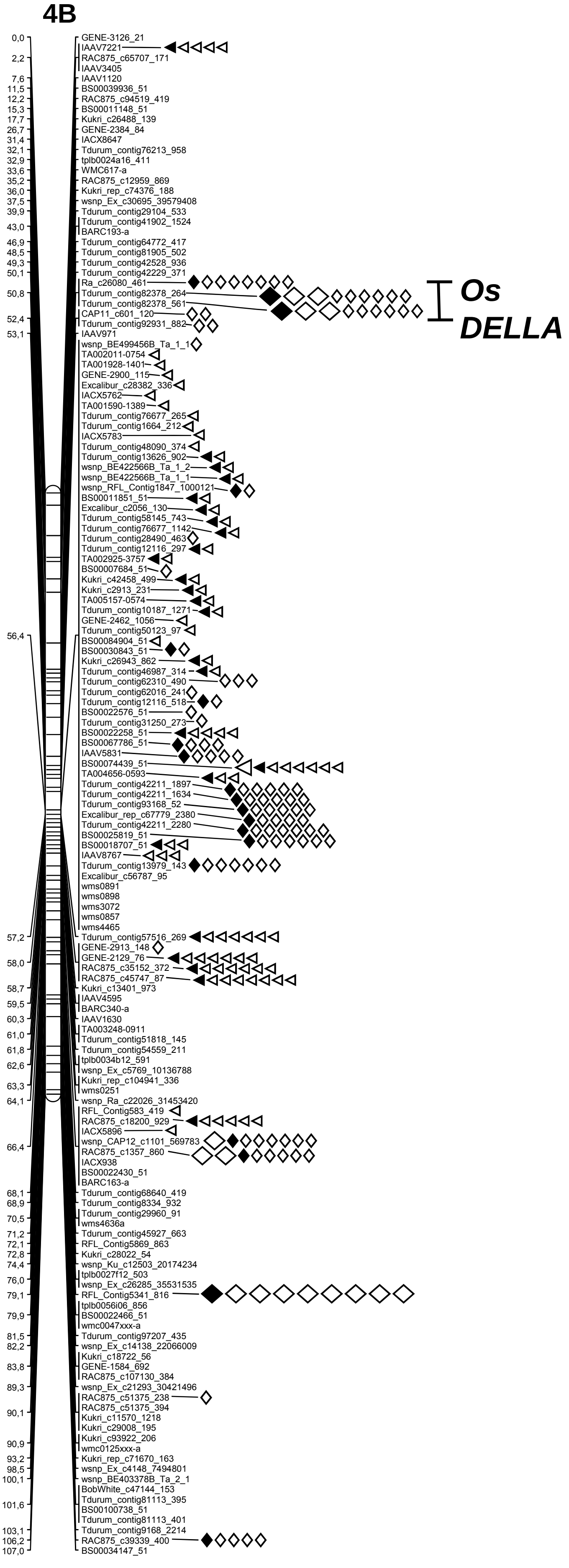
4B
0,0
2,2
7,6
11,5
12,2
15,3
17,7
26,7
31,4
32,1
32,9
33,6
35,2
36,0
37,5
39,9
43,0
46,9
48,5
49,3
50,1
50,8
52,4
53,1
56,4
57,2
58,0
58,7
59,5
60,3
61,0
61,8
62,6
63,3
64,1
66,4
68,1
68,9
70,5
71,2
72,1
72,8
74,4
76,0
79,1
79,9
81,5
82,2
83,8
89,3
90,1
90,9
93,2
98,5
100,1
101,6
103,1
106,2
107,0
GENE-3126_21
IAAV7221
RAC875_c65707_171
IAAV3405
IAAV1120
BS00039936_51
RAC875_c94519_419
BS00011148_51
Kukri_c26488_139
GENE-2384_84
IACX8647
Tdurum_contig76213_958
tplb0024a16_411
WMC617-a
RAC875_c12959_869
Kukri_rep_c74376_188
wsnp_Ex_c30695_39579408
Tdurum_contig29104_533
Tdurum_contig41902_1524
BARC193-a
Tdurum_contig64772_417
Tdurum_contig81905_502
Tdurum_contig42528_936
Tdurum_contig42229_371
Ra_c26080_461
Tdurum_contig82378_264
Tdurum_contig82378_561
CAP11_c601_120
Tdurum_contig92931_882
IAAV971
wsnp_BE499456B_Ta_1_1
TA002011-0754
TA001928-1401
GENE-2900_115
Excalibur_c28382_336
IACX5762
TA001590-1389
Tdurum_contig76677_265
Tdurum_contig1664_212
IACX5783
Tdurum_contig48090_374
Tdurum_contig13626_902
wsnp_BE422566B_Ta_1_2
wsnp_BE422566B_Ta_1_1
wsnp_RFL_Contig1847_1000121
BS00011851_51
Excalibur_c2056_130
Tdurum_contig58145_743
Tdurum_contig76677_1142
Tdurum_contig28490_463
Tdurum_contig12116_297
TA002925-3757
BS00007684_51
Kukri_c42458_499
Kukri_c2913_231
TA005157-0574
Tdurum_contig10187_1271
GENE-2462_1056
Tdurum_contig50123_97
BS00084904_51
BS00030843_51
Kukri_c26943_862
Tdurum_contig46987_314
Tdurum_contig62310_490
Tdurum_contig62016_241
Tdurum_contig12116_518
BS00022576_51
Tdurum_contig31250_273
BS00022258_51
BS00067786_51
IAAV5831
BS00074439_51
TA004656-0593
Tdurum_contig42211_1897
Tdurum_contig42211_1634
Tdurum_contig93168_52
Excalibur_rep_c67779_2380
Tdurum_contig42211_2280
BS00025819_51
BS00018707_51
IAAV8767
Tdurum_contig13979_143
Excalibur_c56787_95
wms0891
wms0898
wms3072
wms0857
wms4465
Tdurum_contig57516_269
GENE-2913_148
GENE-2129_76
RAC875_c35152_372
RAC875_c45747_87
Kukri_c13401_973
IAAV4595
BARC340-a
IAAV1630
TA003248-0911
Tdurum_contig51818_145
Tdurum_contig54559_211
tplb0034b12_591
wsnp_Ex_c5769_10136788
Kukri_rep_c104941_336
wms0251
wsnp_Ra_c22026_31453420
RFL_Contig583_419
RAC875_c18200_929
IACX5896
wsnp_CAP12_c1101_569783
RAC875_c1357_860
IACX938
BS00022430_51
BARC163-a
Tdurum_contig68640_419
Tdurum_contig8334_932
Tdurum_contig29960_91
wms4636a
Tdurum_contig45927_663
RFL_Contig5869_863
Kukri_c28022_54
wsnp_Ku_c12503_20174234
tplb0027f12_503
wsnp_Ex_c26285_35531535
RFL_Contig5341_816
tplb0056i06_856
BS00022466_51
wmc0047xxx-a
Tdurum_contig97207_435
wsnp_Ex_c14138_22066009
Kukri_c18722_56
GENE-1584_692
RAC875_c107130_384
wsnp_Ex_c21293_30421496
RAC875_c51375_238
RAC875_c51375_394
Kukri_c11570_1218
Kukri_c29008_195
Kukri_c93922_206
wmc0125xxx-a
Kukri_rep_c71670_163
wsnp_Ex_c4148_7494801
wsnp_BE403378B_Ta_2_1
BobWhite_c47144_153
Tdurum_contig81113_395
BS00100738_51
Tdurum_contig81113_401
Tdurum_contig9168_2214
RAC875_c39339_400
BS00034147_51
Os
DELLA

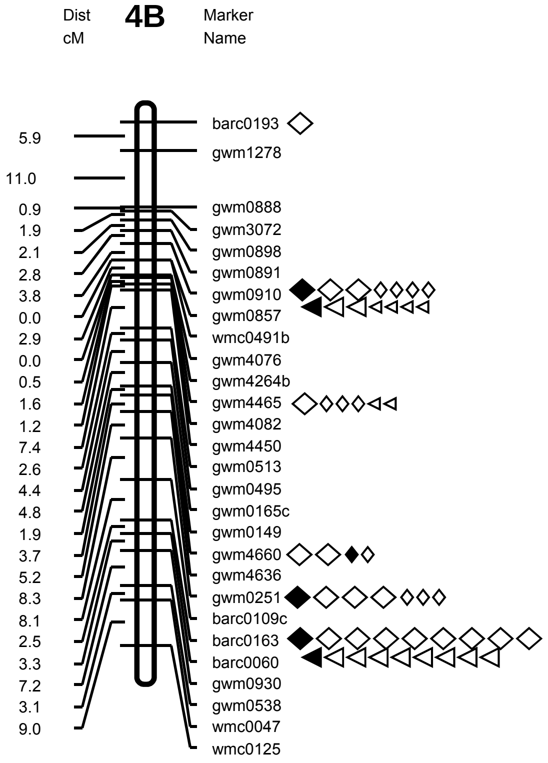
Dist
cM
4B
Marker
Name
5.9
11.0
0.9
1.9
2.1
2.8
3.8
0.0
2.9
0.0
0.5
1.6
1.2
7.4
2.6
4.4
4.8
1.9
3.7
5.2
8.3
8.1
2.5
3.3
7.2
3.1
9.0
barc0193
gwm1278
gwm0888
gwm3072
gwm0898
gwm0891
gwm0910
gwm0857
wmc0491b
gwm4076
gwm4264b
gwm4465
gwm4082
gwm4450
gwm0513
gwm0495
gwm0165c
gwm0149
gwm4660
gwm4636
gwm0251
barc0109c
barc0163
barc0060
gwm0930
gwm0538
wmc0047
wmc0125

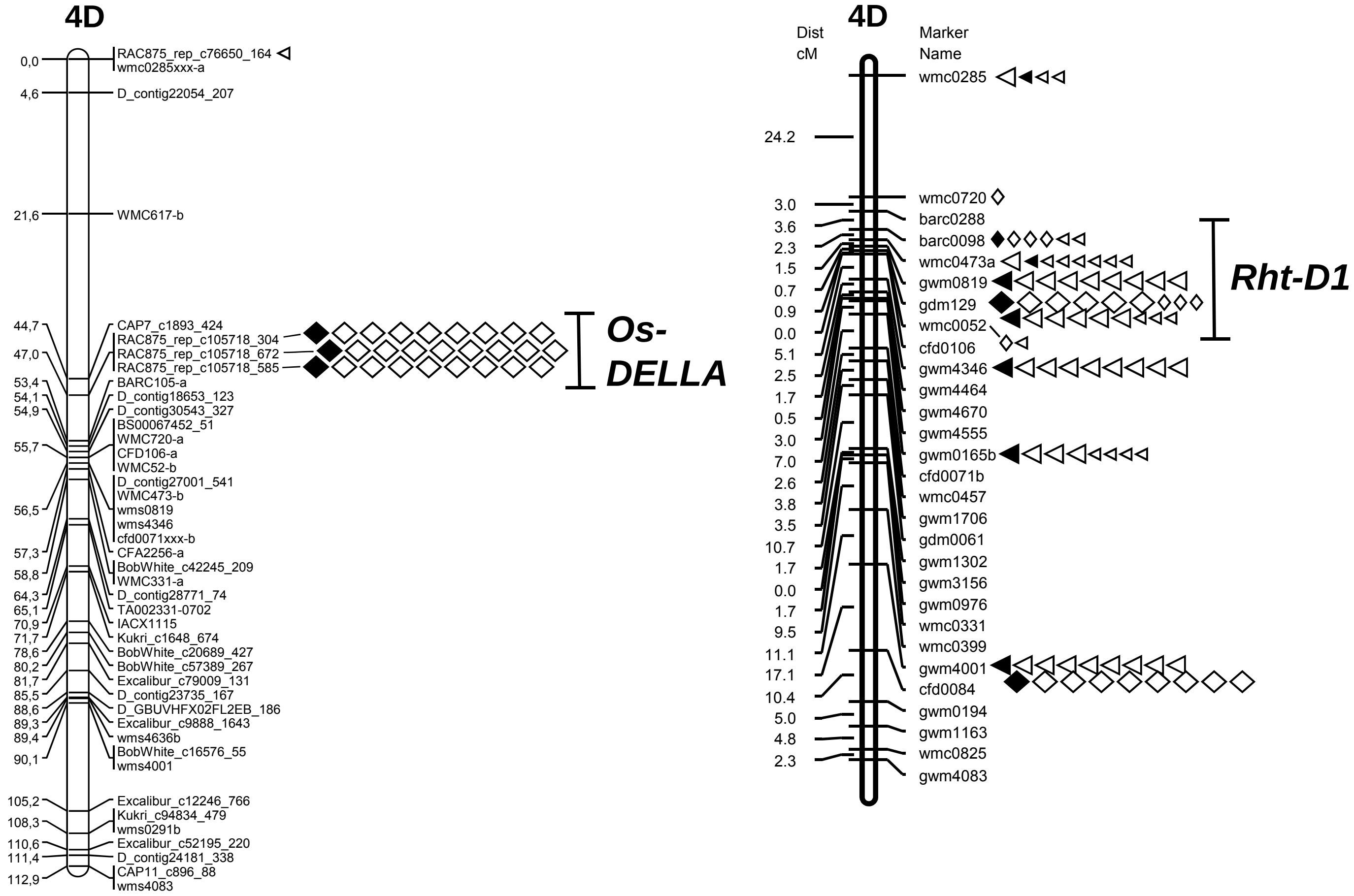
4D
RAC875_rep_c76650_164
wmc0285xxx-a
D_contig22054_207
WMC617-b
CAP7_c1893_424
RAC875_rep_c105718_304
RAC875_rep_c105718_672
RAC875_rep_c105718_585
Os-DELLA
4D
Dist cM
Marker Name
wmc0285
wmc0720
barc0288
barc0098
wmc0473a
gwm0819
gdm129
wmc0052
cfd0106
gwm4346
gwm4464
gwm4670
gwm4555
gwm0165b
cfd0071b
wmc0457
gwm1706
gdm0061
gwm1302
gwm3156
gwm0976
wmc0331
wmc0399
gwm4001
cfd0084
gwm0194
gwm1163
wmc0825
gwm4083
Rht-D1

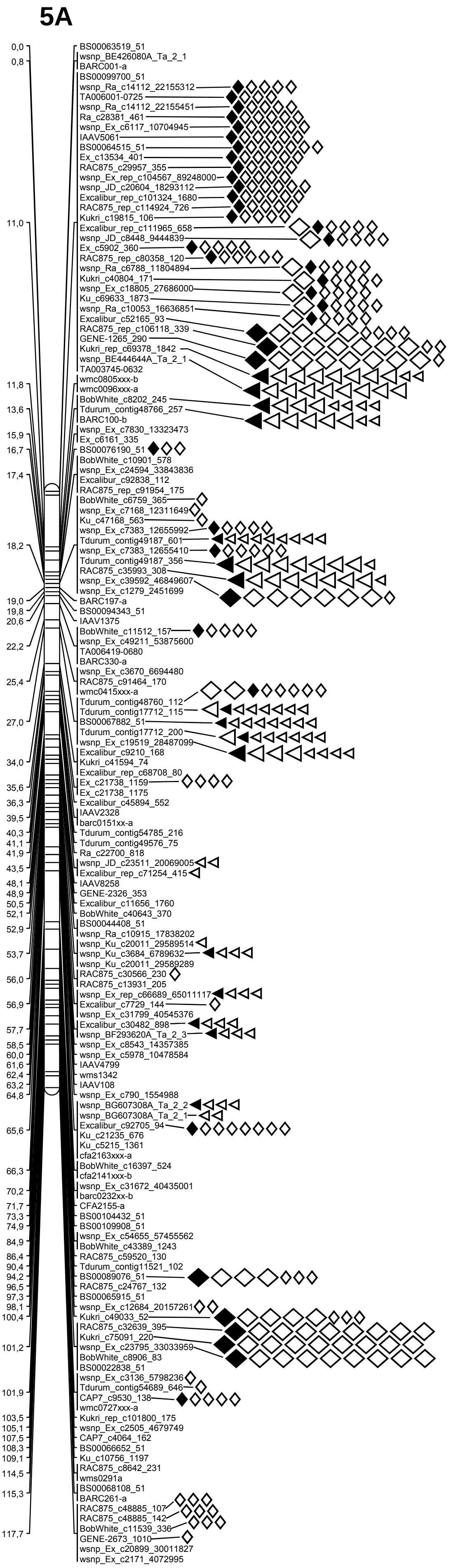
5A

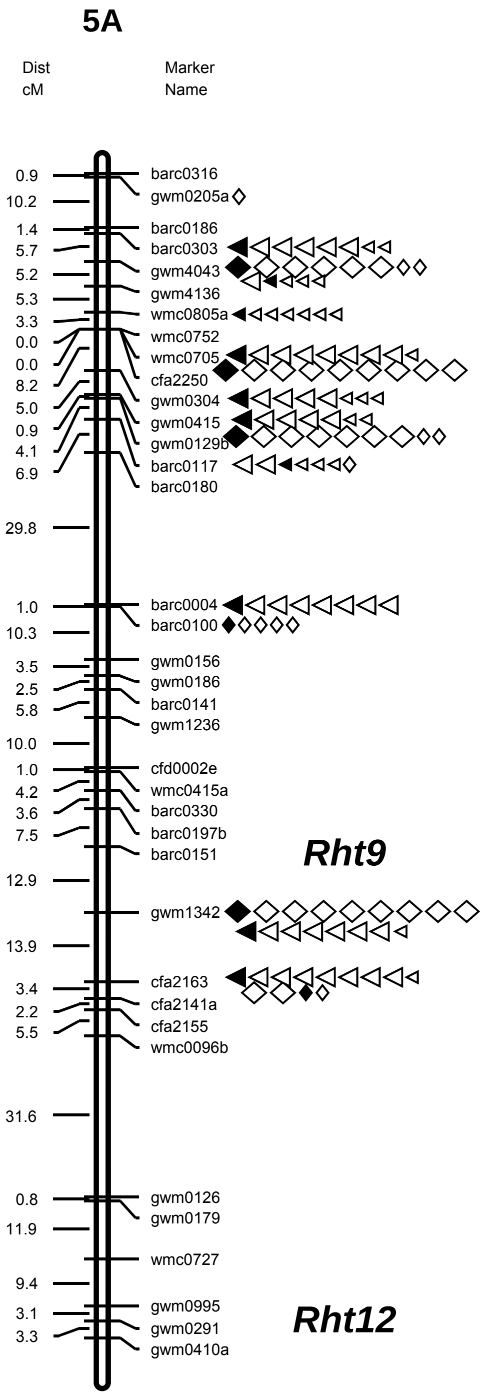
5A
Dist cM
Marker Name
0.9
10.2
1.4
5.7
5.2
5.3
3.3
0.0
0.0
8.2
5.0
0.9
4.1
6.9
29.8
1.0
10.3
3.5
2.5
5.8
10.0
1.0
4.2
3.6
7.5
12.9
13.9
3.4
2.2
5.5
31.6
0.8
11.9
9.4
3.1
3.3
barc0316
gwm0205a
barc0186
barc0303
gwm4043
gwm4136
wmc0805a
wmc0752
wmc0705
cfa2250
gwm0304
gwm0415
gwm0129b
barc0117
barc0180
barc0004
barc0100
gwm0156
gwm0186
barc0141
gwm1236
cfd0002e
wmc0415a
barc0330
barc0197b
barc0151
gwm1342
cfa2163
cfa2141a
cfa2155
wmc0096b
gwm0126
gwm0179
wmc0727
gwm0995
gwm0291
gwm0410a
Rht9
Rht12

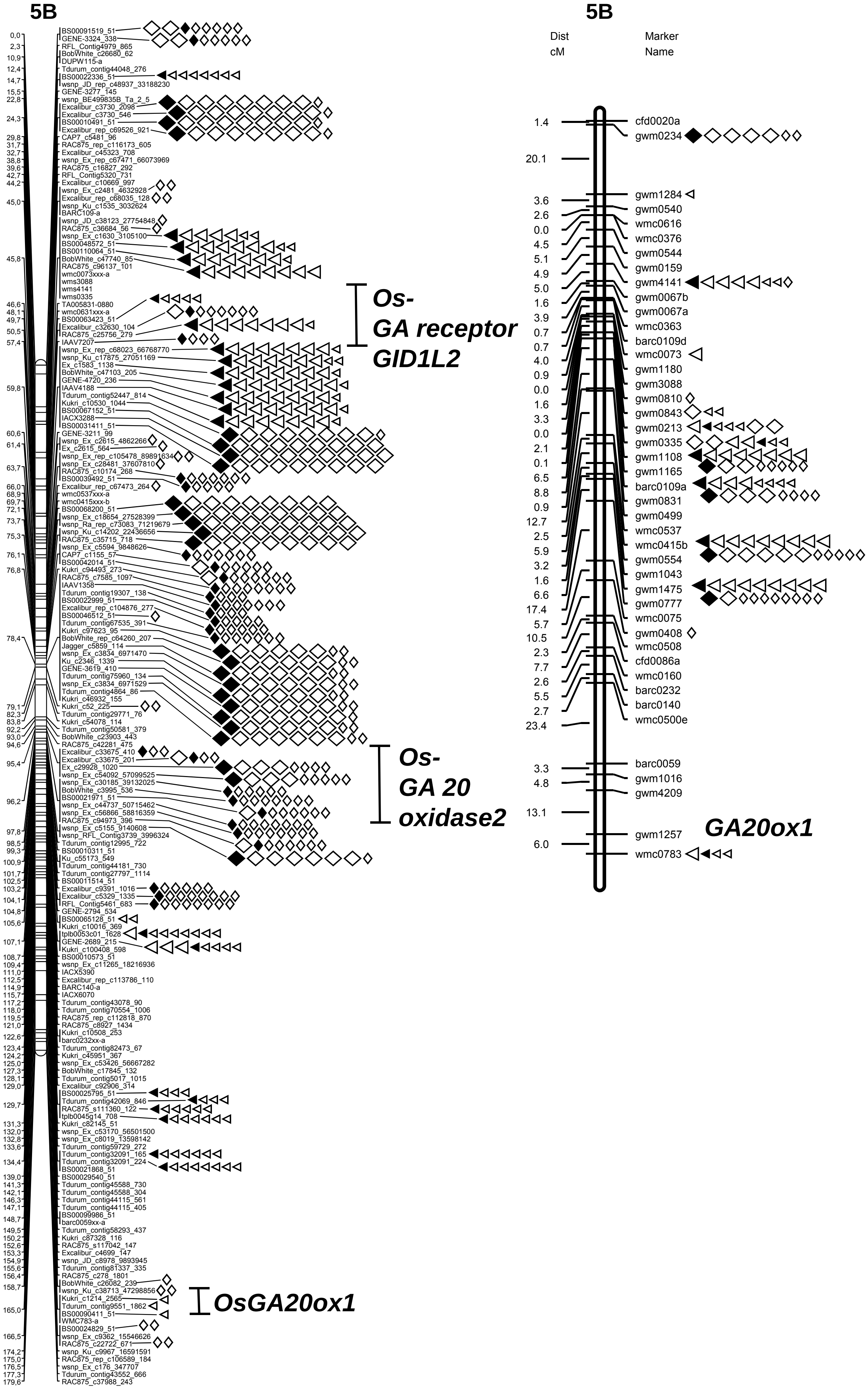

5B
BS00091519_51
GENE-3324_338
RFL_Contig4979_865
BobWhite_c26680_62
DUPW115-a
Tdurum_contig44048_276
BS00022336_51
wsnp_JD_rep_c48937_33188230
GENE-3277_145
wsnp_BE499835B_Ta_2_5
Excalibur_c3730_2098
Excalibur_c3730_546
BS00010491_51
Excalibur_rep_c69526_921
CAP7_c5481_96
RAC875_rep_c116173_605
Excalibur_c45323_708
wsnp_Ex_rep_c67471_66073969
RAC875_c16827_292
RFL_Contig5320_731
Excalibur_c10669_997
wsnp_Ex_c2481_4632928
Excalibur_rep_c68035_128
wsnp_Ku_c1535_3032624
BARC109-a
wsnp_JD_c38123_27754848
RAC875_c36684_56
wsnp_Ex_c1630_3105100
BS00048572_51
BS00110064_51
BobWhite_c47740_85
RAC875_c96137_101
wmc0073xxx-a
wms3088
wms4141
wms0335
TA005831-0880
wmc0631xxx-a
BS00063423_51
Excalibur_c32630_104
RAC875_c25756_279
IAAV7207
wsnp_Ex_rep_c68023_66768770
wsnp_Ku_c17875_27051169
Ex_c1583_1138
BobWhite_c47103_205
GENE-4720_236
IAAV4188
Tdurum_contig52447_814
Kukri_c10530_1044
BS00067152_51
IACX3288
BS00031411_51
GENE-3211_99
wsnp_Ex_c2615_4862266
Ex_c2615_564
wsnp_Ex_rep_c105478_89891634
wsnp_Ex_c28481_37607810
RAC875_c10174_268
BS00039492_51
Excalibur_rep_c67473_264
wmc0537xxx-a
wmc0415xxx-b
BS00068200_51
wsnp_Ex_c18654_27528399
wsnp_Ra_rep_c73083_71219679
wsnp_Ku_c14202_22436656
RAC875_c35715_718
wsnp_Ex_c5594_9848626
CAP7_c1155_57
BS00042014_51
Kukri_c94493_273
RAC875_c7585_1097
IAAV1358
Tdurum_contig19307_138
BS00022999_51
Excalibur_rep_c104876_277
BS00046512_51
Tdurum_contig67535_391
Kukri_c97623_95
BobWhite_rep_c64260_207
Jagger_c5859_114
wsnp_Ex_c3834_6971470
Ku_c2346_1339
GENE-3619_410
Tdurum_contig75960_134
wsnp_Ex_c3834_6971529
Tdurum_contig4864_86
Kukri_c46932_155
Kukri_c52_225
Tdurum_contig29771_76
Kukri_c54078_114
Tdurum_contig50581_379
BobWhite_c23903_443
RAC875_c42281_475
Excalibur_c33675_410
Excalibur_c33675_201
Ex_c29928_1020
wsnp_Ex_c54092_57099525
wsnp_Ex_c30185_39132025
BobWhite_c3995_536
BS00021971_51
wsnp_Ex_c44737_50715462
wsnp_Ex_c56866_58816359
RAC875_c94973_396
wsnp_Ex_c5155_9140608
wsnp_RFL_Contig3739_3996324
Tdurum_contig12995_722
BS00010311_51
Ku_c55173_549
Tdurum_contig44181_730
Tdurum_contig27797_1114
BS00011514_51
Excalibur_c9391_1016
Excalibur_c5329_1335
RFL_Contig5461_683
GENE-2794_534
BS00065128_51
Kukri_c10016_369
tplb0053c01_1628
GENE-2689_215
Kukri_c100408_598
BS00010573_51
wsnp_Ex_c11265_18216936
IACX5390
Excalibur_rep_c113786_110
BARC140-a
IACX6070
Tdurum_contig43078_90
Tdurum_contig70554_1006
RAC875_rep_c112818_870
RAC875_c8927_1434
Kukri_c10508_253
barc0232xx-a
Tdurum_contig82473_67
Kukri_c45951_367
wsnp_Ex_c53426_56667282
BobWhite_c17845_132
Tdurum_contig5017_1015
Excalibur_c92906_314
BS00025795_51
Tdurum_contig42069_846
RAC875_s111360_122
tplb0045g14_708
Kukri_c82145_51
wsnp_Ex_c53170_56501500
wsnp_Ex_c8019_13598142
Tdurum_contig59729_272
Tdurum_contig32091_165
Tdurum_contig32091_224
BS00021868_51
BS00029540_51
Tdurum_contig45588_730
Tdurum_contig45588_304
Tdurum_contig44115_561
Tdurum_contig44115_405
BS00099986_51
barc0059xx-a
Tdurum_contig58293_437
Kukri_c87328_116
RAC875_s117042_147
Excalibur_c4699_147
wsnp_JD_c8978_9893945
Tdurum_contig81337_335
RAC875_c278_1801
BobWhite_c26082_239
wsnp_Ku_c38713_47298856
Kukri_c1214_2565
Tdurum_contig9551_1862
BS00090411_51
WMC783-a
BS00024829_51
wsnp_Ex_c9362_15546626
RAC875_c22722_671
wsnp_Ku_c9967_16591591
RAC875_rep_c106589_184
wsnp_Ex_c176_347707
Tdurum_contig43552_666
RAC875_c37988_243
Os-
GA receptor
GID1L2
Os-
GA 20
oxidase2
OsGA20ox1
5B
Dist
cM
Marker
Name
1.4 cfd0020a
gwm0234
20.1
3.6 gwm1284
2.6 gwm0540
0.0 wmc0616
4.5 wmc0376
5.1 gwm0544
4.9 gwm0159
5.0 gwm4141
1.6 gwm0067b
3.9 gwm0067a
0.7 wmc0363
0.7 barc0109d
4.0 wmc0073
0.9 gwm1180
0.0 gwm3088
1.6 gwm0810
3.3 gwm0843
0.0 gwm0213
2.1 gwm0335
0.1 gwm1108
6.5 gwm1165
8.8 barc0109a
0.9 gwm0831
12.7 gwm0499
2.5 wmc0537
5.9 wmc0415b
3.2 gwm0554
1.6 gwm1043
6.6 gwm1475
17.4 gwm0777
5.7 wmc0075
10.5 gwm0408
2.3 wmc0508
7.7 cfd0086a
2.6 wmc0160
5.5 barc0232
2.7 barc0140
23.4 wmc0500e
3.3 barc0059
4.8 gwm1016
13.1 gwm4209
6.0 gwm1257
wmc0783
GA20ox1

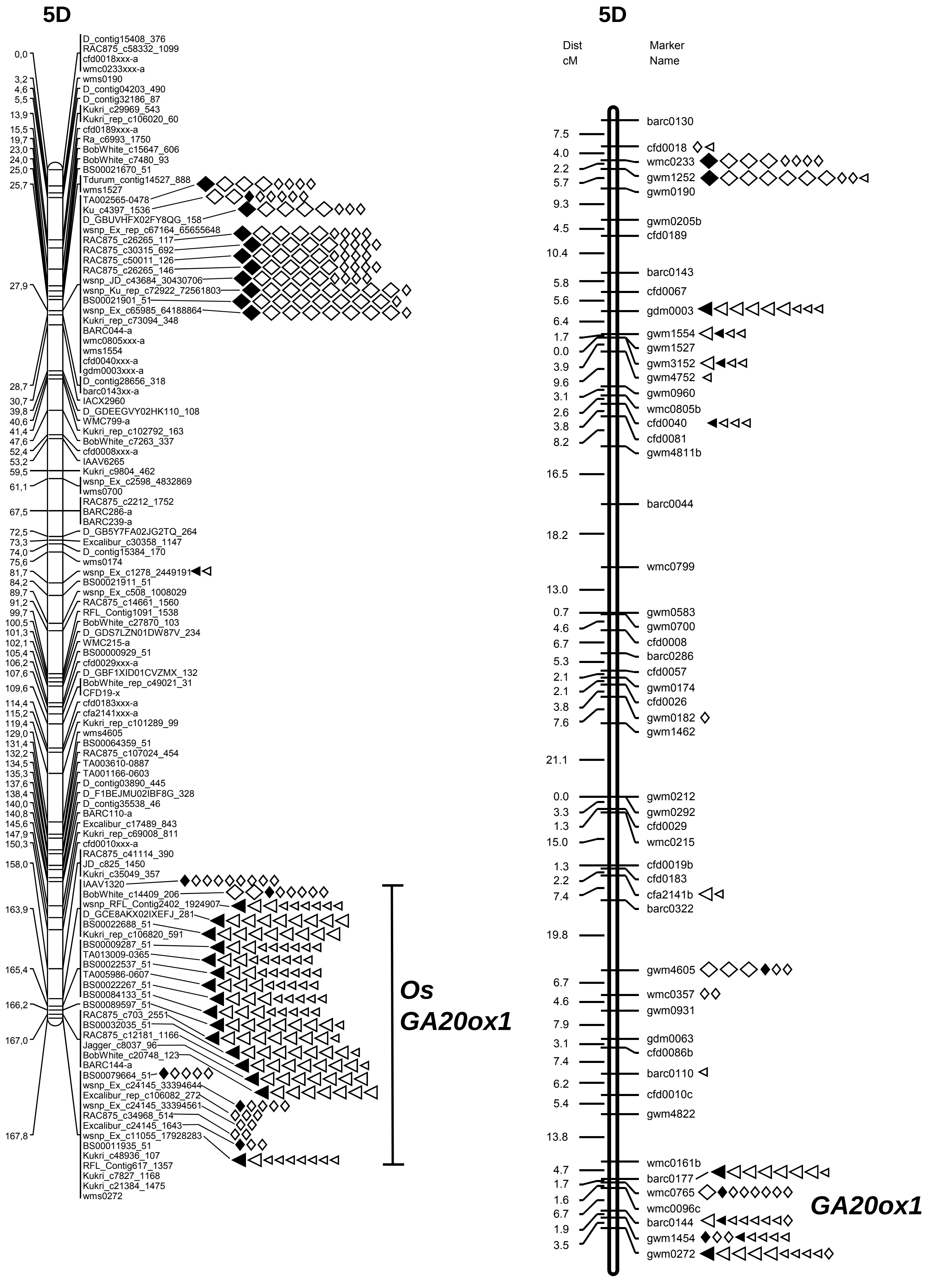

5D
Os
GA20ox1
5D
Dist cM
Marker Name
barc0130
cfd0018
wmc0233
gwm1252
gwm0190
gwm0205b
cfd0189
barc0143
cfd0067
gdm0003
gwm1554
gwm1527
gwm3152
gwm4752
gwm0960
wmc0805b
cfd0040
cfd0081
gwm4811b
barc0044
wmc0799
gwm0583
gwm0700
cfd0008
barc0286
cfd0057
gwm0174
cfd0026
gwm0182
gwm1462
gwm0212
gwm0292
cfd0029
wmc0215
cfd0019b
cfd0183
cfa2141b
barc0322
gwm4605
wmc0357
gwm0931
gdm0063
cfd0086b
barc0110
cfd0010c
gwm4822
wmc0161b
barc0177
wmc0765
wmc0096c
barc0144
gwm1454
gwm0272
GA20ox1

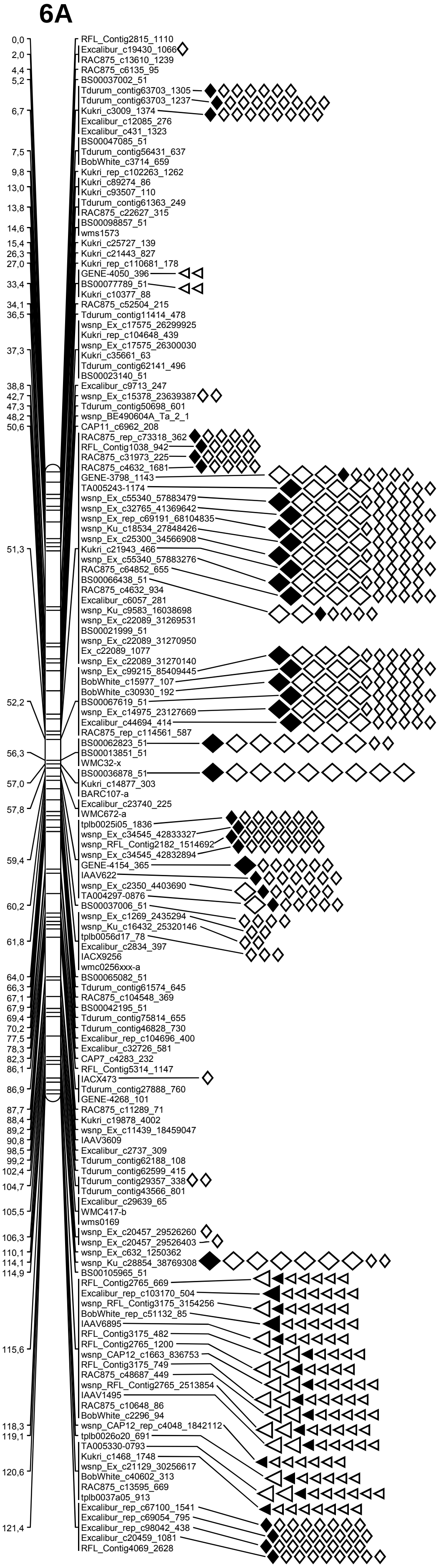
6A
0,0
2,0
4,4
5,2
6,7
7,5
9,8
13,0
13,8
14,6
15,4
26,3
27,0
33,4
34,1
36,5
37,3
38,8
42,7
47,3
48,2
50,6
51,3
52,2
56,3
57,0
57,8
59,4
60,2
61,8
64,0
66,3
67,1
67,9
69,4
70,2
77,5
78,3
82,3
86,1
86,9
87,7
88,4
89,2
90,8
98,5
99,2
102,4
104,7
105,5
106,3
110,1
114,1
114,9
115,6
118,3
119,1
120,6
121,4
RFL_Contig2815_1110
Excalibur_c19430_1066
RAC875_c13610_1239
RAC875_c6135_95
BS00037002_51
Tdurum_contig63703_1305
Tdurum_contig63703_1237
Kukri_c3009_1374
Excalibur_c12085_276
Excalibur_c431_1323
BS00047085_51
Tdurum_contig56431_637
BobWhite_c3714_659
Kukri_rep_c102263_1262
Kukri_c89274_86
Kukri_c93507_110
Tdurum_contig61363_249
RAC875_c22627_315
BS00098857_51
wms1573
Kukri_c25727_139
Kukri_c21443_827
Kukri_rep_c110681_178
GENE-4050_396
BS00077789_51
Kukri_c10377_88
RAC875_c52504_215
Tdurum_contig11414_478
wsnp_Ex_c17575_26299925
Kukri_rep_c104648_439
wsnp_Ex_c17575_26300030
Kukri_c35661_63
Tdurum_contig62141_496
BS00023140_51
Excalibur_c9713_247
wsnp_Ex_c15378_23639387
Tdurum_contig50698_601
wsnp_BE490604A_Ta_2_1
CAP11_c6962_208
RAC875_rep_c73318_362
RFL_Contig1038_942
RAC875_c31973_225
RAC875_c4632_1681
GENE-3798_1143
TA005243-1174
wsnp_Ex_c55340_57883479
wsnp_Ex_c32765_41369642
wsnp_Ex_rep_c69191_68104835
wsnp_Ku_c18534_27848426
wsnp_Ex_c25300_34566908
Kukri_c21943_466
wsnp_Ex_c55340_57883276
RAC875_c64852_655
BS00066438_51
RAC875_c4632_934
Excalibur_c6057_281
wsnp_Ku_c9583_16038698
wsnp_Ex_c22089_31269531
BS00021999_51
wsnp_Ex_c22089_31270950
Ex_c22089_1077
wsnp_Ex_c22089_31270140
wsnp_Ex_c99215_85409445
BobWhite_c15977_107
BobWhite_c30930_192
BS00067619_51
wsnp_Ex_c14975_23127669
Excalibur_c44694_414
RAC875_rep_c114561_587
BS00062823_51
BS00013851_51
WMC32-x
BS00036878_51
Kukri_c14877_303
BARC107-a
Excalibur_c23740_225
WMC672-a
tplb0025i05_1836
wsnp_Ex_c34545_42833327
wsnp_RFL_Contig2182_1514692
wsnp_Ex_c34545_42832894
GENE-4154_365
IAAV622
wsnp_Ex_c2350_4403690
TA004297-0876
BS00037006_51
wsnp_Ex_c1269_2435294
wsnp_Ku_c16432_25320146
tplb0056d17_78
Excalibur_c2834_397
IACX9256
wmc0256xxx-a
BS00065082_51
Tdurum_contig61574_645
RAC875_c104548_369
BS00042195_51
Tdurum_contig75814_655
Tdurum_contig46828_730
Excalibur_rep_c104696_400
Excalibur_c32726_581
CAP7_c4283_232
RFL_Contig5314_1147
IACX473
Tdurum_contig27888_760
GENE-4268_101
RAC875_c11289_71
Kukri_c19878_4002
wsnp_Ex_c11439_18459047
IAAV3609
Excalibur_c2737_309
Tdurum_contig62188_108
Tdurum_contig62599_415
Tdurum_contig29357_338
Tdurum_contig43566_801
Excalibur_c29639_65
WMC417-b
wms0169
wsnp_Ex_c20457_29526260
wsnp_Ex_c20457_29526403
wsnp_Ex_c632_1250362
wsnp_Ku_c28854_38769308
BS00105965_51
RFL_Contig2765_669
Excalibur_rep_c103170_504
wsnp_RFL_Contig3175_3154256
BobWhite_rep_c51132_85
IAAV6895
RFL_Contig3175_482
RFL_Contig2765_1200
wsnp_CAP12_c1663_836753
RFL_Contig3175_749
RAC875_c48687_449
wsnp_RFL_Contig2765_2513854
IAAV1495
RAC875_c10648_86
BobWhite_c2296_94
wsnp_CAP12_rep_c4048_1842112
tplb0026o20_691
TA005330-0793
Kukri_c1468_1748
wsnp_Ex_c21129_30256617
BobWhite_c40602_313
RAC875_c13595_669
tplb0037a05_913
Excalibur_rep_c67100_1541
Excalibur_rep_c69054_795
Excalibur_rep_c98042_438
Excalibur_c20459_1081
RFL_Contig4069_2628

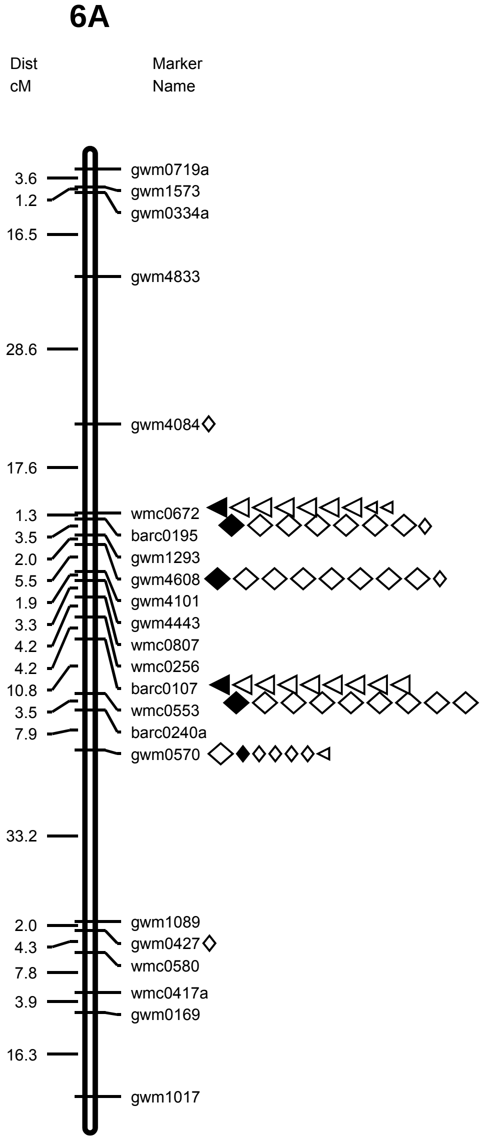
6A
Dist cM
Marker Name
3.6
1.2
16.5
28.6
17.6
1.3
3.5
2.0
5.5
1.9
3.3
4.2
4.2
10.8
3.5
7.9
33.2
2.0
4.3
7.8
3.9
16.3
gwm0719a
gwm1573
gwm0334a
gwm4833
gwm4084
wmc0672
barc0195
gwm1293
gwm4608
gwm4101
gwm4443
wmc0807
wmc0256
barc0107
wmc0553
barc0240a
gwm0570
gwm1089
gwm0427
wmc0580
wmc0417a
gwm0169
gwm1017

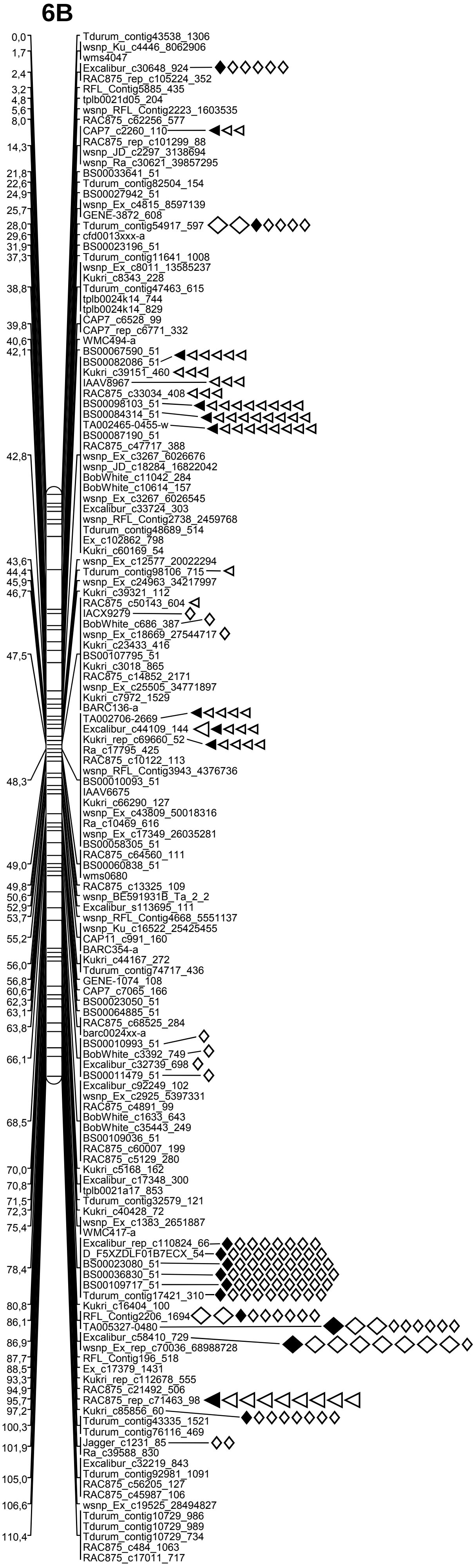

6B
0,0
1,7
2,4
3,2
4,8
5,6
8,0
14,3
21,8
22,6
24,9
25,7
28,0
29,6
31,9
37,3
38,8
39,8
40,6
42,1
42,8
43,6
44,4
45,9
46,7
47,5
48,3
49,0
49,8
50,6
52,9
53,7
55,2
56,0
56,8
60,6
62,3
63,1
63,8
66,1
68,5
70,0
70,8
71,5
72,3
75,4
78,4
80,8
86,1
86,9
87,7
88,5
93,3
94,9
95,7
97,2
100,3
101,9
105,0
106,6
110,4
Tdurum_contig43538_1306
wsnp_Ku_c4446_8062906
wms4047
Excalibur_c30648_924
RAC875_rep_c105224_352
RFL_Contig5885_435
tplb0021d05_204
wsnp_RFL_Contig2223_1603535
RAC875_c62256_577
CAP7_c2260_110
RAC875_rep_c101299_88
wsnp_JD_c2297_3138694
wsnp_Ra_c30621_39857295
BS00033641_51
Tdurum_contig82504_154
BS00027942_51
wsnp_Ex_c4815_8597139
GENE-3872_608
Tdurum_contig54917_597
cfd0013xxx-a
BS00023196_51
Tdurum_contig11641_1008
wsnp_Ex_c8011_13585237
Kukri_c8343_228
Tdurum_contig47463_615
tplb0024k14_744
tplb0024k14_829
CAP7_c6528_99
CAP7_rep_c6771_332
WMC494-a
BS00067590_51
BS00082086_51
Kukri_c39151_460
IAAV8967
RAC875_c33034_408
BS00098103_51
BS00084314_51
TA002465-0455-w
BS00087190_51
RAC875_c47717_388
wsnp_Ex_c3267_6026676
wsnp_JD_c18284_16822042
BobWhite_c11042_284
BobWhite_c10614_157
wsnp_Ex_c3267_6026545
Excalibur_c33724_303
wsnp_RFL_Contig2738_2459768
Tdurum_contig48689_514
Ex_c102862_798
Kukri_c60169_54
wsnp_Ex_c12577_20022294
Tdurum_contig98106_715
wsnp_Ex_c24963_34217997
Kukri_c39321_112
RAC875_c50143_604
IACX9279
BobWhite_c686_387
wsnp_Ex_c18669_27544717
Kukri_c23433_416
BS00107795_51
Kukri_c3018_865
RAC875_c14852_2171
wsnp_Ex_c25505_34771897
Kukri_c7972_1529
BARC136-a
TA002706-2669
Excalibur_c44109_144
Kukri_rep_c69660_52
Ra_c17795_425
RAC875_c10122_113
wsnp_RFL_Contig3943_4376736
BS00010093_51
IAAV6675
Kukri_c66290_127
wsnp_Ex_c43809_50018316
Ra_c10469_616
wsnp_Ex_c17349_26035281
BS00058305_51
RAC875_c64560_111
BS00060838_51
wms0680
RAC875_c13325_109
wsnp_BE591931B_Ta_2_2
Excalibur_s113695_111
wsnp_RFL_Contig4668_5551137
wsnp_Ku_c16522_25425455
CAP11_c991_160
BARC354-a
Kukri_c44167_272
Tdurum_contig74717_436
GENE-1074_108
CAP7_c7065_166
BS00023050_51
BS00064885_51
RAC875_c68525_284
barc0024xx-a
BS00010993_51
BobWhite_c3392_749
Excalibur_c32739_698
BS00011479_51
Excalibur_c92249_102
wsnp_Ex_c2925_5397331
RAC875_c4891_99
BobWhite_c1633_643
BobWhite_c35443_249
BS00109036_51
RAC875_c60007_199
RAC875_c5129_280
Kukri_c5168_162
Excalibur_c17348_300
tplb0021a17_853
Tdurum_contig32579_121
Kukri_c40428_72
wsnp_Ex_c1383_2651887
WMC417-a
Excalibur_rep_c110824_66
D_F5XZDLF01B7ECX_54
BS00023080_51
BS00036830_51
BS00109717_51
Tdurum_contig17421_310
Kukri_c16404_100
RFL_Contig2206_1694
TA005327-0480
Excalibur_c58410_729
wsnp_Ex_rep_c70036_68988728
RFL_Contig196_518
Ex_c17379_1431
Kukri_rep_c112678_555
RAC875_c21492_506
RAC875_rep_c71463_98
Kukri_c85856_60
Tdurum_contig43335_1521
Tdurum_contig76116_469
Jagger_c1231_85
Ra_c39588_830
Excalibur_c32219_843
Tdurum_contig92981_1091
RAC875_c56205_127
RAC875_c45987_106
wsnp_Ex_c19525_28494827
Tdurum_contig10729_986
Tdurum_contig10729_989
Tdurum_contig10729_734
RAC875_c484_1063
RAC875_c17011_717

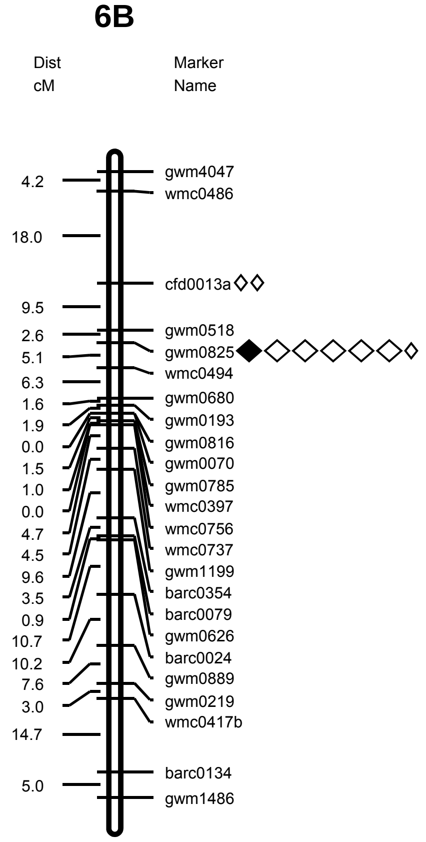

6B
Dist
cM
Marker
Name
4.2
18.0
9.5
2.6
5.1
6.3
1.6
1.9
0.0
1.5
1.0
0.0
4.7
4.5
9.6
3.5
0.9
10.7
10.2
7.6
3.0
14.7
5.0
gwm4047
wmc0486
cfd0013a
gwm0518
gwm0825
wmc0494
gwm0680
gwm0193
gwm0816
gwm0070
gwm0785
wmc0397
wmc0756
wmc0737
gwm1199
barc0354
barc0079
gwm0626
barc0024
gwm0889
gwm0219
wmc0417b
barc0134
gwm1486

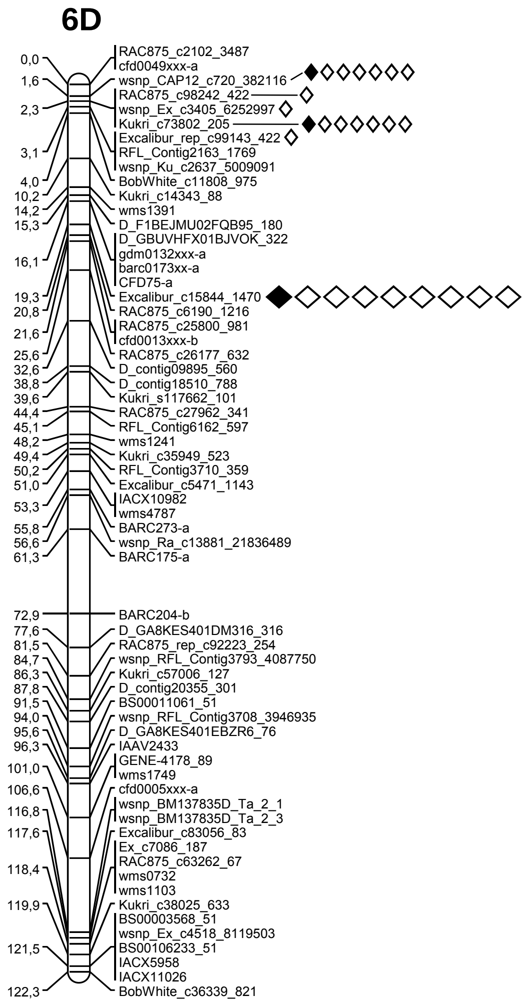
6D
0,0
1,6
2,3
3,1
4,0
10,2
14,2
15,3
16,1
19,3
20,8
21,6
25,6
32,6
38,8
39,6
44,4
45,1
48,2
49,4
50,2
51,0
53,3
55,8
56,6
61,3
72,9
77,6
81,5
84,7
86,3
87,8
91,5
94,0
95,6
96,3
101,0
106,6
116,8
117,6
118,4
119,9
121,5
122,3
RAC875_c2102_3487
cfd0049xxx-a
wsnp_CAP12_c720_382116
RAC875_c98242_422
wsnp_Ex_c3405_6252997
Kukri_c73802_205
Excalibur_rep_c99143_422
RFL_Contig2163_1769
wsnp_Ku_c2637_5009091
BobWhite_c11808_975
Kukri_c14343_88
wms1391
D_F1BEJMU02FQB95_180
D_GBUVHFX01BJVOK_322
gdm0132xxx-a
barc0173xx-a
CFD75-a
Excalibur_c15844_1470
RAC875_c6190_1216
RAC875_c25800_981
cfd0013xxx-b
RAC875_c26177_632
D_contig09895_560
D_contig18510_788
Kukri_s117662_101
RAC875_c27962_341
RFL_Contig6162_597
wms1241
Kukri_c35949_523
RFL_Contig3710_359
Excalibur_c5471_1143
IACX10982
wms4787
BARC273-a
wsnp_Ra_c13881_21836489
BARC175-a
BARC204-b
D_GA8KES401DM316_316
RAC875_rep_c92223_254
wsnp_RFL_Contig3793_4087750
Kukri_c57006_127
D_contig20355_301
BS00011061_51
wsnp_RFL_Contig3708_3946935
D_GA8KES401EBZR6_76
IAAV2433
GENE-4178_89
wms1749
cfd0005xxx-a
wsnp_BM137835D_Ta_2_1
wsnp_BM137835D_Ta_2_3
Excalibur_c83056_83
Ex_c7086_187
RAC875_c63262_67
wms0732
wms1103
Kukri_c38025_633
BS00003568_51
wsnp_Ex_c4518_8119503
BS00106233_51
IACX5958
IACX11026
BobWhite_c36339_821

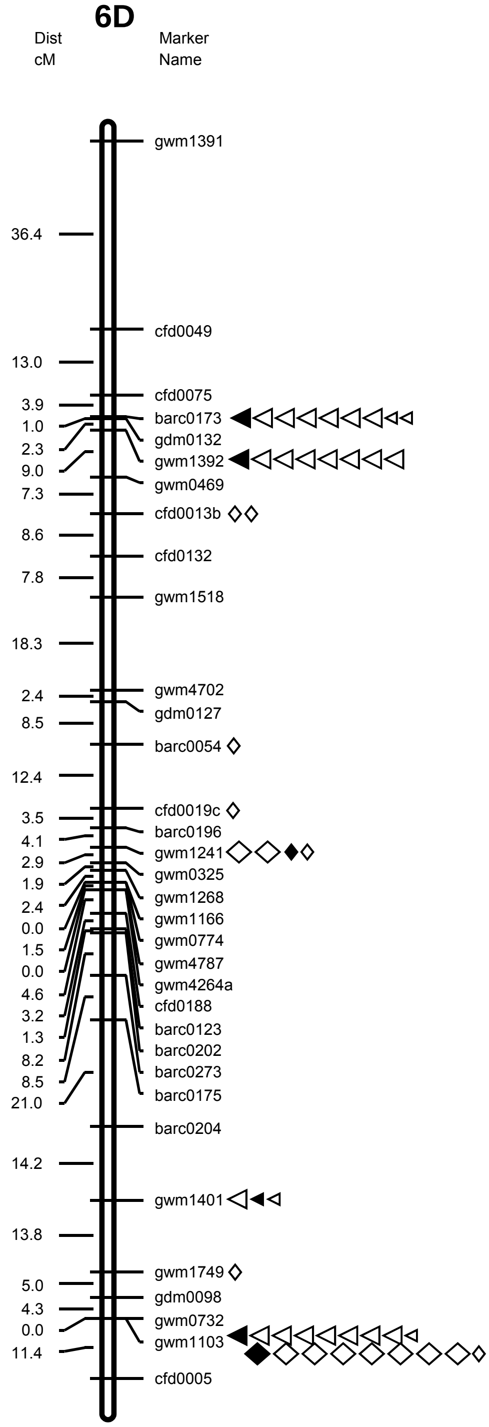
6D
Dist
cM
Marker
Name
gwm1391
36.4
cfd0049
13.0
cfd0075
3.9
barc0173
1.0
gdm0132
2.3
gwm1392
9.0
gwm0469
7.3
cfd0013b
8.6
cfd0132
7.8
gwm1518
18.3
gwm4702
2.4
gdm0127
8.5
barc0054
12.4
cfd0019c
3.5
barc0196
4.1
gwm1241
2.9
gwm0325
1.9
gwm1268
2.4
gwm1166
0.0
gwm0774
1.5
gwm4787
0.0
gwm4264a
4.6
cfd0188
3.2
barc0123
1.3
barc0202
8.2
barc0273
8.5
barc0175
21.0
barc0204
14.2
gwm1401
13.8
gwm1749
5.0
gdm0098
4.3
gwm0732
0.0
gwm1103
11.4
cfd0005

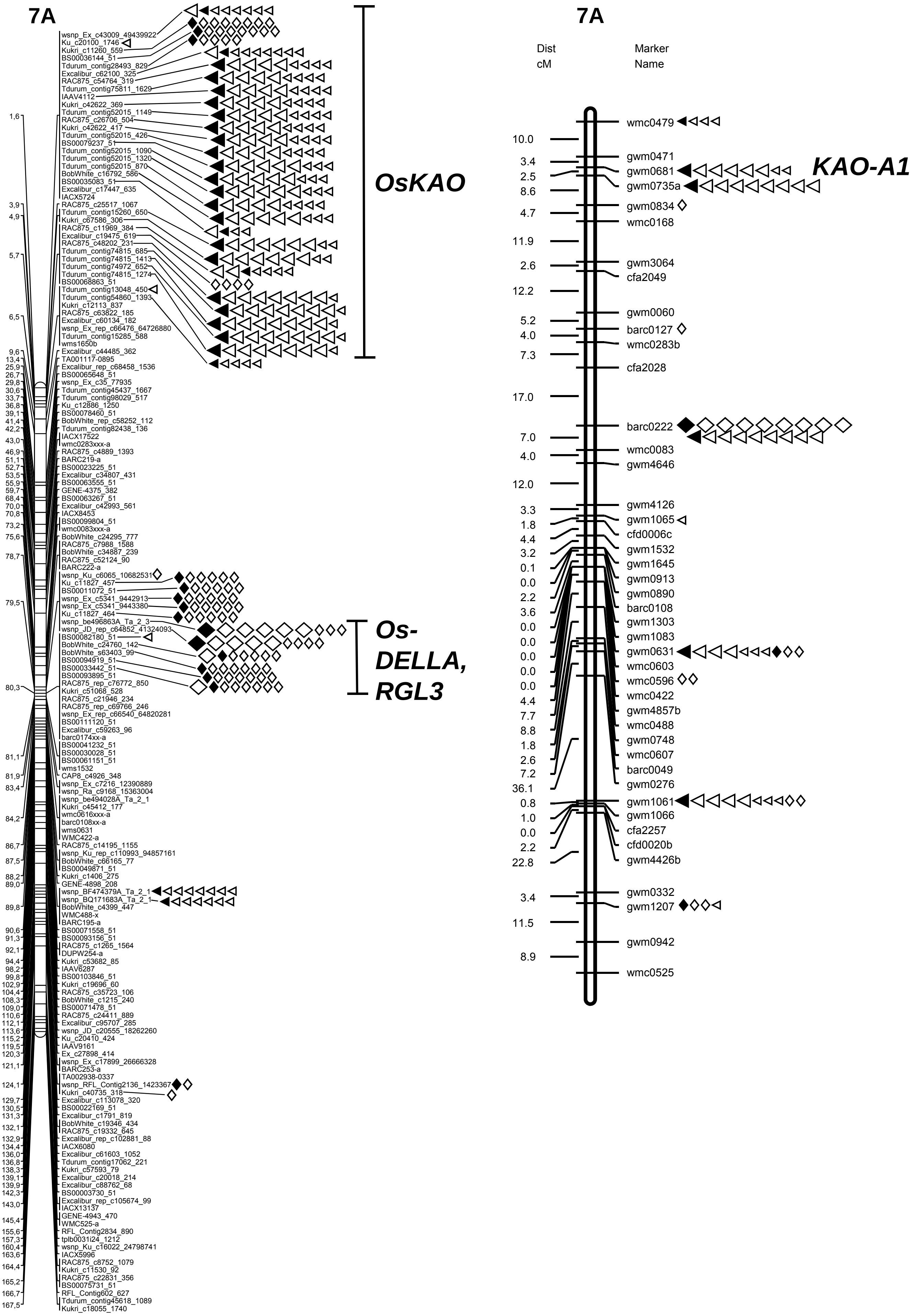
7A
OsKAO
Os-
DELLA,
RGL3
7A
Dist
cM
Marker
Name
KAO-A1
wmc0479
gwm0471
gwm0681
gwm0735a
gwm0834
wmc0168
gwm3064
cfa2049
gwm0060
barc0127
wmc0283b
cfa2028
barc0222
wmc0083
gwm4646
gwm4126
gwm1065
cfd0006c
gwm1532
gwm1645
gwm0913
gwm0890
barc0108
gwm1303
gwm1083
gwm0631
wmc0603
wmc0596
wmc0422
gwm4857b
wmc0488
gwm0748
wmc0607
barc0049
gwm0276
gwm1061
gwm1066
cfa2257
cfd0020b
gwm4426b
gwm0332
gwm1207
gwm0942
wmc0525
10.0
3.4
2.5
8.6
4.7
11.9
2.6
12.2
5.2
4.0
7.3
17.0
7.0
4.0
12.0
3.3
1.8
4.4
3.2
0.1
0.0
2.2
3.6
0.0
0.0
0.0
0.0
0.0
4.4
7.7
8.8
1.8
2.6
7.2
36.1
0.8
1.0
0.0
2.2
22.8
3.4
11.5
8.9

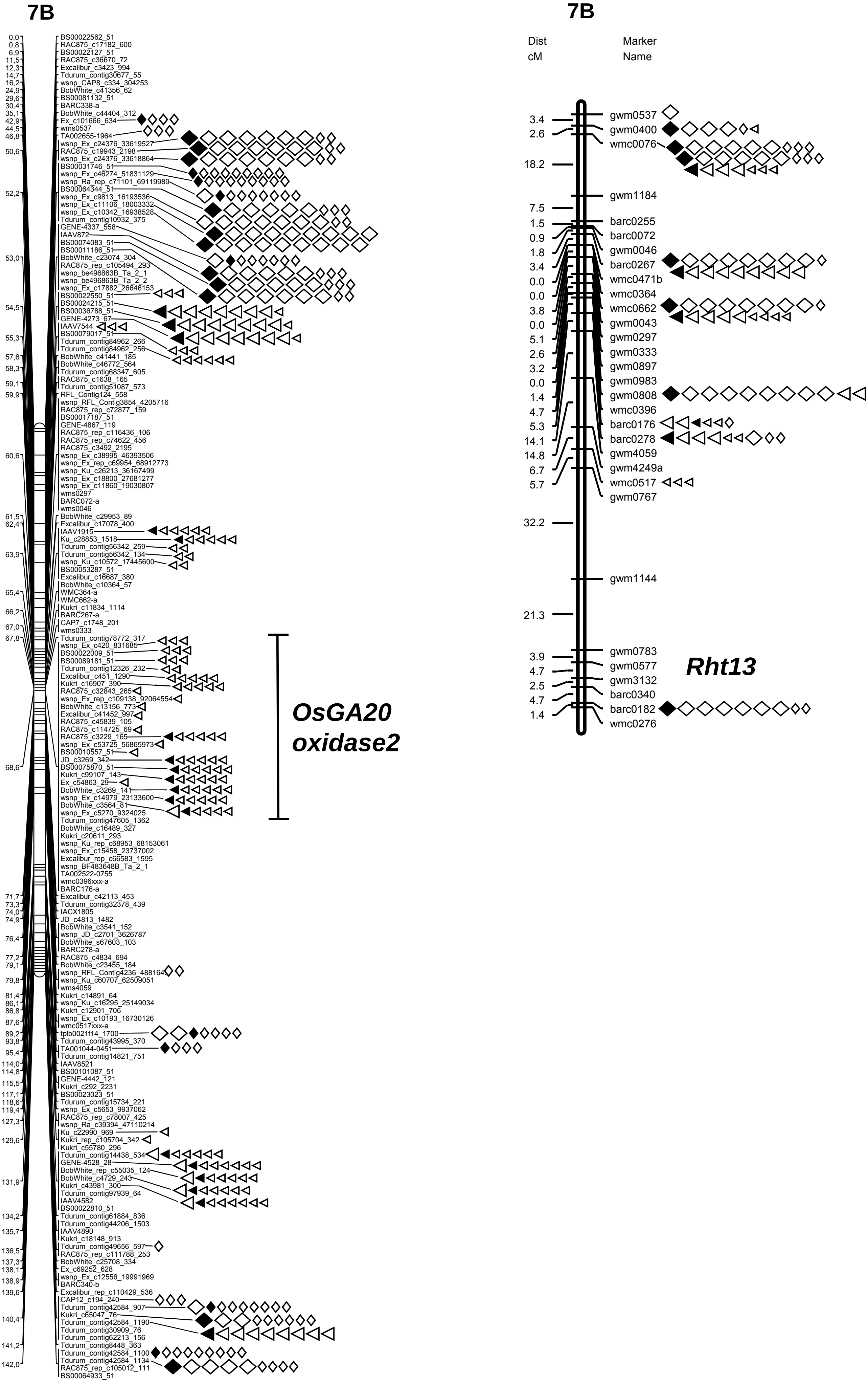
7B
OsGA20
oxidase2
7B
Dist
cM
Marker
Name
3.4
2.6
18.2
7.5
1.5
0.9
1.8
3.4
0.0
0.0
3.8
0.0
5.1
2.6
3.2
0.0
1.4
4.7
5.3
14.1
14.8
6.7
5.7
32.2
21.3
3.9
4.7
2.5
4.7
1.4
gwm0537
gwm0400
wmc0076
gwm1184
barc0255
barc0072
gwm0046
barc0267
wmc0471b
wmc0364
wmc0662
gwm0043
gwm0297
gwm0333
gwm0897
gwm0983
gwm0808
wmc0396
barc0176
barc0278
gwm4059
gwm4249a
wmc0517
gwm0767
gwm1144
gwm0783
gwm0577
gwm3132
barc0340
barc0182
wmc0276
Rht13

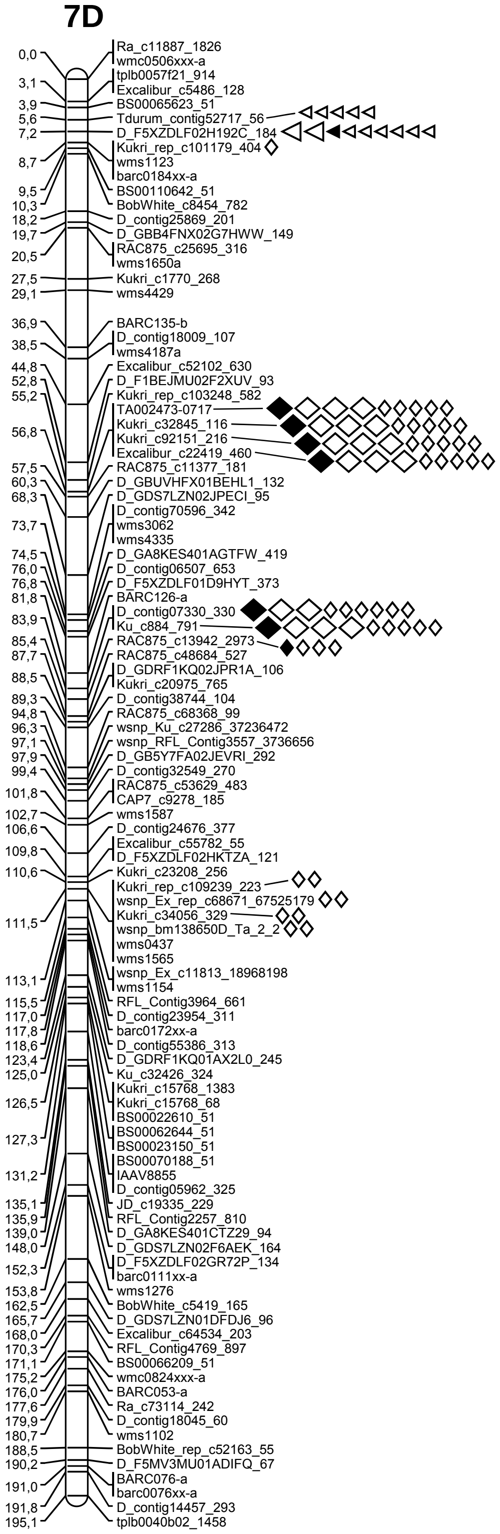
7D
0,0
Ra_c11887_1826
wmc0506xxx-a
3,1
tplb0057f21_914
Excalibur_c5486_128
3,9
BS00065623_51
5,6
Tdurum_contig52717_56
7,2
D_F5XZDLF02H192C_184
8,7
Kukri_rep_c101179_404
wms1123
barc0184xx-a
9,5
BS00110642_51
10,3
BobWhite_c8454_782
18,2
D_contig25869_201
19,7
D_GBB4FNX02G7HWW_149
20,5
RAC875_c25695_316
wms1650a
27,5
Kukri_c1770_268
29,1
wms4429
36,9
BARC135-b
38,5
D_contig18009_107
wms4187a
44,8
Excalibur_c52102_630
52,8
D_F1BEJMU02F2XUV_93
55,2
Kukri_rep_c103248_582
TA002473-0717
56,8
Kukri_c32845_116
Kukri_c92151_216
Excalibur_c22419_460
57,5
RAC875_c11377_181
60,3
D_GBUVHFX01BEHL1_132
68,3
D_GDS7LZN02JPECI_95
73,7
D_contig70596_342
wms3062
wms4335
74,5
D_GA8KES401AGTFW_419
76,0
D_contig06507_653
76,8
D_F5XZDLF01D9HYT_373
81,8
BARC126-a
83,9
D_contig07330_330
Ku_c884_791
85,4
RAC875_c13942_2973
87,7
RAC875_c48684_527
88,5
D_GDRF1KQ02JPR1A_106
Kukri_c20975_765
89,3
D_contig38744_104
94,8
RAC875_c68368_99
96,3
wsnp_Ku_c27286_37236472
97,1
wsnp_RFL_Contig3557_3736656
97,9
D_GB5Y7FA02JEVRI_292
99,4
D_contig32549_270
101,8
RAC875_c53629_483
CAP7_c9278_185
102,7
wms1587
106,6
D_contig24676_377
109,8
Excalibur_c55782_55
D_F5XZDLF02HKTZA_121
110,6
Kukri_c23208_256
111,5
Kukri_rep_c109239_223
wsnp_Ex_rep_c68671_67525179
Kukri_c34056_329
wsnp_bm138650D_Ta_2_2
wms0437
wms1565
wsnp_Ex_c11813_18968198
113,1
wms1154
115,5
RFL_Contig3964_661
117,0
D_contig23954_311
117,8
barc0172xx-a
118,6
D_contig55386_313
123,4
D_GDRF1KQ01AX2L0_245
125,0
Ku_c32426_324
126,5
Kukri_c15768_1383
Kukri_c15768_68
127,3
BS00022610_51
BS00062644_51
BS00023150_51
BS00070188_51
131,2
IAAV8855
D_contig05962_325
135,1
JD_c19335_229
135,9
RFL_Contig2257_810
139,0
D_GA8KES401CTZ29_94
148,0
D_GDS7LZN02F6AEK_164
152,3
D_F5XZDLF02GR72P_134
barc0111xx-a
153,8
wms1276
162,5
BobWhite_c5419_165
165,7
D_GDS7LZN01DFDJ6_96
168,0
Excalibur_c64534_203
170,3
RFL_Contig4769_897
171,1
BS00066209_51
175,2
wmc0824xxx-a
176,0
BARC053-a
177,6
Ra_c73114_242
179,9
D_contig18045_60
180,7
wms1102
188,5
BobWhite_rep_c52163_55
190,2
D_F5MV3MU01ADIFQ_67
191,0
BARC076-a
barc0076xx-a
191,8
D_contig14457_293
195,1
tplb0040b02_1458

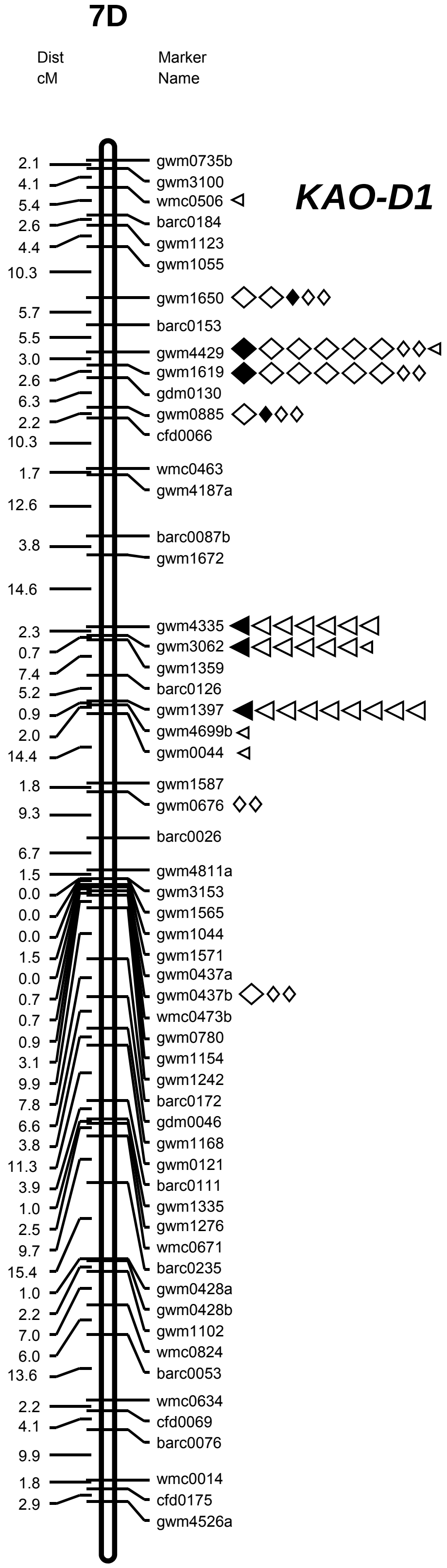
7D
Dist
cM
Marker
Name
KAO-D1
2.1
4.1
5.4
2.6
4.4
10.3
5.7
5.5
3.0
2.6
6.3
2.2
10.3
1.7
12.6
3.8
14.6
2.3
0.7
7.4
5.2
0.9
2.0
14.4
1.8
9.3
6.7
1.5
0.0
0.0
0.0
1.5
0.0
0.7
0.7
0.9
3.1
9.9
7.8
6.6
3.8
11.3
3.9
1.0
2.5
9.7
15.4
1.0
2.2
7.0
6.0
13.6
2.2
4.1
9.9
1.8
2.9
gwm0735b
gwm3100
wmc0506
barc0184
gwm1123
gwm1055
gwm1650
barc0153
gwm4429
gwm1619
gdm0130
gwm0885
cfd0066
wmc0463
gwm4187a
barc0087b
gwm1672
gwm4335
gwm3062
gwm1359
barc0126
gwm1397
gwm4699b
gwm0044
gwm1587
gwm0676
barc0026
gwm4811a
gwm3153
gwm1565
gwm1044
gwm1571
gwm0437a
gwm0437b
wmc0473b
gwm0780
gwm1154
gwm1242
barc0172
gdm0046
gwm1168
gwm0121
barc0111
gwm1335
gwm1276
wmc0671
barc0235
gwm0428a
gwm0428b
gwm1102
wmc0824
barc0053
wmc0634
cfd0069
barc0076
wmc0014
cfd0175
gwm4526a
