# Supplementary material for: Whole Genome Association Mapping of Plant Height in Winter Wheat (Triticum aestivum L.)
Source: PLoS One. 2014 Nov 18;9(11):e113287. doi: 10.1371/journal.pone.0113287 (PMC4236181; doi:10.1371/journal.pone.0113287)
Supplement: Figure S2 — No Linkage disequilibrium was detected between SSR-marker GWM0261 and the candidate gene Ppd-D1 on chromosome 2D. (PDF) [file pone.0113287.s002.pdf]

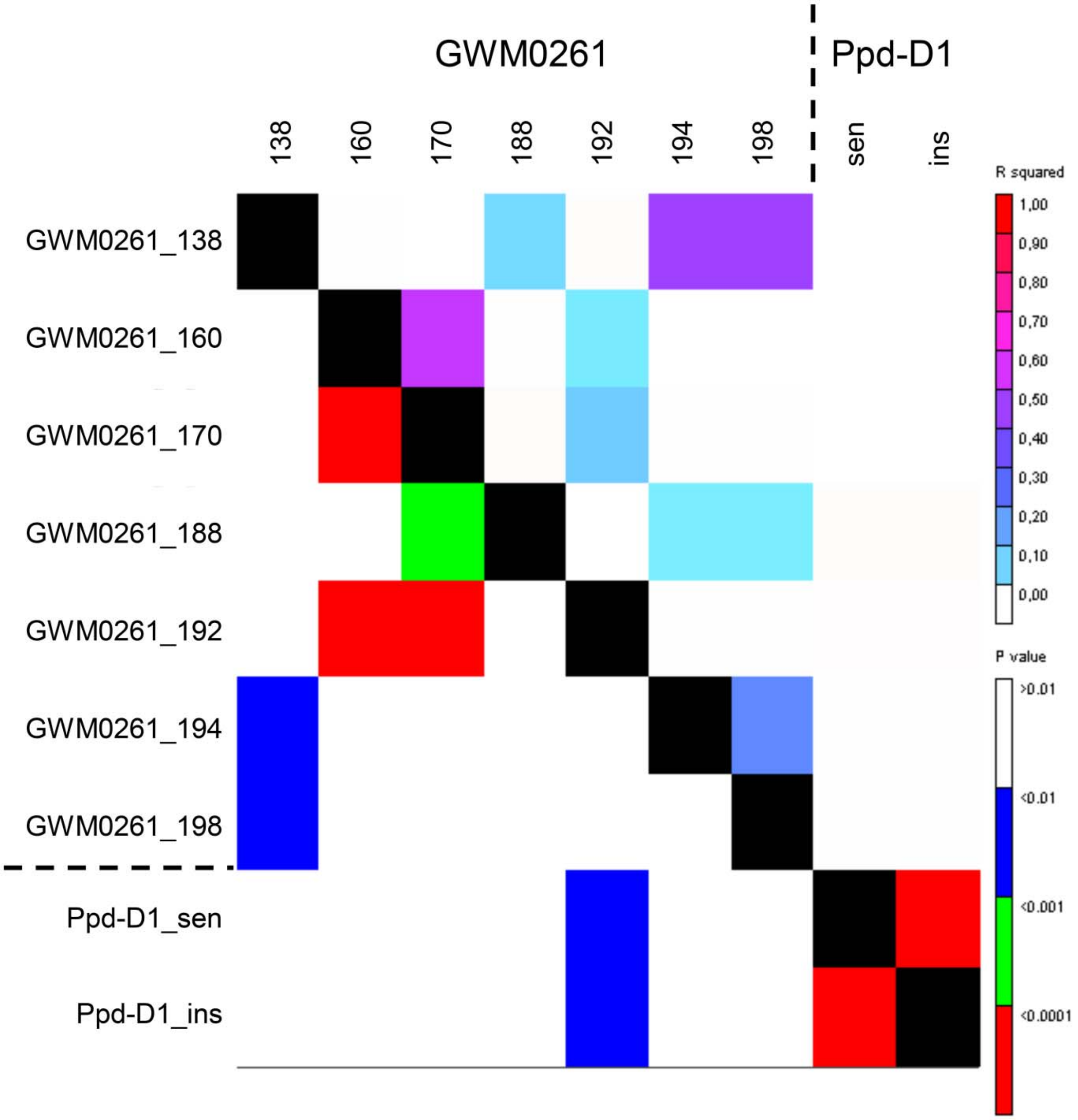

Figure S2: No Linkage disequilibrium was detected between SSR-marker GWM0261 and the candidate gene *Ppd-D1* on chromosome 2D.
