# Supplementary figures and images for: Whole Genome Association Mapping of Plant Height in Winter Wheat (Triticum aestivum L.)
Source: PLoS One. 2014 Nov 18;9(11):e113287. doi: 10.1371/journal.pone.0113287 (PMC4236181; doi:10.1371/journal.pone.0113287)

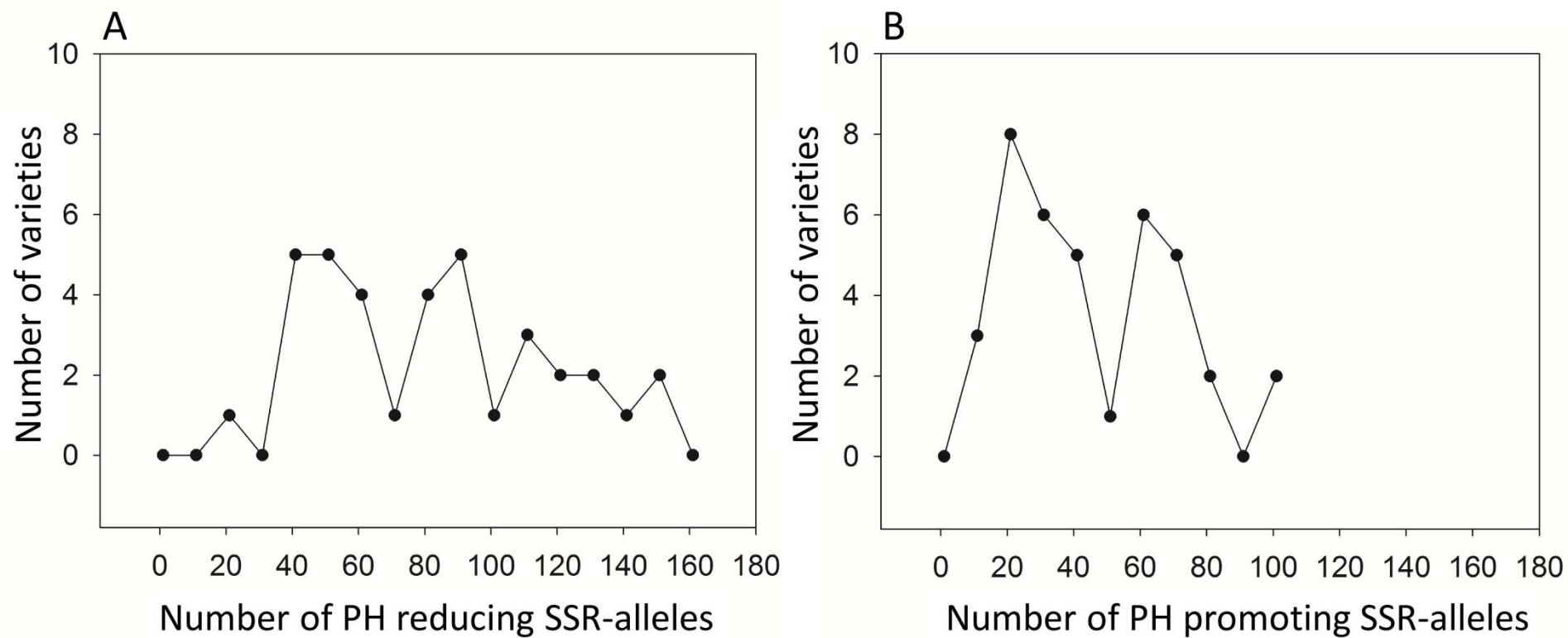

Figure S3: Frequency of (A) PH reducing and (B) PH promoting SSR-alleles per variety.

Supplement: Figure S3 — Frequency of (A) PH reducing and (B) PH promoting SSR-alleles per variety. (PDF) [file pone.0113287.s003.pdf]
