# Supplementary material for: Whole Genome Association Mapping of Plant Height in Winter Wheat (Triticum aestivum L.)
Source: PLoS One. 2014 Nov 18;9(11):e113287. doi: 10.1371/journal.pone.0113287 (PMC4236181; doi:10.1371/journal.pone.0113287)
Supplement: Table S3 — Estimation of variance components and broad sense heritability and estimation of differences between groups ( = environments) using ANOVA and a Tukey B test. (DOCX) [file pone.0113287.s006.docx]

Table S3: Estimation of variance components and broad sense heritability and estimation of differences between groups (= environments) using ANOVA and a Tukey B test.

| **Variance Estimates** | |
| --- | --- |
| Component | Estimate |
| Var(Genotype) | 72.846 |
| Var(Error) | 73.069 |
| Dependent Variable: PH Method: Minimum Norm Quadratic Unbiased Estimation (Weight = 1 for Random Effects and Residual) | |
| H^2^ = 0.882 |  |

| **ANOVA** | | | | | |
| --- | --- | --- | --- | --- | --- |
| **PH** | | | | | |
|  | Sum of Squares | df | Mean Square | F | Sig. |
| Between Groups | 166377.110 | 7 | 23768.159 | 264.004 | .000 |
| Within Groups | 267207.929 | 2968 | 90.030 |  |  |
| Total | 433585.038 | 2975 |  |  |  |

| **PH Posthoc test** | | | | | | | | |  |
| --- | --- | --- | --- | --- | --- | --- | --- | --- | --- |
| Tukey B^a^ | | | | | | | | |  |
| env no | N | Subset for alpha = 0.05 | | | | | | |  |
|  |  | 1 | 2 | 3 | 4 | 5 | 6 | 7 |  |
| 10.SEL.PH Mean | 372 | 74.503 |  |  |  |  |  |  |  |
| 09.WOH.PH Mean | 372 |  | 78.742 |  |  |  |  |  |  |
| 10.WOH.PH Mean | 372 |  |  | 85.497 |  |  |  |  |  |
| 09.SEL.PH Mean | 372 |  |  |  | 87.508 |  |  |  |  |
| 10.JAN.PH Mean | 372 |  |  |  | 87.952 | 87.952 |  |  |  |
| 10.SAU.PH Mean | 372 |  |  |  |  | 89.613 |  |  |  |
| 09.AND.PH Mean | 372 |  |  |  |  |  | 94.188 |  |  |
| 10.AND.PH Mean | 372 |  |  |  |  |  |  | 99.701 |  |
| Means for groups in homogeneous subsets are displayed. | | | | | | | | |  |
| a. Uses Harmonic Mean Sample Size = 372.000. | | | | | | | | |  |
