# Supplementary material for: Whole Genome Association Mapping of Plant Height in Winter Wheat (Triticum aestivum L.)
Source: PLoS One. 2014 Nov 18;9(11):e113287. doi: 10.1371/journal.pone.0113287 (PMC4236181; doi:10.1371/journal.pone.0113287)
Supplement: Table S4 — Spearman rank order correlation of PH scores in 372 varieties among eight environments and the BLUEs. GAT_2012 represents an untreated control environment in a plant nursery. (DOCX) [file pone.0113287.s007.docx]

**Table S4: Spearman rank order correlation of PH scores in 372 varieties among eight environments and the BLUEs.**

GAT_2012 represents an untreated control environment in a plant nursery.

|  | 09.SEL.PH | 09.WOH.PH | 10.AND.PH | 10.JAN.PH | 10.SAU.PH | 10.SEL.PH | 10.WOH.PH | BLUES | GAT_2012 |
| --- | --- | --- | --- | --- | --- | --- | --- | --- | --- |
| 09.AND.PH | 0.932 | 0.929 | 0.936 | 0.871 | 0.909 | 0.919 | 0.942 | 0.968 | 0.871 |
| 09.SEL.PH |  | 0.943 | 0.931 | 0.869 | 0.919 | 0.929 | 0.934 | 0.969 | 0.853 |
| 09.WOH.PH |  |  | 0.918 | 0.862 | 0.905 | 0.916 | 0.923 | 0.960 | 0.834 |
| 10.AND.PH |  |  |  | 0.917 | 0.917 | 0.918 | 0.933 | 0.973 | 0.879 |
| 10.JAN.PH |  |  |  |  | 0.877 | 0.863 | 0.863 | 0.927 | 0.823 |
| 10.SAU.PH |  |  |  |  |  | 0.916 | 0.921 | 0.957 | 0.811 |
| 10.SEL.PH |  |  |  |  |  |  | 0.937 | 0.959 | 0.834 |
| 10.WOH.PH |  |  |  |  |  |  |  | 0.968 | 0.866 |
| GAT_2012 |  |  |  |  |  |  |  | 0.882 |  |
