# Supplementary material for: Whole Genome Association Mapping of Plant Height in Winter Wheat (Triticum aestivum L.)
Source: PLoS One. 2014 Nov 18;9(11):e113287. doi: 10.1371/journal.pone.0113287 (PMC4236181; doi:10.1371/journal.pone.0113287)
Supplement: Table S5 — Analysis of variance (ANOVA) of plant height in 372 varieties in eight environments. (DOCX) [file pone.0113287.s008.docx]

**Table S5: Analysis of variance (ANOVA) of plant height in 372 varieties in eight environments.**

| Source of Variation | DF | SS | MS | F | P |
| --- | --- | --- | --- | --- | --- |
| Genotype | 371 | 243314.261 | 655.834 | 71.282 | <0.001* |
| Env | 7 | 166377.110 | 23768.159 | 2583.358 | <0.001* |
| Residual | 2597 | 23893.668 | 9.200 |  |  |
| Total | 2975 | 433585.038 | 145.743 |  |  |

DF = Degrees of freedom

SS = Sum of Squares

MS = Mean of Squares

***** P<0.001
